# Compositional neurosymbolic representations enable efficient active exploration

**DOI:** 10.1038/s41467-026-75703-4

**Published:** 2026-08-22

**Authors:** P. Michael Furlong, Nicole S.-Y. Dumont, Rika Antonova, Jeff Orchard, Chris Eliasmith

**Affiliations:** 1 Centre for Theoretical Neuroscience, University of Waterloo, Waterloo, Canada; 2 Department of Systems Design Engineering, University of Waterloo, Waterloo, Canada; 3 National Research Council Canada, Ottawa, Canada; 4 Institute of Neuroinformatics, University of Zurich, Zürich, Switzerland; 5 University of Cambridge, Cambridge, United Kingdom; 6 Cheriton School of Computer Science, University of Waterloo, Waterloo, Canada; 7 Department of Philosophy, University of Waterloo, Waterloo, Canada

**Keywords:** Computational science, Learning algorithms

## Abstract

Autonomous systems that learn and explore over long horizons face a problem. Standard methods scale poorly in the number of observations, *n*, precluding sustained operation on bounded hardware. We show that compositional, high-dimensional vector representations inspired by neural computation address these constraints. We use these representations to construct a Bayesian optimization (BO) algorithm that operates in complex spaces and reduces the time and memory requirements compared to state-of-the-art BO algorithms on diverse tasks. Whereas standard methods incur *O*(*n*^3^) time and *O*(*n*^2^) memory complexity, our approach holds both at *O*(*d*^2^) in the embedding dimension, which remains constant over the algorithm’s lifetime. Our algorithm reduces compute time by 60–200 × without loss in accuracy. Implementation on neuromorphic hardware reduces energy consumption per sample by 30–188 × . These efficiencies stem from converting sample selection into continuous optimization on a compact domain, implementable by gradient methods or neural dynamics, enabling long-term, resource-bound, autonomous exploration.

## Abstract (web summary)

Bayesian optimization can guide autonomous exploration but scales poorly, limiting its use on resource-constrained systems. Here, the authors introduce a neuro-symbolic embedding approach that delivers speedup, with partial implementation validated on neuromorphic hardware.

## Introduction

Generalizing efficiently from limited data in complex environments remains a major challenge for artificial intelligence (AI). Humans, in contrast, learn and act in complex, high-dimensional state and action spaces with remarkable efficiency, even in the face of uncertainty. A crucial aspect of this ability is the use of compositional representations, where simpler elements are systematically combined to form more complex structures. This principle underlies human cognition, from perception to language, and is fundamental to generalization and adaptability in complex environments^1–4^. However, achieving both *scalability* and *compositional* expressiveness in AI models remains an open challenge^5,6^.

In machine learning, kernel methods offer a principled way to encode structured inductive biases^7^, support data-efficient learning, and model uncertainty. Compositional kernels, constructed by combining simpler kernels via addition or multiplication, enable the expression of rich relational structures^8^. However, the practical application of such kernels is hindered by scalability: inference with Gaussian processes (GPs) requires ${{{\mathcal{O}}}}({n}^{3})$ time and ${{{\mathcal{O}}}}({n}^{2})$ memory, where *n* is the number of observations. While kernel approximation methods such as Random Fourier Features [RFFs; ^9^] reduce this complexity, approximating compositional kernels with RFFs often leads to unbounded growth in embedding dimensionality^10^, and representing structured or symbolic inputs remains challenging.

Vector Symbolic Algebras (VSAs) - specifically, Holographic Reduced Representations (HRRs) and their complex-valued variant, Fourier HRRs (FHRRs) ^11,12^–offer a promising solution by providing a dimensionality-preserving algebra for compositional representation. We note that VSAs are more commonly known as *vector symbolic architectures*^13^, but we use the term algebra to distinguish the algebras from the systems architected using VSAs.

Vector embeddings used in kernel approximation (such as RFFs) typically implement composition using operations like concatenation (for additive kernels) or tensor products (for multiplicative kernels), both of which increase dimensionality. In contrast, VSAs achieve similar compositionality using fixed-dimensional operations such as bundling and binding (e.g., circular convolution in HRRs). This allows vector embeddings of structured and hierarchical data to have fixed dimensionality.

VSAs have a long history in cognitive science^6,13–21^, and have more recently been used for neural-symbolic deep learning^21,22^, differentiable memory^23^, efficient attention^24,25^, and kernel approximation.

Indeed, the connection between VSAs and kernel embeddings has been well understood since their inception^26^, but has garnered recent attention ^27–31^. The central hypothesis of VSAs is that cognition operates through algebraic manipulations of high-dimensional distributed representations. These operations support not only compositionality and neural implementation^20^, but also the encoding of uncertainty^29,30,32^.

In this work, we introduce Spatial Semantic Pointer Bayesian optimization (SSP-BO), a new Bayesian optimization (BO) algorithm based on VSA-derived kernel approximations. Our method leverages the algebra of HRRs to construct expressive, compositional, and fixed-size embeddings over various action spaces. Additionally, our work further connects BO to biologically plausible neural representations through the use of continuous vector encodings called Spatial Semantic Pointers (SSPs), also referred to as fractional power encodings,^33–35^. These representations reproduce activity patterns observed in the brain’s spatial coding systems–such as grid, place, and head direction cells^36–38^–and serve as our algorithm’s embedding method for continuous variables.

This yields several advantages:

- **Scalability:** We obtain constant-time sample selection with respect to the number of environment observations, *n*. Standard BO requires ${{{\mathcal{O}}}}({n}^{3})$ time and ${{{\mathcal{O}}}}({n}^{2})$ memory for surrogate model updates; our approach reduces this to ${{{\mathcal{O}}}}({d}^{2})$ and ${{{\mathcal{O}}}}({d}^{2})$, respectively, where *d* is the fixed dimensionality of the VSA embeddings. Since *d* is set prior to optimization and remains constant, both sample generation time and memory usage are independent of the number of observations collected during the run, and hence *O*(1) with respect to *n*.
- **Expressivity:** Our method supports structured, symbolic, and mixed discrete-continuous input spaces (e.g., graphs, sets, trajectories) via a single algebraic framework.
- **Neuromorphic compatibility:** Sample selection in SSP-BO occurs in the continuous embedding space. This makes it possible to perform sample selection over structured action spaces on neuromorphic hardware, providing a biologically grounded pathway for efficient decision-making systems.

**Fig. 1:**
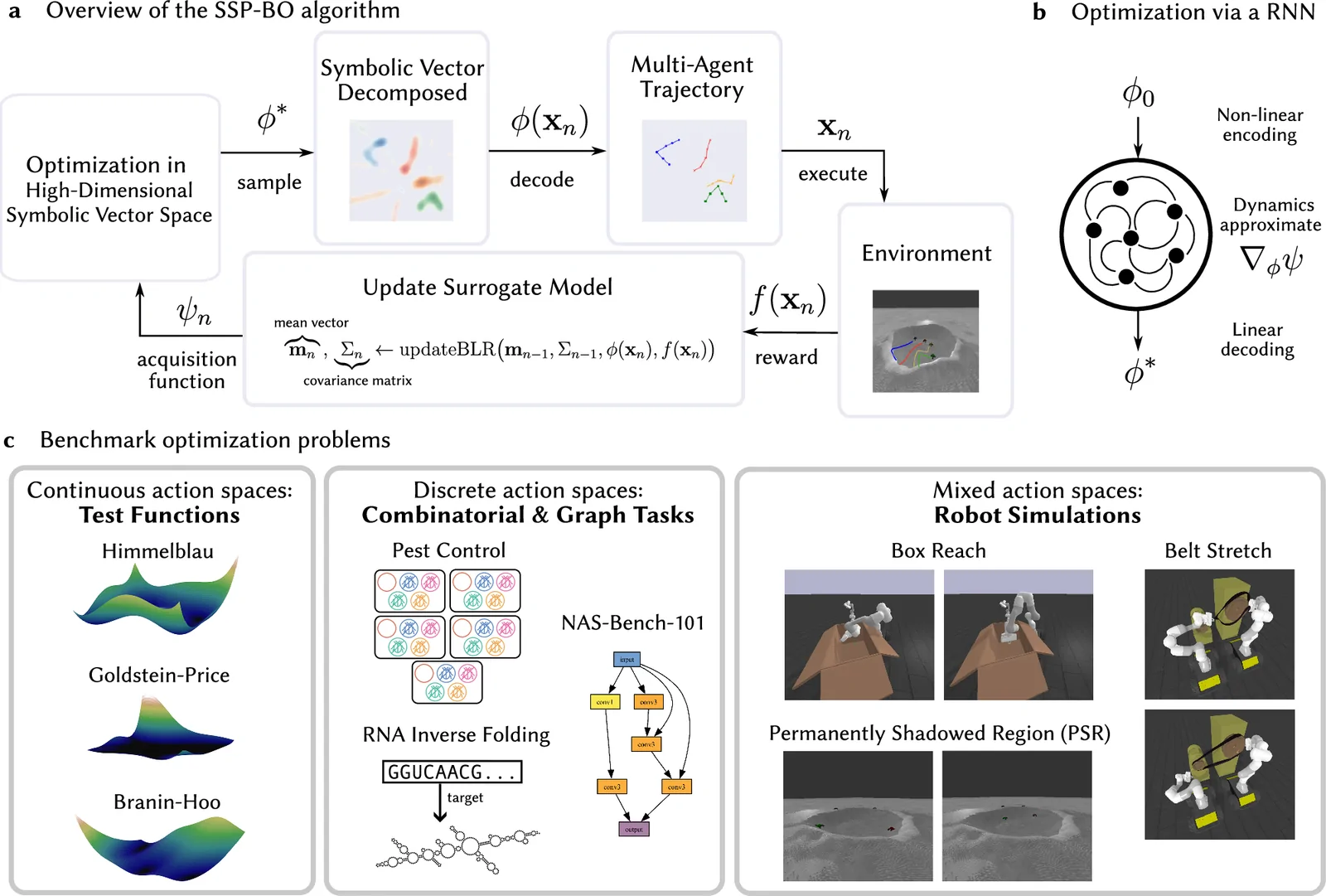
Overview of the SSP-BO method and experiments. **a** Our method conducts optimization in the space of vector symbolic representations. Upon obtaining an candidate vector, we decode it using vector symbolic operators. With that, we obtain an action vector **x**_*t*_, which can be executed in the environment. In this example, an action vector represents desired trajectories of robots searching for water in a crater. We obtain a reward *f*(**x**_*t*_) from the environment and incorporate it into the posterior distribution over the reward function. We update the posterior using Bayesian linear regression. We then optimize an information-theoretic acquisition function in the space of vector symbolic representations, produce the next embedded candidate vector, and so on. This way of conducting optimization yields a method with native support for compositionality and constant time and space complexity. **b** We demonstrate that a recurrent neural population can be used to optimize the objective in the embedding space. Neurons are connected all-to-all, with connection weights set to approximate gradient ascent on the acquisition function. The selected sample $\phi \in {{\mathbb{R}}}^{d}$ is linearly decoded from the population’s activity. **c** The benchmark optimization problems that SSP-BO is tested on. On the left are three standard test functions for global optimization: the Himmelblau, Goldstein-Price, and Branin-Hoo functions. The SSP-BO method was tested on these functions with SSPs used to represent the 2D function input. In the middle are the discrete action space tasks: Pest Control, RNA Inverse Folding and NAS-Bench-101. On the right are the robotics environments: Box Reach, Belt Stretch, and Permanently Shadowed Region (PSR). In Box Reach, the objective is to quickly reach a target within a box. In Belt Stretch, two arms must collaborate to place an elastic belt on a motor and gear assembly. In PSR, multiple robots explore the permanently shadowed of a crater to find water.

We evaluate SSP-BO on a suite of tasks involving action selection in structured domains, including robotic trajectory optimization, multi-agent coordination, graph-based search, and combinatorial design. Compared to exact GP-based methods, SSP-BO offers substantial efficiency gains while maintaining strong performance. Whereas existing approaches typically provide either scalable BO or expressive BO, this work combines both within a single compositional kernel embedding framework, enabling efficient optimization in structured domains and supporting neuromorphic implementation. To the best of our knowledge, this constitutes the first demonstration of neuromorphic hardware being used within a BO loop. A graphical abstract is provided in Fig. 1.

Bayesian optimization (BO) is a data-efficient framework for black-box optimization and reinforcement learning^39–41^. It has been applied to problems in autonomous exploration, morphology and material design, and hyperparameter tuning^42–47^. BO typically models an unknown reward or objective function with a surrogate (often a GP [refs. ^43,48–50^]) and selects queries by optimizing an acquisition function over the surrogate’s posterior. However, the cost of GP inference grows cubically with the number of data points, which limits its applicability in real-time or embedded systems (e.g., spacecraft, edge devices).

Efficiency improvements have been pursued via sparse approximations^51,52^, discretization^42,53,54^, and simulation-based augmentation^55–57^. While effective, these methods either constrain representational flexibility or require significant additional resources (e.g., high-fidelity simulators).

Structured and mixed-domain optimization problems remain especially challenging. Existing approaches often rely on engineered kernels^58,59^ or learned latent space projections^60–64^. However, these methods require training generative models, introduce interpretability challenges, and can be expensive to optimize. While alternatives to GPs exist, the GP formulation remains attractive because of its ease of implementation, convenient theoretical properties, and connection to exploratory behaviors in humans^7,65^.

Positive-definite kernels are closed under addition and multiplication [^66^, section 6.2]. These algebraic operations form the basis for compositional kernels^67,68^, which can be used to encode inductive biases over structured data. This compositionality has been shown to improve generalization in machine learning domains^59^. Furthermore, in cognitive modeling, structured inductive biases reduce the amount of data required for learning^5,69,70^.

In kernel approximation methods, such as RFFs, compositional constructions are generally obtained using concatenation and tensor products of features [e.g.,^10,71–73^]. However, these operations result in feature dimensionality that grows linearly or multiplicatively with composition depth, leading to unbounded resource use as kernel complexity increases. A notable exception is the method proposed by ref. ^74^, which constructs product kernels using element-wise multiplication of complex-valued vectors–a strategy closely related to the FHRR binding operator.

In contrast, VSA-based embeddings used in SSP-BO maintain constant dimensionality through fixed-width algebraic operations, such as bundling and binding (a compressed tensor product). This enables scalable kernel approximation without sacrificing expressivity and supports compositional representations over arbitrary data types. Our method falls within the class of syntactic-driven kernels^58,75^, in which each structured representation induces a corresponding kernel function. Overall, VSAs preserve compositional expressiveness while keeping memory and compute costs bounded – an essential property for neuromorphic implementation.

This paper introduces SSP-BO, a BO algorithm that uses compositional kernel embedding based on VSA-derived representations. In sum, the main contributions of this work are:

- **Using hybrid cognitive representations of continuous trajectories and symbolic entities for BO**. VSAs allows us to address the long-standing problem of efficient BO in combinatorial, continuous, and mixed discrete-continuous spaces by leveraging VSAs with the same algorithm.
- **Employing neuroscience-inspired representations for BO**. We use Hexagonal-Spatial Semantic Pointers (Hex-SSPs), a VSA method for encoding continuous data, used in modeling activity of spatially sensitive neurons found in the hippocampus^36^.
- **Demonstrating constant time and memory sample selection complexity for BO**. SSP-BO reduces the complexity of selecting the next sample during BO from *O*(*n*^3^) and *O*(*n*^2^) in time and memory respectively, to constant in the number of samples collected and *O*(*d*^2^) and *O*(*d*^2^) in the embedding size, which remains fixed during optimization. We empirically support the theoretical claims that SSP-BO’s run time is approximately constant in the number of collected samples, across optimization problems. We show this results in a total sample selection time reduction by a factor of 3.1 − 205.5 × (depending on the total number of samples and the problem size) compared to standard BO methods.
- **Validating the SSP-BO algorithm on neuromorphic computing hardware**. We provide a neural implementation of the sample section in SSP-BO and demonstrate performance on two neuromorphic computing platforms (SpiNNaker and Loihi 2). Notably, this showcases the first implementation of BO on neuromorphic computing platforms.

In requiring only fixed rather than growing resources, our approach enables deploying sophisticated methods for lifelong learning and autonomous exploration on resource-constrained platforms, including neuromorphic hardware. Beyond the specific results reported here, we hope this work illustrates a broader point: that the architectural constraints of neuromorphic and biological systems (fixed resources, compositional structure, constant-time operations) need not limit the sophistication of the algorithms they support, and that established machine learning methods can benefit from the representational frameworks developed in these communities.

## Results

Our algorithm follows a Reinforcement Learning (RL) paradigm: an agent interacts with a (potentially stochastic) environment and seeks the most rewarding actions. The agent can observe the reward function by sampling actions **x** from a domain, ${{{\mathcal{X}}}}$, up to some maximum sampling budget. After receiving an observation, the agent updates its surrogate model of the reward function. There is a need to balance exploration, i.e., searching for more rewarding actions, and exploitation, i.e., using the best rewarded actions seen so far. Actions are selected by optimizing an acquisition function that balances these factors (Equation (18)).

A key aspect of our approach is the use of a Vector Symbolic Algebra (VSA). Actions are mapped to a high-dimensional embedding space, $\phi :{{{\mathcal{X}}}}\to {{\mathbb{R}}}^{d}$, using a VSA. This mapping can be derived for various structured action domains, as explained in the next section. The BO loop using VSA embeddings is presented in section “SSP-BO algorithm” and an overview of SSP-BO is given in Fig. 1. Empirical results begin in section “Experiments”.

### Vector symbolic algebra (VSA)

We introduce VSAs here, but readers interested in greater detail, including how to map them to spiking or non-spiking neural networks, can refer to other resources ^12,20,76–80^. Below we provide the mathematical introduction to VSAs, intuition about the kernels induced by these structures are given in section “Methods”.

**Fig. 2:**
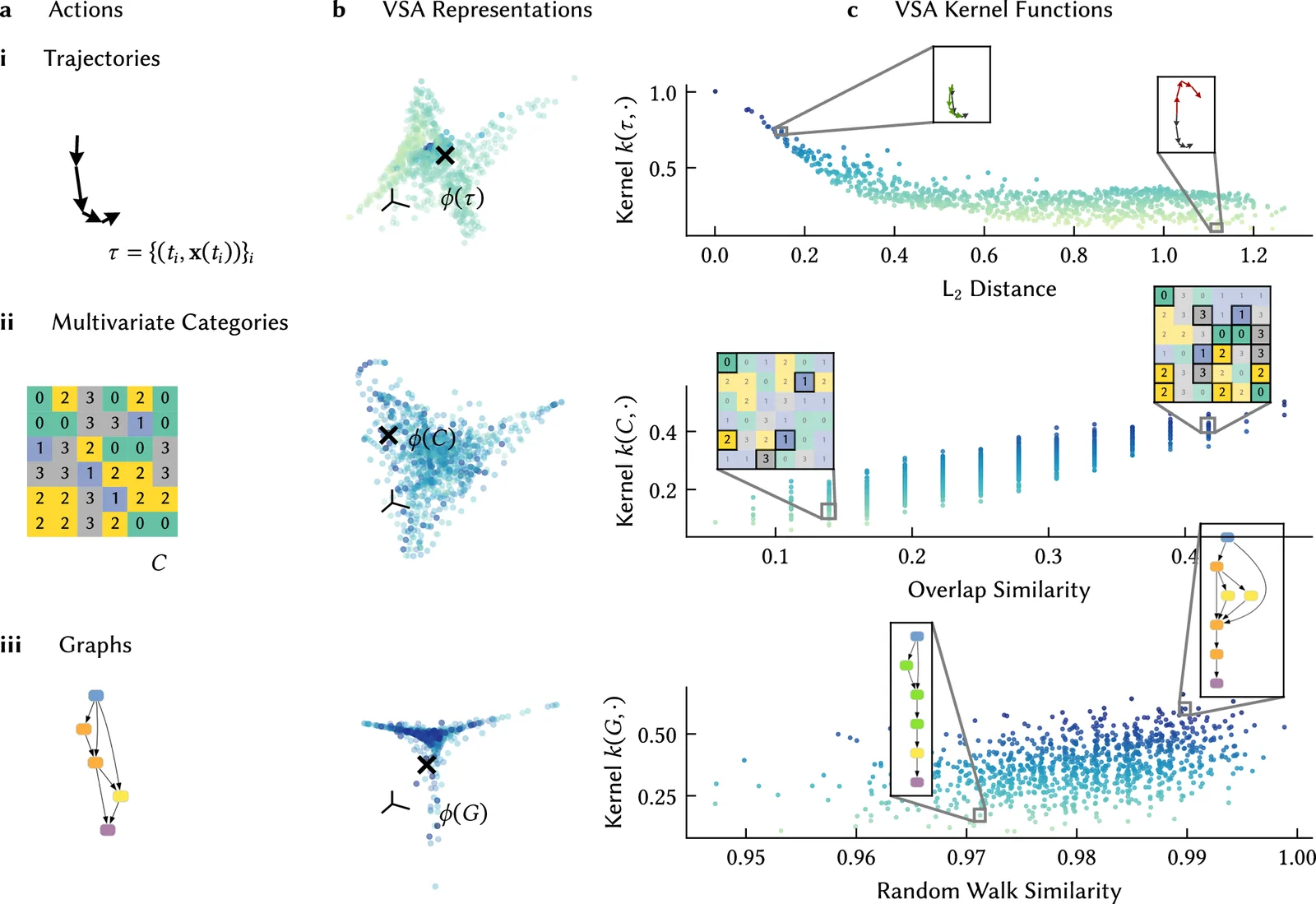
VSA encodings and kernels for different action spaces. **a** Example action spaces used in our optimization tasks, including (**i**) trajectory, (**ii**) combinatorial, and (**iii**) graph-based spaces. A reference action from each space is shown: (**i**) a trajectory *τ* represented as waypoints connected by arrows, (**ii**) a combinatorial variable *C* displayed as a color-coded grid where each square represents a categorical value, and (**iii**) a directed graph representing a convolutional neural network architecture, with nodes as layers (colored by operation type) and arrows as connections^103^. **b** VSA-based representations of randomly sampled actions projected into a 3D space using locally linear embedding for visualization (actual representations are higher-dimensional). The black cross marks the reference action (e.g., *τ*) and colors of other markers indicate the associated kernel value (e.g., *k*(*τ*, ⋅ )). **c** The kernel values under VSA encodings. Each point represents a randomly sampled action, with insets showing example actions (in (**i**) the reference trajectory is plotted underneath in black and in (**ii**) overlap with the reference variable is shown). The y-axis corresponds to the cosine similarity between the VSA representation of the reference action (from **a**) and other actions, while the x-axis corresponds to a standard distance or similarity measure specific to each action space: (**i**) *L*_2_ distance between trajectories, (**ii**) categorical overlap similarity, and (**iii**) a random walk similarity measure for graphs. These results show that VSA-based embeddings induce meaningful similarity measures aligned with known metrics in each space.

VSAs are algebras for capturing symbolic reasoning in high-dimensional vector spaces. In VSAs, symbols, represented as high-dimensional vectors (Fig. 2), can be manipulated by operations on those vectors. Cognitive models are constructed by combining vectors and operators, which can then be used to process stimuli. VSAs are typically equipped with four key operators: *bundling* ( + ), *similarity* ( ⋅ ), *binding* ( ⊗ ), and *unbinding* ( ⊘ ). Some VSAs also define a *hiding* operator, which can be implemented using permutation matrices. HRRs, however, do not include a distinct hiding operator, as that can be achieved by binding. In this paper, we work with Holographic Reduced Representations (HRRs), a type of VSA developed by ref. ^12^. In this framework, *bundling* is vector addition, *similarity* is cosine similarity, *binding* is circular convolution, and *unbinding* is circular correlation, which can be approximated by circular convolution with the pseudo inverse of a vector. Given a convolutional binding operator, it is common to improve the efficiency of the binding and unbinding operations by conducting them in the Fourier domain. Indeed, Fourier HRRs are a variation on HRRs that is conducted entirely in the Fourier domain, and has been used in a number of neuromorphic applications^34,81,82^.

It is sometimes necessary to represent indivisible concepts or objects that may serve as a referent to some high-level concept without defining its constituent elements. We could represent a concept or category, e.g., “retirement” or “animal”, by creating a discrete symbol that the system treats as having the semantic content of the concept it points to. Here discrete symbols are represented by vectors on the unit hyper-sphere. These are generated randomly by selecting their frequency components in the Fourier domain, and then converting them to the psuedo-time domain through the inverse Fourier transform. This differs from the original proposition of Plate^83^ for real-valued HRR symbols, who specified that vectors should be drawn from the distribution, ${{{\mathcal{N}}}}(0,d)$. However, the representations we use for discrete symbols are equivalent to the inverse Fourier transform of Plate’s “circular vectors” with the addition of conjugate symmetry.

Specifically, we generate a phase vector, ${{{\boldsymbol{a}}}}\in {{\mathbb{R}}}^{d}$, where elements of ***a*** are sampled uniformly from the interval [ − *π*, *π*]. Other sampling distributions can be used to generate ***a***, or it may be set some other way, e.g., end-to-end learning. We further constrain ***a*** to have conjugate symmetry, to ensure the inverse Fourier transform does not generate imaginary components, and to be unit-length in the time domain, which places us in a strict subset of HRRs. We define our embedding of the symbol, $s\in {{{\mathcal{S}}}}$, in the high-dimensional space as:

1 $${\phi }_{{{{\mathcal{S}}}}}(s)={{{{\mathcal{F}}}}}^{-1}\left\{{{{{\rm{e}}}}}^{i{{{\bf{a}}}}}\right\},$$

where ${{{{\mathcal{F}}}}}^{-1}$ indicates the inverse Fourier transform, and the exponential function is applied to each element of *i***a**. The kernel induced under this mapping is given in section “Kernels over Discrete Encodings”.

To represent multiple entities, like symbols indicating the identities of several agents, we generate a unique **a**_*k*_ for every entity. Thus, for a set of discrete symbols, ${{{\mathcal{S}}}}=\left\{{s}_{1},\ldots,{s}_{M}\right\}$, we generate a corresponding set of phase vectors, $\left\{{{{{\bf{a}}}}}_{1},\ldots,{{{{\bf{a}}}}}_{M}\right\}$. Discrete symbol vectors play multiple roles within VSAs. They can be used to represent the values of categorical data, but they can also be used to impose structure on a vector representation.

Chiefly, structure is imposed by using discrete symbol vectors to define the *slot* in *slot-filler* relationships. By analogy to programming languages, slots are like the names of variables, and the fillers are the values those variables take on. For example, sentences can be represented in a slot-filler manner with slots such as “subject” or “verb” specifying the functional roles of particular discrete data, such as “Bob” or “throw,”

2 $$\,{\mbox{subject}}\otimes {\mbox{Bob}}+{\mbox{verb}}\otimes {\mbox{throw}}+{\mbox{object}}\otimes {\mbox{ball}}\,.$$

More generally, consider a multi-category or combinatorial variable *C* (e.g., an object with multiple properties that take on discrete values). There is a set of category slots ${\phi }_{{{{\mathcal{S}}}}}({s}_{i})$ (e.g., *s*_1_ = color and *s*_2_ = classification) each of which has an associated value ${\phi }_{{{{{\mathcal{V}}}}}_{i}}({v}_{i,j})$ (e.g., the possible colors are *v*_1,1_ = red, *v*_1,2_ = blue, …  and possible classifications are *v*_2,1_ = door, *v*_2,2_ = box, …  etc.). The mappings are labeled with ${{{\mathcal{S}}}}$ and ${{{{\mathcal{V}}}}}_{i}$ to indicate that there are different dictionaries for category slots and value types. The complete combinatorial variable *C* can be represented as

3 $${\phi }_{{{{\mathcal{C}}}}}(C)={\sum}_{i=1}^{{\#} \, {{{\rm{of}}}} \, {{{\rm{categories}}}}}{\phi }_{{{{\mathcal{S}}}}}({s}_{i})\otimes {\phi }_{{{{{\mathcal{V}}}}}_{i}}({v}_{i,j})+{ \kappa } {\bigotimes}_{i=1}^{{\#} \,{{{\rm{of}}}} \, {{{\rm{categories}}}}}{\phi }_{{{{{\mathcal{V}}}}}_{i}}({v}_{i,j}).$$

The first term bundles all bound slot-filler pairs, *κ* = 0.1 is a weighting constant, and the second term is the bound combination of all values.

As discussed later in section “Data Structures as Approximate Compositional Kernels”, purely bundling variables restricts the representation to additive kernels. To represent multiplicative kernels and capture interactions between input dimensions, the all-bound term is required. This could be generalized to sum over n-gram bound groups, assuming the category slots are ordered in a meaningful way^84,85^. An example of multi-category embeddings is shown in Fig. 2**(ii)**.

VSAs have been previously used to encode graphs^78,86,87^. In section “Experiments”, we consider an optimization problem defined over directed acyclic graphs with *M* labeled nodes, which we represent by

4 $${\phi }_{{{{\mathcal{G}}}}}(G)=	\frac{1}{M}{\sum}_{i=1}^{M}{\phi }_{{{{\mathcal{N}}}}}({n}_{i})\otimes \left(\,{{\mbox{node}}}\_{{\mbox{label}}}\,\otimes {\phi }_{{{{\mathcal{L}}}}}({l}_{i})\,+\,\,{{\mbox{tail}}}\bigotimes \sum \limits_{j{{\rm{{\,where}}}}\,i\to j}{\phi }_{{{{\mathcal{N}}}}}({n}_{j})\right) \\ 	+\,\kappa {{ \otimes }_{i=1}^{M}} {\phi }_{{{{\mathcal{N}}}}}({n}_{i}) \otimes {\phi }_{{{{\mathcal{L}}}}}({l}_{i}) \otimes {\sum}_{j\,{{{\rm{where}}}}\,i \to j}{\phi }_{{{{\mathcal{N}}}}}({n}_{j}),$$

where tail and node_label are slot vectors, ${n}_{i},{n}_{j}\in {{{\mathcal{N}}}}$ denote nodes, and ${l}_{i}\in {{{\mathcal{L}}}}$ is a label associated with the vertex. Nodes disconnected from all others are removed from the graph. Note that, like the multi-category embeddings, there is an all-bound term weighted by a constant *κ* = 0.1.

Moving from discrete symbol representations to discrete ordered representations can be thought of as constructing vector representations of integers. An integer, $j\in {{{\mathcal{J}}}}\subseteq {{{\mathcal{Z}}}}$, can be represented by first selecting a basis vector ${{{{\mathcal{F}}}}}^{-1}\left\{{{{{\rm{e}}}}}^{i{{{{\bf{a}}}}}_{{{{\rm{J}}}}}}\right\}$, a randomly generated symbol as described in the previous section (i.e., **a**_J_ is a random phase vector). To represent an integer, *j*, the basis vector is bound with itself *j* times. We denote this as:

5 $${\phi }_{{{{\rm{J}}}}}(j)={\otimes }^{j}{{{{\mathcal{F}}}}}^{-1}\left\{{{{{\rm{e}}}}}^{i{{{{\bf{a}}}}}_{{{{\rm{J}}}}}}\right\}={{{{\mathcal{F}}}}}^{-1}\left\{{{{{\rm{e}}}}}^{i{{{{\bf{a}}}}}_{{{{\rm{J}}}}}\cdot j}\right\}.$$

In contrast to discrete symbols, integer representations are used to represent values that are ordered and have a meaningful distance (and hence similarity) metric.

To extend iterated binding to real-valued data we exploit the fact that convolution in the time domain is multiplication in the Fourier domain. Integer representations are trivially extended to real-valued data to produce a method known as *fractional binding*^33,35^ or *fractional power encoding*^29,34^. Because some of these methods were developed in the context of the Semantic Pointer Architecture^19^, they are sometimes known as Spatial Semantic Pointers (SSPs)^35^. We can thus represent real-valued data $x\in {{{\mathcal{X}}}}\subseteq {\mathbb{R}}$ as

6 $${\phi }_{{{{\mathcal{X}}}}}(x)={{{{\mathcal{F}}}}}^{-1}\left\{{{{{\rm{e}}}}}^{i{{{{\bf{a}}}}}_{{{{\mathcal{X}}}}}\frac{x}{\ell }}\right\},$$

where $\ell \in {{\mathbb{R}}}^{+}$ is the length scale of the representation, ${{{{\bf{a}}}}}_{{{{\mathcal{X}}}}}$ is the phase vector, and the exponentiation is applied element-wise, as above. This is similar to the method of RFFs^9^, in that it maps scalar inputs to a higher-dimensional feature space via complex exponentials of linear projections. However, unlike RFFs these representations are used with the HRR algebra to create embeddings of compositional structures.

Provided that two variables are bound using the same basis vector, the dot product between fractional bound representations induces a kernel, $k({{{\bf{x}}}},{{{{\bf{x}}}}}^{{\prime} })={\phi }_{{{{\mathcal{X}}}}}{({{{\bf{x}}}})}^{T}{\phi }_{{{{\mathcal{X}}}}}({{{{\bf{x}}}}}^{{\prime} })$. The expected shape of the kernel is a function of the sampling distribution for the phase vector components. Thus, the similarity between continuous variables can be tuned to the needs of a particular application, as typically done with RFFs. In the case of (*a*)_*i*_ ~ Uniform[ − *π*, *π*], the induced kernel is the sinc function^27^. This is a useful kernel because it converges to a point mass in the small bandwidth limit, i.e., sinc((*i* − *j*)/*l*) → *δ*(*i* − *j*) as *l* → 0.

This encoding can be extended to *m* − dimensional data, ${{{\bf{x}}}}\in {{{\mathcal{X}}}}\subseteq {{\mathbb{R}}}^{m}$, by replacing the phase vector, ${{{{\bf{a}}}}}_{{{{\mathcal{X}}}}}$, with a phase matrix, ${{{{\bf{A}}}}}_{{{{\mathcal{X}}}}}\in {{\mathbb{R}}}^{d\times m}$,

7 $${\phi }_{{{{\mathcal{X}}}}}({{{\bf{x}}}})={{{{\mathcal{F}}}}}^{-1}\left\{{{{{\rm{e}}}}}^{i{A}_{{{{\mathcal{X}}}}}{{{\bf{x}}}}}\right\}.$$

In this case, the phase matrix is selected such that the columns are independently generated random vectors. We refer to such an encoding scheme as Random SSPs (Rand-SSPs).

#### Hexagonal SSPs

In this work, we use a phase matrix that has been structured in a particular way. Specifically, the phase matrix is set so that the induced kernel is a sum of periodic kernels that mimic the neural activity of grid cells in the medial entorhinal cortex^36^. We have chosen this structure because it allows SSPs to be efficiently represented by neural populations and, as features for deep networks, outperforms Rand-SSPs on a number of classification and regression tasks^36,88,89^. We call the resulting feature representation Hexagonal SSPs (Hex-SSPs). Section “Hexagonal SSPs” provides the details of the Hex-SSP construction and a derivation of the kernel induced under this embedding.

If we consider the space of trajectories, ${{{\mathcal{T}}}}$, we can represent a trajectory *τ* = [**x**(*t*)]_*t*∈[0, *T*]_ with SSPs as a bundle of time and location representations bound together in a single vector^90^:

8 $${\phi }_{{{{\mathcal{T}}}}}(\tau )=\int_{0}^{T}{\phi }_{{{{\mathcal{X}}}}}({{{\bf{x}}}}(t))\otimes {\phi }_{T}(t)dt,$$

where *t* is time and *T* is the maximum trajectory duration. Pragmatically, instead of computing the exact integral over the trajectory *τ*, we often sample the trajectory and encode a bundle of waypoints bound with time:

9 $${\phi }_{{{{\mathcal{T}}}}}(\tau )={\sum}_{i=1}^{n}{\phi }_{{{{\mathcal{X}}}}}({{{\bf{x}}}}({t}_{i}))\otimes {\phi }_{T}({t}_{i}).$$

If we want to represent trajectories of multiple agents, we can generate random symbol vectors for each agent, bind them to the corresponding trajectory embeddings, and represent the collection in one bundled vector.

10 $${\sum}_{i=1}^{\#\,{{{\rm{of}}}}\, {{{\rm{agents}}}}}{\phi }_{{{{\rm{agts}}}}}({s}_{i})\otimes {\phi }_{{{{\mathcal{T}}}}}({\tau }_{i}),$$

where *ϕ*_agts_(*s*_*i*_) are randomly generated symbol-vectors for the agents whose trajectories are being optimized.

The encoding in Equation (10) serves as the projection into the feature space used in the experiments presented in this work. Note that regardless of the trajectory length or the number of agents involved, the dimensionality of the SSP vector representation does not change.

### SSP-BO algorithm

#### Algorithm 1

SSP Bayesian Optimization (SSP-BO). *N* is the total sampling budget for the algorithm, *β* is the inverse variance parameter, *α* was chosen to be 0.01, *f*( ⋅ ) is the target function being optimized, and ${{{\mathcal{X}}}}$ is the domain of *f*( ⋅ ). *γ*_*c*_ is a scaling parameter that can be used to further control the influence of the exploration term.

1: **Procedure** SSP-BO ($N,f(\cdot ),\frac{1}{\beta },\alpha,\lambda$)

2:  $${{{{\bf{m}}}}}_{0},\,{\Sigma }_{0},\,{\Sigma }_{0}^{-1}\leftarrow {{{\bf{0}}}},\,\alpha {{{\bf{I}}}},\,{\alpha }^{-1}{{{\bf{I}}}}$$

3:  **for** *k* ← 1 to *N* **do**

4:   *μ*_*n*_(*θ*) ≜ **m**_*n*_ ⋅ *θ*

5:   $${\sigma }_{n}(\theta )\triangleq \sqrt{\frac{1}{\beta }+{\theta }^{{{{\rm{T}}}}}{\Sigma }_{n-1}\theta }$$

6:   $${\theta }^{*}\leftarrow \arg {{\max }_{\theta \in {{{\mathcal{S}}}}^{d-1}}}\,\left[{\mu }_{n}(\theta )+{\lambda }_{n}{\sigma }_{n}(\theta )\right]$$

7:   ${{{{\bf{x}}}}}_{n}\leftarrow \arg {{\max }_{{{{\bf{x}}}}\in {{{\mathcal{X}}}}}}\,\phi ({{{\bf{x}}}})\cdot {\theta }^{*}$ ⊳ Decode optimal SSP

8:   *f*_*n*_ ← *f*(**x**_*n*_)

9:  $${{{{\bf{m}}}}}_{n},{\Sigma }_{n},{\Sigma }_{n}^{-1}\leftarrow {{{\rm{updateBLR}}}}\left({{{{\bf{m}}}}}_{n-1},{\Sigma }_{n-1},{\Sigma }_{n-1}^{-1},{{{{\bf{x}}}}}_{n},{f}_{n}\right)$$⊳See eqs. (14)–(16)

10:  **end for**

11: **end procedure**

SSP-BO follows the standard BO framework, adapted to operate over VSA representations (section “Kernel Approximations with VSAs”). At each iteration, the agent selects an action ${{{{\bf{x}}}}}_{n}\in {{{\mathcal{X}}}}$ by optimizing an acquisition function, observes a reward *f*(**x**_*n*_), and updates its surrogate model. This process repeats until a sampling budget *N* is exhausted.

#### Surrogate model

**Fig. 3:**
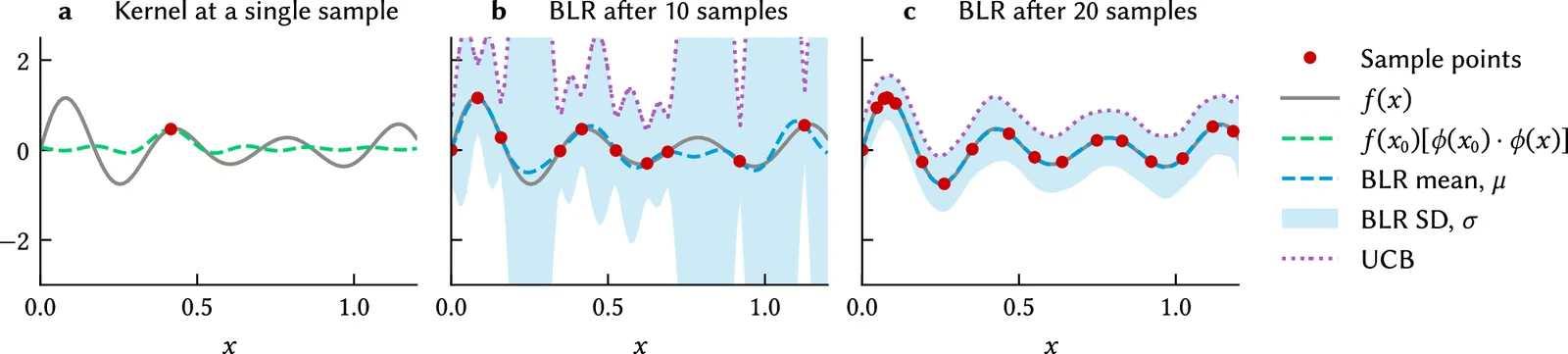
Function posterior estimated using BLR and SSP encoding as the number of samples increases. Red dots indicate sample points, the blue line indicates the estimated function mean, the shaded regions indicate the prediction standard deviation, and the gray line is the true function. **a** The estimated function using only one observation, where uncertainty is undefined. **b** After ten samples the mean begins to approach the true function, although the uncertainty is high. **c** With 20 samples, we obtain an accurate function estimate with reasonable uncertainty bounds.

We model the reward function using Bayesian linear regression (BLR; Fig. 3), which provides a probabilistic surrogate over VSA embeddings. Each action **x** is mapped to a fixed-dimensional vector $\phi ({{{\bf{x}}}})\in {{\mathbb{R}}}^{d}$, and the predicted reward is modeled as a Gaussian distribution:

11 $$\hat{f}({{{\bf{x}}}}) \sim {{{\mathcal{N}}}}(\mu ({{{\bf{x}}}}),{\sigma }^{2}({{{\bf{x}}}})).$$

The parameters of this distribution are computed using standard BLR formulas [^66^ Section 3.3]:

12 $${\mu }_{n}({{{\bf{x}}}})={{{{\bf{m}}}}}_{n-1}^{T}\phi ({{{\bf{x}}}}),$$

13 $${\sigma }_{n}^{2}({{{\bf{x}}}})=\frac{1}{\beta }+\phi {({{{\bf{x}}}})}^{T}{\Sigma }_{n-1}\phi ({{{\bf{x}}}}),$$

where *μ*_*n*_(**x**) is the posterior mean of the predicted reward at **x**, parameterized by the mean vector **m**_*n*−1_, and ${\sigma }_{n}^{2}({{{\bf{x}}}})$ is the posterior variance, parameterized by the covariance matrix *Σ*_*n*−1_. The precision parameter *β* is set as the inverse variance of the observed rewards and is estimated from the initial samples. The mean vector and covariance matrix are updated incrementally with each new observation.

#### Online updates

We use rank-one updates to update the inverse covariance matrix ${\Sigma }_{n}^{-1}$ and thus update the covariance matrix *Σ*_*n*_ online via the matrix inversion lemma (Equation (15)), avoiding costly inversions. Online updates to the BLR parameters are given by

14 $${\Sigma }_{n}^{-1}={\Sigma }_{n-1}^{-1}+\beta \phi ({{{{\bf{x}}}}}_{n})\phi {({{{{\bf{x}}}}}_{n})}^{T},$$

15 $${\Sigma }_{n}={\Sigma }_{n-1}-\frac{{\Sigma }_{n-1}\phi ({{{{\bf{x}}}}}_{n})\phi {({{{{\bf{x}}}}}_{n})}^{T}{\Sigma }_{n-1}}{\frac{1}{\beta }+\phi ({{{{\bf{x}}}}}_{n}){\Sigma }_{n-1}\phi {({{{{\bf{x}}}}}_{n})}^{T}}\,,$$

16 $${{{{\bf{m}}}}}_{n}={\Sigma }_{n}{\Sigma }_{n-1}^{-1}{{{{\bf{m}}}}}_{n-1}+{\Sigma }_{n}\beta \phi ({{{{\bf{x}}}}}_{n})f({{{{\bf{x}}}}}_{n}).$$

#### Acquisition function

Actions are selected by maximizing an acquisition function, *ψ*_*n*_(**x**). We use the Upper Confidence Bound [UCB; ^91–94^] criterion to balance exploration and exploitation. The acquisition function at iteration *n* is:

17 $${\psi }_{n}({{{\bf{x}}}})={\mu }_{n}({{{\bf{x}}}})+\lambda {\sigma }_{n}({{{\bf{x}}}})$$

18 $$={{{{\bf{m}}}}}_{n-1}^{{{{\rm{T}}}}}\phi ({{{\bf{x}}}})+\lambda \sqrt{\frac{1}{\beta }+\phi {({{{\bf{x}}}})}^{{{{\rm{T}}}}}{\Sigma }_{n-1}\phi ({{{\bf{x}}}})}.$$

where *λ* = 1 is a scalar that governs the trade-off between uncertainty-driven exploration and predicted reward.

#### Optimization in embedding space

To select the next action, we optimize the acquisition function in the high-dimensional VSA space:

19 $$\,{\mbox{Optimize:}}\,\quad {\theta }^{*}={{{{\rm{arg}}}\; {{\rm{max}}}}_{\theta \in {{{{\mathcal{S}}}}}^{d-1}}}\,{{{{\bf{m}}}}}_{n-1}^{{{{\rm{T}}}}}\theta+\lambda \sqrt{{\beta }^{-1}+{\theta }^{{{{\rm{T}}}}}{\Sigma }_{n-1}\theta },$$

20 $$\,{\mbox{Decode:}}\,\quad {{{{\bf{x}}}}}_{n}={{{{\rm{arg}}}\;{{\rm{max}}}}_{{{{\bf{x}}}}\in {{{\mathcal{X}}}}}}\ {\theta }^{*}\cdot \phi ({{{\bf{x}}}}).$$

where ${{{{\mathcal{S}}}}}^{d-1}$ is the *d*-dimensional unit hypersphere. Note that θ^*^ may not correspond exactly to any valid action embedding – i.e., θ^*^ might not lie in the set $\{\phi ({{{\bf{x}}}}):{{{\bf{x}}}}\in {{{\mathcal{X}}}}\}$. Thus, we decode it by selecting the action whose embedding is most similar in feature space. This decoding step is conceptually similar to projection methods in constrained optimization, but in our case is based on similarity rather than geometric projection.

By operating in the embedding space, SSP-BO transforms a complex optimization problem in a potentially structured or discrete action space, ${{{\mathcal{X}}}}$, into a continuous optimization problem on ${{{{\mathcal{S}}}}}^{d-1}$, at the cost of approximation. The objective is smooth and defined on a compact domain, lending itself to efficient optimization via gradient ascent or neural dynamics. This formulation also lends itself to neuromorphic implementation, as the same recurrent neural architecture can perform sample selection across diverse data types and structures (e.g., discrete, continuous, sets, graphs, and trajectories). However, the task of decoding θ^*^ into an appropriate action, x^*^, can be challenging, see section “Feature Decoding” for more details.

The complete algorithm is given in Algorithm 1.

### Experiments

We now demonstrate that VSA-based representations, when used in the acquisition function Equation (18), support effective optimization. We compare SSP-BO against various BO baselines that optimize directly in the action space ${{{\mathcal{X}}}}$.

Our experiments span a range of action types and problem structures (Fig. 1(c)). First, we evaluate SSP-BO on continuous function optimization using the Hex-SSP representation. Second, we test SSP-BO on discrete action spaces, including combinatorial optimization and a neural architecture search problem. These tasks assess whether VSA representations support efficient optimization over symbolic and structured domains. Third, we evaluate SSP-BO on multi-agent trajectories, demonstrating the method’s ability to optimize in mixed, high-dimensional spaces.

In all domains, we find that SSP-BO performs better than or comparably to BO baselines. We also conduct runtime profiling and complexity analysis, showing that SSP-BO yields significant computational advantages due to its fixed-size embedding and efficient updates. Finally, we demonstrate that the algorithm can be implemented on neuromorphic hardware using spiking neurons, highlighting its potential for energy-efficient, brain-inspired optimization.

Previously, randomly generated SSPs have been used as a feature space for BO^95^. In this work, we show that Hex-SSP features are more effective than Rand-SSP features for BO, although both can be used. Because we test these algorithms on standard target functions, we know what the maximum value of these functions is, so we can score the algorithms on the basis of regret. Regret is the difference between the reward obtained, *f*(*x*_*n*_), and the best reward, *f*(x^*^). We report the average regret at sample number *n*, ${R}_{n}=\frac{1}{n}{\sum }_{i=1}^{n}f({x}^{*})-f({x}_{n})$. Lower values of this quantity are better.

**Fig. 4:**
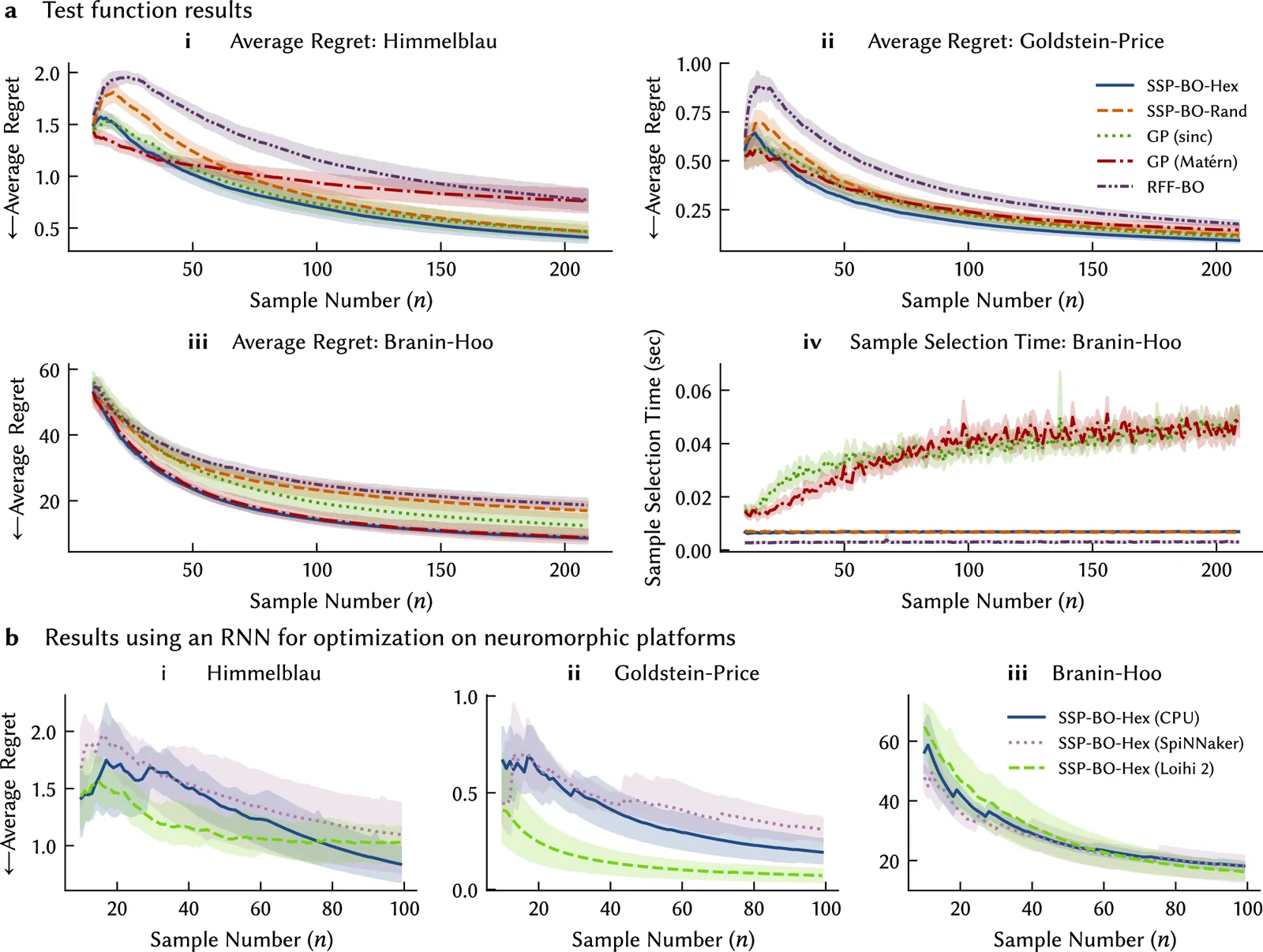
SSP-BO offers bounded execution time and sample selection on neuromorphic hardware, while achieving regret comparable to standard implementations. **a** Plots **(i–iii)** show the average regret *R*_*t*_ (lower is better); plot **(iv)** shows the sample selection time (lines 4-7 and 9 in Algorithm 1), which includes the decoding step and does not include the function evaluation time. Shaded regions are 95% bootstrap confidence intervals for 30 trials with sampling budgets of *N* = 200. There are an additional 10 random samples that are used to initialize the surrogate models. This stage is not included in the plots, but the initial samples do contribute to the computed regret. SSP-BO-Hex achieves better or equal regret performance to GP (Matérn) while showing a substantial improvement in selection time. **b** Results when sample selection is performed by an spiking RNN. Plots **(i–iii)** show the average regret and 95% bootstrap confidence intervals for 5 trials with sampling budgets of *N* = 100 using different hardware to simulate the RNN.

Figure 4a shows the performance of SSP-BO on three common global optimization functions, as well as the time complexity of the algorithms compared to a baseline algorithm implemented using GP and a GP approximated with RFFs (see section “Baseline Implementations for BO” and Supplementary Note 3, “Baseline Methods” for details). As anticipated, SSP-BO has constant time complexity, and constant memory complexity (see Supplementary Fig. 1). Note that the time for RFF-BO is also constant, as it too uses a kernel approximation. It is in fact slightly faster than SSP-BO because RFF-BO optimizes in the original action space and therefore does not require a decoding step. However, RFF-BO has higher regret on all test functions. SSP-BO with Hex-SSPs achieves the best regret performance. Furthermore, we find that the Hex-SSP results were stable even when reduced to 25 dimensions (Supplementary Fig. 3).

We evaluate our approach on two combinatorial optimization tasks: a synthetic pest control task^96^ and an RNA inverse folding task^97^. For comparison, we use benchmark results from ^98^, which include various GP-based Bayesian optimization methods such as COMBO^96^, BOiLS^99^, BODi^100^, and BOSS^101^. Each method employs different combinatorial kernels and optimization algorithms that operate directly in the combinatorial action space (see Supplementary Table II). Some methods use computational strategies to manage the cubic scaling of exact GP inference (e.g., Casmopolitan^102^ dynamically constructs the GP within local trust regions). In contrast, our method approximates a GP via VSA encodings and performs gradient-based optimization directly in the embedding space.

**Fig. 5:**
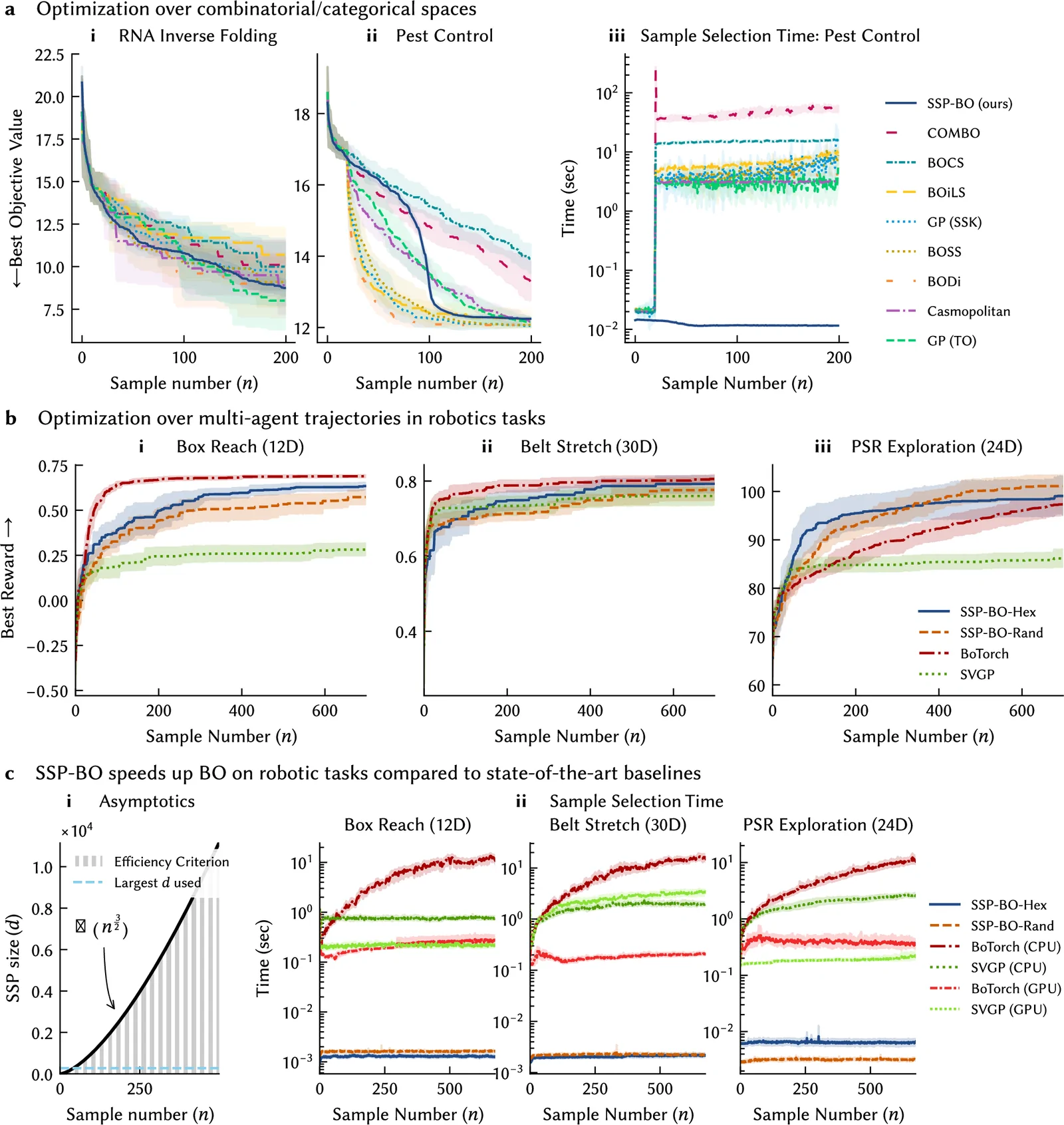
VSA allow representation and fast optimization over combinatorial spaces. **a** VSAs allow representation and optimization over combinatorial/categorical spaces. Plots **(i–ii)** show the best objective function value (lower is better for these two tasks); plot **(iii)** shows sample selection time on a log scale. In all figures, shaded regions are 95% bootstrap confidence intervals for 10 trials with sampling budgets of *N* = 200. SSP-BO again shows a substantial improvement in sample selection times. **b** SSP-BO comparable to state-of-the-art baselines on robotics tasks. **(i–iii)** show the performance of SSP-BO and baselines in terms of largest reward achieved as a function of the number of samples. In all plots, the lines show the mean of 10 runs (each run initialized with a different random seed); shaded regions indicate 95% confidence intervals. BoTorch leads on a simple Box Reach task **(i)**, SSP-BO attains comparable performance on the more advanced Belt Stretch task with deformables **(ii)**, and finally SPP-BO outperforms BoTorch and other baselines on the PSR planetary exploration task with four agents **(iii)**. **c** SSP-BO speeds up BO on robotic tasks. **(i)** The values of SSP dimensionality, *d*, and number of samples, *n*, for which SSP-BO is more efficient than exact GP-based BO in terms of time complexity asymptotics (shown by the shaded region). Exact GP-BO has an ${{{\mathcal{O}}}}({n}^{3})$ time complexity, while SSP-BO is constant in *n* but quadratic in *d*, i.e., ${{{\mathcal{O}}}}({d}^{2})$. Consequently, the instantaneous sample point selection for SSP-BO is an improvement over naive GP BO when $d < {n}^{\frac{3}{2}}$ (the efficiency criterion). The dashed blue line shows the maximum SSP dimensionality used in our experiments. For this value, the theoretical break-even point is  ≈ 70 samples. **(ii)** The time to compute each next sample for SSP-BO, BoTorch, and SVGP for the Box Reach, Belt Stretch, and PSR Exploration tasks on a logarithmic scale. Lines show the mean of 10 runs; shaded regions indicate 95% confidence intervals. In these experiments, SSP-BO offers significant reduction in compute time.

In Fig. 5a, we report the best objective value rather than regret, as the true optimal value is unknown for these tasks. In these tasks, lower objective values are better. Within a budget of *N* = 200, our approach achieves a best objective value within the 95% confidence intervals of the top-performing BO methods in ref. ^98^, demonstrating competitive performance while substantially reducing computation time. The total time spent by SSP-BO planning actions and updating its surrogate model is 205 to 3891 times faster than the total time for the other BO methods on the pest control task and 176 to 2763 times faster on the RNA inverse folding task. While SSP-BO’s sample efficiency is worse than some methods on the pest control task, it still achieves a good objective value in a fraction of the time–even when function evaluation time is included. In particular, the total time SSP-BO takes to sample and evaluate 200 samples is at least 30 times less than the time the best-performing baseline (SSK) takes to sample and evaluate 100 samples.

The time reduction achieved by SSP-BO stems from three key factors: (1) efficient feature similarity computation compared to expensive kernels (e.g., SSK), (2) fast optimization in a smooth continuous space rather than slow genetic algorithms or hill climbing in discrete combinatorial spaces, and (3) avoiding the cubic scaling of exact GP inference through VSA kernel approximations.

To further validate our method, we apply it to a graph optimization task, NAS-Bench-101^103^, a benchmark for Neural Architecture Search (NAS). The goal is to find the network architecture the achieves highest accuracy on CIFAR-10. This requires optimizing over a structured, discrete search space where architectures are represented as directed acyclic graphs with varying layer types. Examples of graphs in the action space are given in Fig. 2.

During optimization, we query the validation accuracy of selected architectures and report here the best validation and test accuracies found, as is standard. The results are given in Table 1. The accuracies obtained by SSP-BO are within the standard deviation of several existing BO NAS methods (e.g., NASBOT^104^, NAO^62^, BANANAS^105^, and BONAS^106^). While our approach performs worse than state-of-the-art NAS methods such as AG-Net^107^, a generative approach to NAS, and DiNAS^108^, a graph diffusion algorithm, it is competitive while maintaining efficiency and suitable to neuromorphic implementation. Most importantly, these results demonstrate that our method is capable of optimization over structured spaces like neural architecture graphs.

**Table 1:** Reported mean validation and testing accuracies, along with standard derivations, obtained on NAS-Bench-101 over 10 trials for different algorithms

Algorithm	Val. Acc (%)	Test Acc (%)	# Samples
Optimum	95.06	94.32	
NASBOT^104^	–	93.73	150
NAO^62^	94.66 ± 0.14	93.49 ± 0.59	192
BANANAS^105^	94.73 ± 0.17	94.09 ± 0.19	192
BONAS^106^	–	94.07 ± 0.24	200
AG-Net^107^	**94.90** ± **0.22**	**94.18** ± **0.10**	192
DiNAS^108^	**94.98** ± **0.17**	**94.27** ± **0.20**	150
SSP-BO (ours)	94.44 ± 0.20	93.88 ± 0.21	200

NASBOT results are averaged over 200 trials and BONAS results are averaged over 50 trials instead of 10, per the cited papers.

Having demonstrated that Hex-SSPs are a viable and efficient feature representation for BO and that VSAs can support BO over structured discrete spaces, we now move onto optimization in structured continuous spaces using hybrid continuous-symbolic representations. Specifically, we seek to optimize the trajectories of multiple robots, with the encoding in Equation (10), in three tasks relevant to robotics and machine learning. The first task, Box Reach, is a pick-and-place task. Two manipulators must learn to reach into a vessel. Pick-and-place tasks have multiple applications, from warehouse automation to laboratory sample handling. In the next scenario, Belt Stretch, two mobile manipulators are tasked with replacing a belt on a belt-drive mechanism. Manipulating non-rigid objects is a challenging task and has applications in household maintenance, manufacturing and repair of machinery, and elder care, amongst others. The third task involves planning trajectories through the Permanently Shadowed Region (PSR) of a crater that maximize the observed counts from a simulated Neutron Spectrometer. Lunar prospecting is just one example of a coordinated, multi-agent, active learning task, but applications generalize to other information-gathering tasks such as resource mapping, search and rescue, and system identification.

We compare SSP-BO against a state-of-the-art baseline, BoTorch, and BO using Sparse Variational Gaussian Processes (SVGPs). SVGP has constant time and memory complexity. The hyperparameters of SVGP are set to match the memory usage of SSP-BO.

In these test cases, we do not know what the highest possible reward is and so we report the best scoring reward achieved by sample number *n* rather than regret. In these tasks, higher rewards are better. Figure 5b shows the evolution of the best discovered reward by SSP-BO and the baseline algorithms for 10 trials. In two cases we are achieving statistically equivalent performance to the baseline BO algorithm and in all scenarios SSP-BO has equivalent or superior terminal reward to SVGP. However, SSP-BO is substantially faster than BoTorch and even SVGP (see Fig. 5c).

Sample selection in SSP-BO is made possible on neuromorphic hardware by optimizing directly within the high-dimensional embedding space. This avoids operating in the complex, discontinuous action space and allows gradient-based optimization to be carried out through the dynamics of a recurrent spiking neural network. We implemented this approach on SpiNNaker and Loihi 2, demonstrating that efficient sample selection is achievable on both platforms. Implementation details are provided in section “Neuromorphic Implementation for SSP-BO”.

Results on the global test optimization functions are shown in Fig. 4(b), which illustrates regret reduction on both platforms. Note that the results for Loihi 2 are better than those for SpiNNaker. This is because we were able to run a larger model on Loihi 2 (880 neurons versus 385) and use a more expressive neuron model (see Supplementary Note 2, “Hardware”). Energy consumption per optimization step is summarized in Table 2. The improvement factor is 30.4-62.2 for Loihi 2 and 188.1 for SpiNNaker, demonstrating the substantial energy-efficiency gains of the neuromorphic implementations compared to the CPU baseline.

**Table 2:** The energy use per optimization step of SSP-BO when deployed on neuromorphic hardware

Hardware	Number of neurons	Energy (J/sample)
CPU	—	(234 ± 64) × 10^−3^
SpiNNaker	385	(1.244 ± 0.005) × 10^−3^
Loihi 2	440	(3.760 ± 1.30) × 10^−3^
Loihi 2	880	(7.451 ± 0.751) × 10^−3^

The CPU version implements the gradient-based algorithm on standard hardware. All models use SSP size *d *= 55. The model run on Loihi2 was sufficiently small that the in-built power profiler was unable to register its power consumption. Power results are from running a larger model and then scaling down consumption accordingly.

**Fig. 6:**
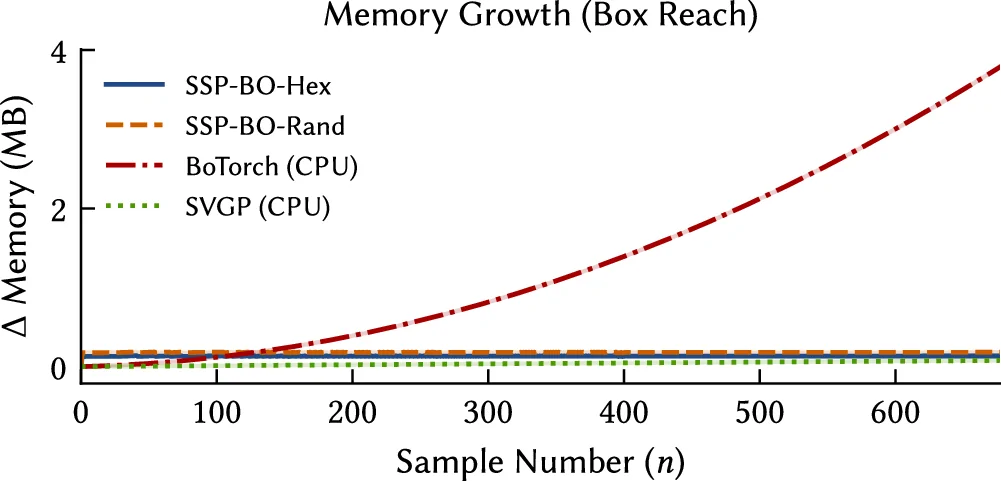
Memory complexity growth for SSP-BO, BoTorch, and SVGP. As with the standard GP implementations, BoTorch has a quadratic growth in memory consumption, while SSP-BO and SVGP remain constant. It takes ~106 samples on this 12 dimensional problem before BoTorch exceeds the memory complexity of SSP-BO. Data logged using the Heapy python memory profiler, data is from 10 trials, shaded regions represent 95% confidence intervals (not visible on graph, since memory utilization doesn’t have large fluctuations).

Figure 5c illustrates the growth of the wall-clock time for selecting the next sample location. For BoTorch, this grows with the number of samples, but for SSP-BO and SVGP this is effectively constant in all simulation scenarios (see also Fig. 4aiv). SSP-BO provides a pragmatic improvement over standard BO approaches, even on traditional computing hardware. Figure 5cii shows the total integrated complexity for a trial of *n* samples. The curve defines a threshold for the SSP size after which it is more efficient to use a GP implementation of BO. The time to compute the next sample point for SSP-BO is consistent with the predicted complexity from an asymptotic analysis–it remains constant regardless of the sample number. By adopting SSP-BO, we reduce the total time spent planning in our experiments by up to 205 × (see Table 3). Similarly, SSP-BO’s memory complexity is constant in *t*, in contrast with the empirical results from GP-BO (Supplementary Fig. 1) and the BoTorch (Fig. 6) algorithms. For SVGP, memory grows until the number of samples is greater than the maximum number of inducing points, at which point it becomes constant like SSP-BO. Overall, SSP-BO achieves efficiency gains by adopting VSA computing, without loss in performance.

**Table 3:** Total cumulative time spent planning sampling actions

	Total Time (sec ± s.e.)		Improvement
	Exact GP	SSP-BO (ours)	
Himmelblau	4.44 ± 1.52	**1.44** ± **0.08**	3.1 ×
Goldstein-Price	6.54 ± 0.99	**1.49** ± **0.06**	4.4 ×
Branin-Hoo	7.19 ± 1.58	**1.28** ± **0.12**	5.6 ×
RNA Inverse Folding	701.96 ± 2.65	**3.42** ± **0.04**	205.5 ×
Pest Control	527.17 ± 6.18	**2.99** ± **0.06**	176.3 ×
Box Reach	148.34 ± 9.20	**0.90** ± **0.02**	164.8 ×
Belt Stretch	128.87 ± 2.50	**1.45** ± **0.03**	88.9 ×
PSR Exploration	267.28 ± 18.96	**4.49** ± **0.28**	59.5 ×

The Exact GP baseline is GP (Matérn) for the standard target functions, GP (TO), the fastest baseline, for the combinatorial tasks, and BoTorch (GPU) for the robotics tasks. There is at least a 3 × improvement over baseline on toy problems, with as much as 200 × improvement over state-of-the-art for the more complicated tasks.

## Discussion

We present a BO algorithm, SSP-BO, which leverages VSA-based generalization of kernel embeddings and is the first BO method in which sample selection is demonstrated on neuromorphic hardware. The VSA embeddings enable us to retain the computational benefits of RFF-based GP approximations while extending BO to discrete, mixed, and structured data via hybrid symbolic-continuous representations.

SSP-BO delivers substantial computational gains, achieving constant-time and constant-memory sample selection with respect to the number of prior observations. We note that the embedding dimensionality *d* required to maintain a given error bound may grow with the number of observations for certain function classes. In many applications, including neuromorphic settings, *d* is fixed by hardware capacity, and one accepts a trade-off between representational fidelity and constant-time performance.

Empirically, we observe reductions of ~3 × to 200 × in total sample selection time, and at least a 30 × reduction in power consumption when translated to neuromorphic hardware. Memory consumption likewise favors SSP-BO, both empirically and asymptotically. These advantages grow with the number of observations, as standard GP costs scale with *n* while SSP-BO’s remain fixed.

A range of prior work has addressed compositional kernel construction for structured or discrete data. ^67^ proposed an algebra of tensor products and sums over kernels to construct kernels over discrete structures. ^68^ extended this to diffusion kernels, a generalized method of constructing kernels for data structures. These algebraic constructions underpin the automated identification of kernel functions as explored in the Automatic Statistician line of research^7,8,61^. These methods, while powerful, suffer from high computational cost due to *n*-wise comparisons over discrete domains.

More recently, BO in structured or combinatorial domains has used high-dimensional latent embeddings of discrete data. For example,^64^ and^100^ project combinatorial spaces into learned latent spaces using diffusion kernels, decoding optimal points via learned neural decoders.^109^ instead reparameterize acquisition functions over probability distributions on discrete spaces. These approaches allow standard BO machinery to operate over complex input domains, but often require learned encoder-decoder pairs, and repeated nested optimizations over discrete and continuous components.

Existing work on kernel approximation via RFFs demonstrates that additive kernels can be efficiently approximated by concatenating the feature maps for each component kernel^72^. However, composing kernels via multiplication requires tensor products of features, causing dimensionality to grow multiplicatively^10^. While low-rank tensor decompositions can alleviate this^110^, these methods require additional training.

In SSP-BO, we decode a VSA representation to obtain a data point, evaluate the objective function, and update the acquisition function with the re-encoded sample. Like prior latent-space BO methods,^64,109^ SSP-BO performs acquisition updates in a latent space. However, unlike these approaches, it avoids learned representations entirely.

This design has several advantages. First, it yields interpretable embeddings: each component of the representation corresponds to a defined algebraic structure, not an opaque neural transformation. Second, unlike learned encoders, VSAs have established theory regarding their representational capacity (see section “Representational Capacity and Scaling Properties”), offering stronger guarantees about generalization and expressivity. Moreover, many structured kernels–such as those used in graph and string domains^58,59^–can be reformulated using VSAs. This formulation provides significant flexibility while keeping resource demands bounded. Because SSP-BO operates entirely in the embedding space, it avoids costly pairwise kernel computations and eliminates the need for multi-stage optimization pipelines. This makes SSP-BO especially attractive for scaling BO to complex, symbolic, or hybrid domains under finite-resource constraints.

Our method extends the applicability of kernel embeddings in several key ways, by providing an algebraic framework that realizes it within fixed computational resources, complementing standard kernel theory. First, we offer a compositional, dimensionality-preserving language for kernel construction, compatible with resource-constrained systems such as neuromorphic platforms. Second, the VSA framework naturally represents mixed discrete-continuous data in a unified space. While other methods require separate passes over different modalities and compute *n*-wise comparisons, our compositional algebra enables single-loop optimization across structured and hybrid domains. Decomposition only occurs during decoding, after a sample is chosen and not during optimization. We exploit this to apply a single optimization strategy across global function optimization (continuous spaces), combinatorial/graph optimization (discrete spaces), and robotics problems (mixed discrete-continuous spaces).

Finally, the nature of our algebra affords a degree of interpretability that is absent in learned encoder-decoder approaches [e.g., ^61,63^]. Because the feature representations are explicitly constructed and algebraically manipulable, we can build, modify, or update them directly–composing new embeddings from atomic elements, shifting substructures, or rebinding values in place. This expressivity has been used in prior work on localization and mapping in spiking neural networks^111,112^.

This is the first work to implement part of a BO loop–sample selection–on neuromorphic hardware. Previous efforts like LavaBO^113,114^ implement BO-compatible GPs, but only on CPUs, with future neuromorphic deployment intended. Likewise, earlier work on SSP-BO presented Neuromorphic-compatible optimization but was tested on a CPU^115^. In contrast, here we test our method on both SpiNNaker and Loihi 2.

Several efforts have explored accelerating GP inference using other specialized hardware. In particular, Cholesky decomposition and matrix-vector operations have been implemented on FPGA^116,117^ and ASIC^118^ platforms. However, these works focus on low-level GP inference rather than addressing the overall scaling limitations of GP-based BO or its application to high-dimensional action spaces.

Reducing BO’s compute and memory demands introduces tradeoffs in approximation accuracy. However, as discussed in section “Representational Capacity and Scaling Properties”, the representational power of VSA bundles and Fourier embeddings is well-studied. For example, decoding error scales linearly with the number of bundled variables, indicating that while embedding size must grow with data complexity, the scaling remains tractable.

We note here that the HRR operations could, in principle, be combined with traditional RFFs to approximate compositional kernels. Future work could explore this. Moreover, the action spaces explored here are only a subset of what VSAs can represent. VSAs have been used to represent hash tables^119^, binary trees^85^, knowledge graphs^87^, words^120^, grammars^121^, and even images^122^. These results justify further exploration of SSP-BO in structured optimization.

Two major directions for future research are handling concept drift and enabling parameter adaptation within the BLR model. Concept drift could be addressed by introducing decay mechanisms into the BLR updates (Equation ??) to emphasize recent samples. Another promising approach is context binding, allowing for multi-context models that switch or blend based on task inference.

We also conjecture that adaptive parameters–particularly the length scale *ℓ* in Equation (7)–could improve posterior refinement during later stages of learning. While SSP-BO performs strongly across the test cases reported here (and the BoTorch baseline already leverages adaptive parameters), performance may plateau as the optimization progresses. Given the connection between HexSSPs and mammalian grid cells, it is noteworthy that biological systems exhibit spatial adaptation: grid cells rescale with environmental change^123^, and place cells reorganize during learning^124^. Adaptive spatial representations may be crucial for long-term, flexible learning.

More generally, it would be worth exploring how the behavior and neural circuits implied by our model map onto models of learning and exploration that are tightly coupled to hippocampal anatomy [ref. ^125^]. More broadly, because SSP-BO operates entirely within the VSA algebra, it can serve as a drop-in component for exploration under uncertainty in existing VSA-based cognitive architectures such as the models of decision making, memory, spatial mapping, and other domains that already use these representations.

A final direction involves incorporating non-stationarity. In recursive SSPs^90^, embedding parameters such as length scale or phase matrices vary across space. This offers increased representational capacity and spatially dependent resolution. While conceptually promising, such embeddings remain underexplored and introduce additional complexity to encoding and decoding. Developing principled learning algorithms or heuristics for constructing recursive SSPs tailored to BO is an open challenge.

Bounded, or at least slowly-growing, resource requirements are vital to continually learning systems. ^126^ identified a number of biological mechanisms to support lifelong learning, including context-dependent perception and gating. Whether the algebraic nature of VSAs can support, or perhaps is a high-level description of, such mechanisms is an open point of investigation.

While primarily focused on the pragmatic benefits of SSP-BO in the context of robotics and reinforcement learning, this work closes an important explanatory gap in cognitive science. Specifically, while prior work proposed that compositional kernels are important for understanding how humans can learn with few samples, a neural implementation of these kernels was lacking. In this work, we have shown that compositional methods from cognitive science can provide compositional kernels without exploding the dimensionality of the representation. Because algebraic statements in VSAs can be converted into neural networks, and because we have constructed complex VSA representations from neural models of continuous and discrete spaces, we propose that VSAs close the gap between rule-like, compositional methods and neural implementations. A more detailed discussion of this point is in Supplementary Note 7, “Neuroscience and Cognition.

## Methods

This section provides additional derivations for VSA-based kernel approximation, along with details on baseline methods and experimental setup. We first review Hexagonal Spatial Semantic Pointers [HexSSP; ^36^], a structured projection of continuous data inspired by grid-cell neural representations^127^. We then elaborate on how VSA encodings support approximate compositional kernel construction. Decoding methods for extracting valid actions from optimization outputs are described, followed by a discussion of the representational capacity of VSA encodings. We review the baseline BO methods, highlighting key differences between RFF-based BO and our SSP-BO approach. We also detail the objective functions from the original robotics tasks. Lastly, we describe the recurrent spiking neural network used for sample selection in SSP-BO on neuromorphic hardware.

### Hexagonal SSPs

Hexagonal SSPs (Hex-SSPs) are a high-dimensional embedding of continuous-valued data in the HRR framework^36^. The construction of these embeddings can be thought of as an additive combination of hexagonally-tiled kernels, each defined by grids of different scales and orientations. Algorithm 2 shows the steps for constructing Hex-SSPs.

#### Algorithm 2

Hexagonal SSP Phase Matrix Generator. *m* is the dimensionality of the data being encoded; ${{{\mathcal{H}}}}\subseteq {\mathbb{R}}$ are scaling values; ${{{\mathcal{R}}}}$ are *m*-dimensional rotation matrices, and **V** are structured phase matrices (see text below for details).

1:  **procedure** HEX-SSP(*m*, ${{{\mathcal{H}}}}$, ${{{\mathcal{R}}}}$)

2:   ${{{\bf{V}}}}\leftarrow {\left[{{{{\bf{v}}}}}_{1}| \cdots | {{{{\bf{v}}}}}_{m+1}\right]}^{T}$ ⊳ Vertices of a regular *m*-simplex

3:   $${{{\boldsymbol{A}}}}\leftarrow {{{\rm{stack}}}}\left(\left\{{h}_{i}{{{\bf{V}}}}{{{{\bf{R}}}}}_{i}\,| \,{h}_{i}\in {{{\mathcal{H}}}},{{{{\bf{R}}}}}_{i}\in {{{\mathcal{R}}}},i=1,\ldots,G\right\}\right)$$

4:   **return** ***A***

5 : **end Procedure**

Hexagonal SSP phase matrices are constructed from a regular *m*-simplex in an *m*-dimensional space. The simplex is determined by minimizing the relation $\mathop{\sum }_{i=1}^{m+1}\mathop{\sum }_{j=1,i\ne j}^{m+1}{{{{\bf{v}}}}}_{i}\cdot {{{{\bf{v}}}}}_{j}$, where **v**_*i*_, **v**_*j*_ are unit vectors that make up the simplex. These vectors form the rows of an initial (*m* + 1) × *m* phase matrix, **V**. We can specify the kernel function, $k({{{\bf{x}}}},{{{{\bf{x}}}}}^{{\prime} })$ that is approximated by the dot product between two SSPs, *ϕ*(**x**) and $\phi ({{{{\bf{x}}}}}^{{\prime} })$, by specifying the phase matrix of the encoding.

**Fig. 7:**
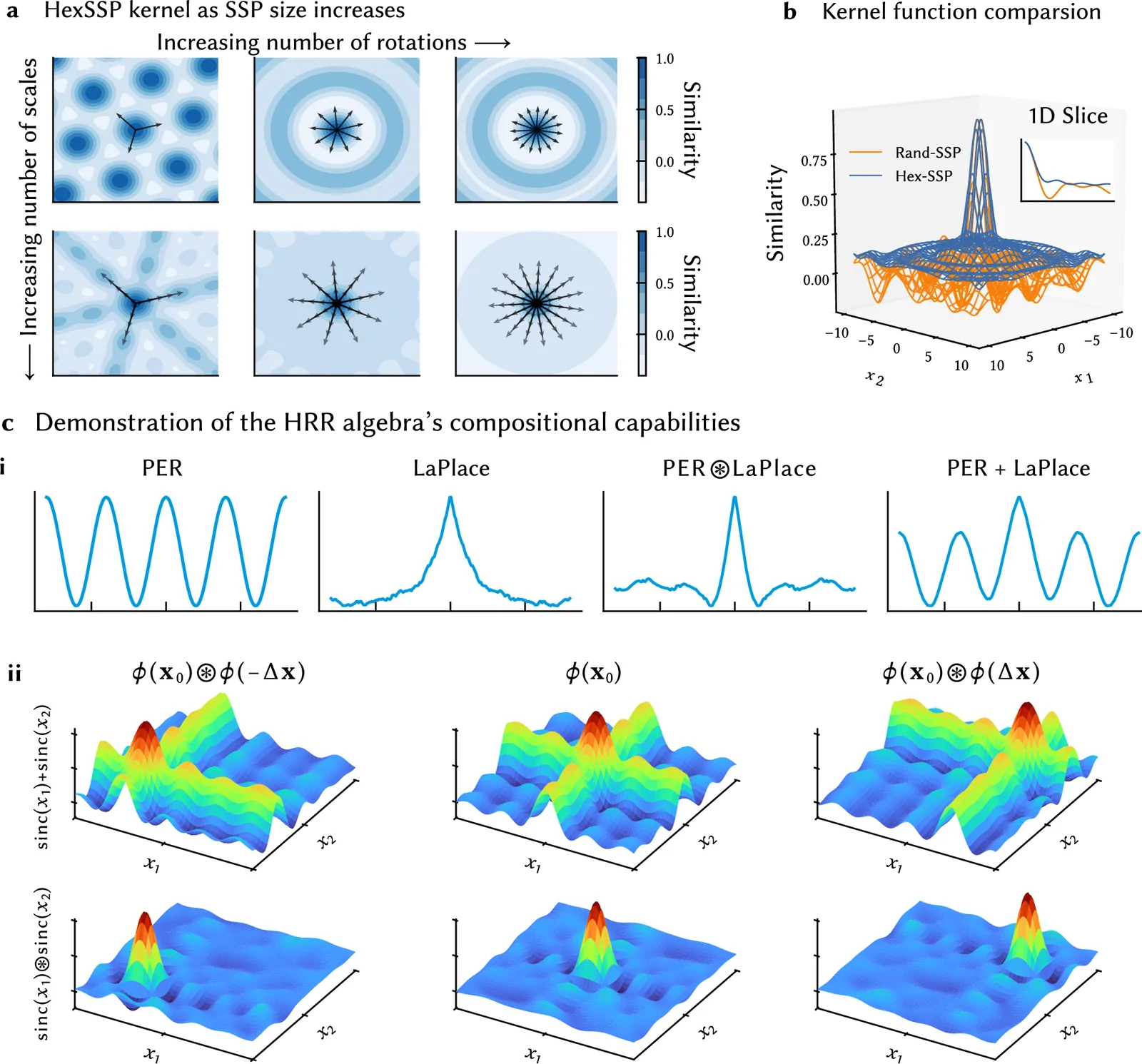
SSP kernel functions and kernel composition. **a** Kernel function induced by Hex-SSPs representing points in a 2D space as a heat map. The initial 3-component simplex (top left) produces hexagonal interference patterns like those found in grid cell neurons. Adding rotated simplices of the same scale (top right) produces a surface with a central peak surrounded by rings. Many differently-scaled simplices with the same orientation (bottom left) result in a starburst pattern. Adding many rotated and scaled simplices (bottom right) reduces the magnitude of the surrounding rings and increases smoothness and robustness to noise. **b** Rand-SSPs result in a sinc kernel function, while Hex-SSPs produce a sum of sinc functions (Equation (26)), making local maxima and minima are more shallow. **c** Demonstration of the HRR algebra’s compositional capabilities. **(i)** Shows the addition and multiplication of one dimensional kernels using periodic and LaPlace kernels encoded as SSPs. **(ii)** Shows that data encoded using additive and multiplicative kernel embeddings can be updated post-embedding. The central column shows the similarity between points in the domain and the original encoded point, **x**_0_. The left column shows the origin point shifted by  − *Δ***x**, a shift that is executed after encoding into the feature space using the binding operation of the HRR. The right column shows the same shift in the similarity map but for *Δ***x**. A benefit of using VSAs is the ability to manipulate and update values after they have been projected into the high-dimensional space.

As a concrete example, consider the phase matrix for an SSP encoding of two-dimensional variables. The simplest simplex is the 2-simplex, formed from an equilateral triangle with three vertex vectors, **v**_*j*_, that are 120° apart. With these vectors defining the initial phase matrix, **V**, the resulting kernel function will have a hexagonally tiled pattern (the leftmost plot of Fig. 7a). This is due to the interference pattern of plane waves defined by the vectors **v**_*j*_. As the dimensionality of a Hexagonal SSP is increased (moving rightward and downward in Fig. 7a) more simplices are added, resulting in an interference pattern that enables Hex-SSPs to approximate a smooth kernel, and do so better than Rand-SSPs (Fig. 7b), which approximate a sinc function. While the sinc kernel is a positive definite function, it is not a probability density as it takes on negative values. However, it is still usable in kernel machines.

### Kernel approximations with VSAs

#### Kernels over discrete encodings

Discrete symbols (e.g., concepts, categories, slots) are represented in a high-dimensional vector space (see Equation (1)). The dot product between symbols represented in this fashion induces a kernel. This is a smoothly varying function with a relatively wide bandwidth, potentially inducing high similarity between randomly selected symbols. However, because we are randomly selecting points from a high-dimensional hypersphere, the likelihood of two vectors being similar is relatively low. The distribution of the dot product, $\kappa=\phi (s)\cdot \phi ({s}^{{\prime} })$, between two randomly selected vectors, *ϕ*(*s*), $\phi ({s}^{{\prime} })$, representing the symbols $s,{s}^{{\prime} }\in {{{\mathcal{S}}}}$ is known to be^128,129^:

21 $$p(K=\kappa )=\frac{{(1-{\kappa }^{2})}^{\frac{d-1}{2}-1}}{{{{\rm{Beta}}}}\left(\frac{1}{2},\frac{d-1}{2}\right)}.$$

The expected value of the dot product between any two such vectors is zero, with a variance inversely proportional to the dimensionality of the representation, ${{{\rm{var}}}}(K)=\frac{1}{d}$. This means that noise induced by similarity between two different symbols can be controlled by increasing the representational dimension. The implication of this is that we can understand the kernel over discrete symbols to be:

22 $$k(s,{s}^{{\prime} })=\left\{\begin{array}{ll}1&s={s}^{{\prime} }\\ \kappa \sim p(K)&\qquad{{{\rm{otherwise}}}}\\ \end{array}\right.$$

Equation (22) is a useful formulation when analyzing the effect of cross-talk when taking the dot product between vector representations that are bundles of other vectors. By binding data vectors with symbol-vectors that specify the slot the data fills, one can make cross-talk terms arbitrarily small, for sufficiently high-dimensional vectors.

#### Kernels over continuous encodings

The dot product between SSPs is a kernel function, *k*(**x**, **y**) = *k*(**x** − **y**) = *ϕ*(**x**) ⋅ *ϕ*(**y**). In general, the kernel’s shape is dependent on the sampling distribution of the phase matrix components–in particular, it resembles the function whose power spectral density is the sampling distribution^9,27^. Suppose that the columns of the phase matrix are independent and identically distributed, sampled from a distribution *P* with probability density function *p*(**a**), then the expected kernel is

23 $${{\mathbb{E}}}_{A \sim P}\left[\phi ({{{\bf{x}}}})\cdot \phi ({{{\bf{x}}}}+\Delta {{{\bf{x}}}})\right]={{\mathbb{E}}}_{A \sim P}\left[\frac{1}{d}\overline{{e}^{iA{{{\bf{x}}}}/\ell }}\cdot {e}^{iA({{{\bf{x}}}}+\Delta {{{\bf{x}}}})/\ell }\right],$$

24 $$={{\mathbb{E}}}_{A \sim P}\left[\frac{1}{d}\mathop{\sum }_{j=1}^{d}{e}^{i{{{{\bf{a}}}}}_{j}^{T}\Delta {{{\bf{x}}}}/\ell }\right],$$

25 $$=\int_{{{\mathbb{R}}}^{n}}{e}^{i{{{{\bf{a}}}}}^{T}\Delta {{{\bf{x}}}}/\ell }p({{{\bf{a}}}})d{{{\bf{a}}}},$$

obtained using Parseval’s theorem and the law of the unconscious statistician.

#### HexSSP kernel

The complete phase matrix ***A*** used to construct Hex-SSPs is composed of multiple rotations and scalings of **V**. **V** is constructed in 2D by drawing vectors from  ~ Uniform[−*r*, *r*]^2^, and from each vector computing the two additional vectors needed to form a simplex. This changes the kernel function. For ease of illustration we consider continuous variables ${{{\bf{x}}}},{{{{\bf{x}}}}}^{{\prime} }\in {{\mathbb{R}}}^{m=2}$ and let ${{{{\bf{x}}}}}^{{\prime} }={{{\bf{x}}}}+\Delta {{{\bf{x}}}}$. The expectation of the resulting kernel function is given by Equation (26) below. It is a sum of sinc functions of the difference between the query points, *Δ***x**, projected onto the standard simplex.

26 $$\begin{array}{ll}&{\mathbb{E}}[{k}_{{{{\rm{Hex}}}}-{{{\rm{SSP}}}}}({{{\bf{x}}}},{{{\bf{x}}}}+\Delta {{{\bf{x}}}})]\propto \left({{{\rm{sinc}}}}(\Delta {x}_{1}){{{\rm{sinc}}}}(\Delta {x}_{2})\right.\\ &+{{{\rm{sinc}}}}\left(-\frac{1}{2}\Delta {x}_{1}+\frac{\sqrt{3}}{2}\Delta {x}_{2}\right){{{\rm{sinc}}}}\left(-\frac{\sqrt{3}}{2}\Delta {x}_{1}-\frac{1}{2}\Delta {x}_{2}\right)\\ &\left.+{{{\rm{sinc}}}}\left(-\frac{1}{2}\Delta {x}_{1}-\frac{\sqrt{3}}{2}\Delta {x}_{2}\right){{{\rm{sinc}}}}\left(\frac{\sqrt{3}}{2}\Delta {x}_{1}-\frac{1}{2}\Delta {x}_{2}\right)\right)\end{array}$$

The rotations and scalings are introduced to explain the activity of neurons whose firing patterns have different grid sizes and orientations. However, an interesting pattern arises as more rotations and scales are added to the representation: the similarity function becomes smoother, with one centralized peak and more shallow local optima, as shown in Fig. 7a. Furthermore, Hex-SSPs are more robust to noise and can be more accurately encoded in spiking neural networks via grid cells^36^.

#### Data structures as approximate compositional kernels

Core to VSA representations is the ability to construct data structures out of bundling and binding operations, given a suite of symbols and vector embeddings of numerical data. In section “Vector Symbolic Algebra (VSA)”, we described how one can represent sets, graphs, and trajectories. VSAs have also been used to represent other data structures in past work, including binary trees^130^, stacks^131^, n-gram statistics^32^, images^122,132^, sentences^133^ and knowledge graphs^87^. A collection of permutation-based VSA encodings are surveyed by ref. ^85^, section IV, including stacks, finite state automata, and the directed graphs.

What is important about these representations is that their similarity, as computed by the vector dot product, captures similarity between data structures that is controlled by their structure. In other words, using vector representations as a base set, we can understand the HRR algebra as a language for composing kernels. In the style of ^8^, the algebra of HRRs permits the addition and multiplication of kernels, using the bundling and binding operators.

It has already established that the dot product between symbols (discrete or continuous) can be interpreted as kernel functions between the encoded data^12,27–29^. In fact, in this framework, there is no meaningful distinction between discrete, or continuous data. By building up data structures out of these elements, using the circular convolution algebra that Plate proposed, we will show that dot product similarity between data structures induces kernel functions for the data structures as well. This complements and expands on past work connecting VSAs to kernel approximation ^28^ and work on compositionality of functions embedded in reproducing kernel Hilbert spaces, using the FHRR algebra ^29^.

We appeal to the method of Random Fourier Features, originated by Rahimi and Recht^9^, to confirm that we can shape the kernels to our liking, as demonstrated by refs. ^29^, ^134^, and ^36^. What we show next is that binding and bundling can be used to construct kernels from elementary components. However, we note that, by virtue of the finite representations in neural networks, all kernels will necessarily be bandlimited kernels.

Given that the dot product between base vectors induces a kernel, we can use binding to multiply kernels. We show the connection between bundling and kernel multiplication through operation in the Fourier domain:

27 $$\begin{array}{ll}&{{{\mathcal{F}}}}\left\{{\phi }_{{{{\rm{X}}}}}({{{\rm{x}}}}) \circledast {\phi }_{{{{\rm{Y}}}}}({{{\rm{y}}}})\cdot \ {\phi }_{{{{\rm{X}}}}}({{{{\rm{x}}}}}^{{\prime} }) \circledast {\phi }_{{{{\rm{Y}}}}}({{{{\rm{y}}}}}^{{\prime} })\right\}\\ &={{{{\rm{e}}}}}^{{{{\rm{i}}}}{A}_{X}{{{\bf{x}}}}}\odot {{{{\rm{e}}}}}^{{{{\rm{i}}}}{A}_{Y}{{{\bf{y}}}}}{({{{{\rm{e}}}}}^{{{{\rm{i}}}}{A}_{X}{{{{\bf{x}}}}}^{{\prime} }}\odot {{{{\rm{e}}}}}^{{{{\rm{i}}}}{A}_{Y}{{{{\bf{y}}}}}^{{\prime} }})}^{{{\dagger}} }\end{array}$$

28 $$=D({{{{\rm{e}}}}}^{{{{\rm{i}}}}{A}_{X}{{{\bf{x}}}}}){{{{\rm{e}}}}}^{{{{\rm{i}}}}{A}_{Y}{{{\bf{y}}}}}{({{{{\rm{e}}}}}^{-{{{\rm{i}}}}{A}_{Y}{{{{\bf{y}}}}}^{{\prime} }})}^{{{\dagger}} }D({{{{\rm{e}}}}}^{-{{{\rm{i}}}}{A}_{X}{{{{\bf{x}}}}}^{{\prime} }})$$

29 $$\approx D({{{{\rm{e}}}}}^{{{{\rm{i}}}}{A}_{X}{{{\bf{x}}}}}){k}_{{{{\rm{Y}}}}}(y,{y}^{{\prime} })D({{{{\rm{e}}}}}^{-{{{\rm{i}}}}{A}_{X}{{{{\bf{x}}}}}^{{\prime} }})$$

30 $$={{{{\rm{e}}}}}^{{{{\rm{i}}}}{A}_{X}{{{\bf{x}}}}}{({{{{\rm{e}}}}}^{-{{{\rm{i}}}}{A}_{X}{{{\bf{x}}}}})}^{{{\dagger}} }\odot {k}_{{{{\rm{Y}}}}}(y,{y}^{{\prime} })$$

31 $$\approx {k}_{{{{\rm{X}}}}}({{{\bf{x}}}},{{{{\bf{x}}}}}^{{\prime} }){k}_{{{{\rm{Y}}}}}({{{\bf{y}}}},{{{{\bf{y}}}}}^{{\prime} })$$

where *D*( ⋅ ) signifies the diagonal matrix of its argument and † is the conjugate transpose. In equations (29) and (31) we use the approximation sign to indicate we are approximating a kernel function with a finite-dimensional dot product.

To construct additive kernels, we can use bundling of vector-encodings of data. This connection is trivial to demonstrate, as the dot product distributes over the vector sum:

32 $$\begin{array}{ll}&({\phi }_{{{{\rm{X}}}}}({{{\bf{x}}}})+{\phi }_{{{{\rm{Y}}}}}({{{\bf{y}}}}))\cdot ({\phi }_{{{{\rm{X}}}}}({{{{\bf{x}}}}}^{{\prime} })+{\phi }_{{{{\rm{Y}}}}}({{{{\bf{y}}}}}^{{\prime} }))\\ &={\phi }_{{{{\rm{X}}}}}({{{\bf{x}}}})\cdot {\phi }_{{{{\rm{X}}}}}({{{{\bf{x}}}}}^{{\prime} })+{\phi }_{{{{\rm{X}}}}}({{{\bf{x}}}})\cdot {\phi }_{{{{\rm{Y}}}}}({{{{\bf{y}}}}}^{{\prime} })\\ &+{\phi }_{{{{\rm{Y}}}}}({{{\bf{y}}}})\cdot {\phi }_{{{{\rm{X}}}}}({{{{\bf{x}}}}}^{{\prime} })+{\phi }_{{{{\rm{Y}}}}}({{{\bf{y}}}})\cdot {\phi }_{{{{\rm{Y}}}}}({{{{\bf{y}}}}}^{{\prime} })\\ \end{array}$$

33 $$\begin{array}{ll}&\approx {k}_{{{{\rm{X}}}}}({{{\bf{x}}}},{{{{\bf{x}}}}}^{{\prime} })+{\phi }_{{{{\rm{X}}}}}({{{\bf{x}}}})\cdot {\phi }_{{{{\rm{Y}}}}}({{{{\bf{y}}}}}^{{\prime} })\\ &+{\phi }_{{{{\rm{Y}}}}}({{{\bf{y}}}})\cdot {\phi }_{{{{\rm{X}}}}}({{{{\bf{x}}}}}^{{\prime} })+{k}_{{{{\rm{Y}}}}}({{{\bf{y}}}},{{{{\bf{y}}}}}^{{\prime} })\\ \end{array}$$

34 $$\approx {k}_{{{{\rm{X}}}}}({{{\bf{x}}}},{{{{\bf{x}}}}}^{{\prime} })+{k}_{{{{\rm{Y}}}}}({{{\bf{y}}}},{{{{\bf{y}}}}}^{{\prime} })+2\varepsilon$$

where 2*ε* is the cross-talk noise, $\phi ({{{\bf{x}}}})\cdot \phi ({{{{\bf{y}}}}}^{{\prime} })+\phi ({{{{\bf{x}}}}}^{{\prime} })\cdot \phi ({{{\bf{y}}}})$. Examples of multiplicative and additive kernels are given in Fig. 7ci in one dimension, and in two dimensions in Fig. 7cii (multiplicative in bottom row and additive in top row). There are pathological cases where the cross-talk noise is large, e.g., ${{{\bf{x}}}}={{{{\bf{x}}}}}^{{\prime} }={{{\bf{y}}}}={{{{\bf{y}}}}}^{{\prime} }\approx {{{\bf{0}}}}$. Cross-talk for additive kernels can be mitigated by using concatenation instead of bundling, as has been employed in RFF-based approaches [e.g.,^72^]. However, concatenation does not preserve dimensionality, and in the spatial domain, concatenation destroys the ability to apply binding techniques to vector representations.

Fortunately, in the HRR algebra, cross-talk noise can be controlled through the use of slot-filler representations. A bundle of the form *ϕ*_X_(**x**) + *ϕ*_Y_(**y**) may be re-written as *G*_X_ ⊛ *ϕ*_X_(**x**) + *G*_Y_ ⊛ *ϕ*_Y_(**y**) where *G*_X_ and *G*_Y_ are randomly generated discrete vectors that denote the role the data is playing in the sum. This ensures that variables **x** and **y** are projected onto different parts of the high-dimensional feature space. Consequently, the cross-talk noise is scaled by the similarity between the slot vectors, *G*_X_ and *G*_Y_, which should be very small. Bundling, after binding to a slot, lets us approximate additive kernels. Figure 7c illustrates the ability to compose kernels using the HRR algebra.

Since we know that positive definite kernels are closed under addition and multiplication^8,66^, and that our algebra can implement kernel multiplication and addition, we can represent compositional kernels, such as those produced by the kernel description grammar used by the Automatic Statistician^8^. These results also imply that, provided we have meaningful data projections for the individual elements, any data structure that is constructed from bundling and binding operations implicitly creates a bandlimited kernel function for the structured data.

### Feature decoding

The BO routine produces an optimal vector, θ^*^, in the feature space. However, the objective function requires sample points from its domain. In systems where VSA embeddings are used throughout (e.g., a robot whose motor system operates directly on VSA-encoded commands θ^*^ may be used directly. In most cases, including all tasks in this paper, we must decode the optimal point in the domain, ${{{{\bf{x}}}}}^{*}\in {{{\mathcal{X}}}}$, from θ^*^.

Decoding SSPs through optimization, as in ref. ^72^, is challenging because kernels induced by the dot product between Hex-SSPs have an infinite number of local maxima, increasing the complexity of decoding. To decode an SSP θ^*^, we first coarsely sample ${{{\mathcal{X}}}}$ and encode the samples as SSPs. Samples are spaced by 2/*l*_*i*_ along each axis, *i* ∈ {1, …, *m*}, where *l*_*i*_ is the length scale used in the SSP encoding of the *i*^*th*^ axis. The SSP with the highest similarity to θ^*^ is used as the initial point of a gradient-based optimization, whose objective is to find **x**_*s*_ such that ${\phi }_{{{{\mathcal{X}}}}}({{{{\bf{x}}}}}_{s})$ has maximal similarity with the optimal SSP, θ^*^. The corresponding point **x**_*s*_ is selected as the decoded point. Since there is a connection between the VSA representations we use and probability distributions [as we discuss in refs. ^30,95^], this decoding process is akin to sampling a maximum a posteriori prediction.

One could alternatively decode using a neural network, as done in ref. ^89^. The downsides of this approach are that the required training data grows with the input domain dimensionality, and the network must be retrained for different length scales. However, the training data is easy to generate and network training can be done prior to deployment of the optimization algorithm.

For the combinatorial and graph VSA embeddings (Equation (3)), we decode by unbinding each slot vector and then finding the category vectors that maximize similarity: $\arg {{\max }_{j}}({\theta }^{*}\oslash {\phi }_{{{{\mathcal{S}}}}}({s}_{i}))\cdot {\phi }_{{{{{\mathcal{V}}}}}_{i}}({v}_{i,j})$ for *i* = 1 to the total number of categories. Likewise, with trajectory embeddings, we can use unbinding to decode the proposed set of waypoints: $\arg {{\max }_{{{{\bf{x}}}}}}({\theta }^{*}\oslash {\phi }_{T}({t}_{i}))\cdot {\phi }_{{{{\mathcal{X}}}}}({{{\bf{x}}}})$.

### Representational capacity and scaling properties

One of the key advantages of SSP-BO is that its runtime and memory requirements remain constant with respect to the number of collected observations, *n*. Specifically, it avoids the ${{{\mathcal{O}}}}({n}^{3})$ time and ${{{\mathcal{O}}}}({n}^{2})$ memory costs of standard GP surrogate models, replacing them with ${{{\mathcal{O}}}}({d}^{2})$ and ${{{\mathcal{O}}}}({d}^{2})$, respectively, where *d* is the dimensionality of the vector embeddings. The ${{{\mathcal{O}}}}({d}^{2})$ time complexity arises from using the Sherman-Morrison formula to perform rank-one updates to *Σ*_*n*_ (Equation (15)). Since *d* is a fixed, user-selected parameter that can be chosen based on the available computational budget, and the theoretical bounds reviewed later, SSP-BO is well suited to deployment on neuromorphic hardware and other resource-constrained platforms.

There is a well-established body of theory on the representational capacity of VSAs that we can draw on to understand how the choice of *d* affects performance. In short, both theory and empirical results show that the required dimensionality scales efficiently: linearly with the number of bundled items and only logarithmically with dictionary size for discrete representations (to obtain constant decoding error); and linearly with the data dimensionality for continuous representations (to obtain constant kernel approximation error). This means that relatively modest values of *d* are sufficient for accurate decoding and function approximation in practice. Additionally, unlike learned latent embeddings–where capacity limits and scaling behavior are often opaque–VSA-based methods offer transparent, analyzable tradeoffs between dimensionality, accuracy, and generalization.

*Discrete Representations:* Discrete symbols are encoded according to Equation (1). For bundles of *K* discrete vectors drawn from a dictionary of size *m*, ^83^ gives a bound on the dimensionality *d* needed to decode with error probability *q*:

35 $$d\ge 3.16(K-0.25)\log \left(\frac{m}{{q}^{3}}\right).$$

This bound confirms efficient scaling: *d* grows linearly with bundle size and only logarithmically with vocabulary size. Theoretical predictions have been validated in practice^20,135^ and extended by ref. ^79^, who benchmarked multiple VSAs. FHRRs and sparse binary architectures consistently required the lowest dimensionalities to achieve high decoding accuracy–outperforming alternatives like Multiply-Add-Permute, Vector-derived Transform Binding, and Matrix Binding of Additive Terms. Note that while our embeddings live in the real domain, as in HRR, our approach to generating vectors is actually taken from FHRR (thus, unbinding is exact rather than approximate). As such, the representational scaling of FHRRs is most relevant.

*Continuous Representations:* The SSP embedding of continuous data is similar to that of RFF–in fact, SSPs can be obtained from a linear transform on RFFs with particular constraints. Thus, capacity results from the RFF literature are applicable to SSP-BO. For example, the error of approximating a kernel is less than *ϵ* with high probability when

36 $$d\ge {{{\mathcal{O}}}}\left(\frac{\log {\mbox{diam}}\,({{{\mathcal{X}}}})}{{\epsilon }^{2}}\right),$$

where $\,{\mbox{diam}}\,({{{\mathcal{X}}}})$ is the diameter of the compact space that the values of **x** occupy [^136^, Theorem 1].

When applying kernel ridge regression using RFFs, the required dimensionality for minimax-optimal learning rates is:

37 $$d={{{\mathcal{O}}}}\left(\sqrt{N}\log {d}_{\lambda }\right)$$

where *d*_*λ*_ denotes the effective degrees of freedom of the kernel and *N* is the number of observations^137,138^. In other words, the required dimensionality grows sublinearly with data set size. ^139^ further showed that RFFs of size ${{{\mathcal{O}}}}(\sqrt{N}\log N)$ can match the generalization bounds of exact kernel ridge regression. This further supports the practical efficiency of SSPs in learning tasks. Empirically, we evaluate the effect of *d* and data size *m* on the performance of SSP-BO in Supplementary Note 4.3, “Dimensionality Experiments”.

Since all VSA structures ultimately represent bundles of bound items, the representational and regression requirements interact. A safe and effective practice is to select *d* according to the maximum of the bounds given in Equation (35) and Equation (37). That said, we emphasize that the regression bounds are derived under conservative assumptions – namely, uniform function approximation over entire input domains. In practice, SSP-BO often targets local or structured objectives, which allows for significantly smaller *d* values than theory might suggest.

For example, in our own experiments, values of *d* as low as 25 yielded strong performance across a range of challenging tasks. This confirms that SSP-BO remains computationally efficient and practically effective even in constrained settings.

### Neuromorphic implementation for SSP-BO

We implemented the sample selection step of the SSP-BO algorithm on neuromorphic hardware platforms. Sample selection in SSP-BO is enabled on neuromorphic hardware by formulating the optimization directly in the smooth, high-dimensional embedding space, rather than in the typically non-smooth action space. This allows a spiking neural network to perform gradient ascent on the acquisition function by evolving its internal neural state over time. Figure 1b illustrates the recurrent network architecture used for this process: a population of spiking neurons encodes the embedding *ϕ* nonlinearly, with linear decoding used to read out the current estimate of *ϕ*. Recurrent connections within the population are trained to approximate the gradient ascent of the acquisition function, following the principles of the Neural Engineering Framework (NEF;^140^). After 2.5 seconds of simulation time, the optimized point ϕ^*^ is decoded from the spiking population and used to update the surrogate model.

During each simulation window, the network evolves smoothly in the neural state space. When decoded back into the original low-dimensional action space, however, the resulting trajectory can exhibit jumps and a mixture of local and global search.

For the experiments reported on neuromorphic hardware, we use an SSP size of *d* = 55, which is lower than the *d* = 97 used in Fig. 4a. The SpiNNaker implementation uses 385 leaky integrate-and-fire (LIF) neurons, while the Loihi 2 implementation used 440 ReLU-like neurons, enabled by exploiting graded spike functionality.

### Baseline implementations for BO

As a baseline for our standard test function experiments we use the algorithm of ^50^, implemented using the scikit-learn Gaussian process package^141^, which we denote as GP (Matérn) or GP (sinc), depending on the choice of kernel. We also compare to approximate GP using RFFs, adapted from ^142^, which we denote as RFF-BO (see Supplementary Algorithm 3). This method uses the same acquisition function and BLR updates as SSP-BO (Equation (18) and Equation (16)), but differs in three ways, with the last two being the most significant.

- **Linear transform:** The RFFs are given by $\frac{2{\sigma }^{2}}{d}\,{\mbox{cos}}\,(A{{{\bf{x}}}})$, where *σ* is fit using the initial samples. This could be obtained from an SSP constructed using *A* via $\frac{2{\sigma }^{2}}{d}\,{\mbox{Re}}\,{{{\mathcal{F}}}}\{\phi ({{{\bf{x}}}})\}$–in other words, it differs from an SSP by a linear transform and an additional scaling term.
- **Phase matrix generation:** The phase matrix elements, *A*, in the RFFs are sampled from ${{{\mathcal{N}}}}(0,\frac{1}{\ell })$, where *ℓ* is optimized using the scikit-learn GP regressor with an RBF kernel over the initial samples. While in SSP-BO-Rand the elements are sampled from a uniform distribution and in SSP-BO-Hex they are generated using Algorithm 2.
- **Optimization domain:** In RFF-BO, the acquisition function is optimized over the data domain, ${{\max }_{{{{\bf{x}}}}\in {{{{\mathcal{R}}}}}^{n}}}\ {\psi }_{n}({{{\bf{x}}}})$. In contrast, SSP-BO optimizes in the embedding space over a continuous, compact domain and then decodes (Equation (19)).

An additional distinction is that RFF embeddings are not defined for the combinatorial, graph, or trajectory tasks. For the combinatorial tasks, we use the GP BO methods tested in ref. ^98^. The algorithms use various kernels, acquisition functions, and optimization methods that operate over combinatorial spaces. The options for each method included in Fig. 5a are given in Supplementary Table II.

For the robotics experiments, we use a state-of-the-art Bayesian optimization library called BoTorch^143^. BoTorch offers a variety of options for Gaussian processes (built on top of GPyTorch^144^) and their kernels. Additionally, it allows for kernel hyperparameters to be fit automatically using marginal likelihood, as described in Section V-A in ref. ^145^. Many recent BO methods are built on top of BoTorch and use the standard library as a baseline ^100,146–148^.

One of the most widely used families of kernels is the Matérn kernel family. Within this kernel family, the Radial Basis Function (RBF) kernel (also known as the Squared Exponential kernel) has been shown to perform well on a range of applications in robot locomotion and manipulation^149–154^. Hence, we use the RBF kernel in our experiments. We use UCB as the acquisition function for BO, since it is one of the most widely used options with both good practical performance and theoretical guarantees for regret bounds^94^. We compare against both exact GP inference–using BoTorch to compute Gaussian posteriors – and SVGP, a sparse variational GP model^155^ with a fixed number of inducing points, initialized following ^156^. The number of inducing points is chosen to match the memory usage of SSP-BO.

### Experimental setup and environments

We present three types of tasks, all of which are illustrated in Fig. 1c. The first task suite consists of a set of test functions commonly used for evaluating global optimization algorithms. The second evaluation suite consists of black-box optimization functions defined over multivariate discrete spaces (e.g., combinatorial variables, graphs). Functions from these first two sets have all been used as benchmarks in prior work. The third type of task contains robots and autonomous exploration tasks. Robot simulations were built using the PyBullet physics simulator^157^. All robotics tasks have a total path distance penalty term, *p*(**x**), to encourage efficiency.

#### Dual-arm robot manipulation

In the first robot experiment, Box Reach, there are two robot arms that need to reach targets in a box (see Fig. 1c). The two arms have different sizes. The smaller arm is able to reach into smaller boxes, but not larger boxes. The other arm is larger and can reach into boxes of all heights, but moves slower. The reward function is based on the distances to the target from the closest arm at each time step *t* (and the pentaly term *p*(**x**)):

38 $$\mathop{\sum }_{t=0}^{T}\exp \left\{-\min {\left({{\mbox{dist}}}_{t}({\mbox{goal}},{{\mbox{arm}}}_{1}),{{\mbox{dist}}}_{t}({\mbox{goal}},{{\mbox{arm}}}_{2})\right)}^{2}/2\right\}-p({{{\bf{x}}}}).$$

The control policy is a selection of two 3D waypoints, a mid-trajectory waypoint and an end-trajectory waypoint, for the end effector of each arm, which are then executed by an onboard operational space controller.

In the second robot experiment, Belt Stretch, the robots need to stretch a rubber belt onto a belt-drive mechanism (see the middle part of Fig. 1c). The robots need to learn to coordinate both arms while placing the rubber belt on the mechanism. The reward for the task is based on the distances between the current and desired belt pose. In our experiments, we express the desired belt pose by defining two target locations on each wheel of the drive mechanism and checking the distance from the four equally spaced locations (vertices) of the belt to these target locations on the wheels: $\mathop{\sum }_{t=0}^{T}\exp \left\{-\mathop{\sum }_{k=1}^{K=4}{{{\rm{dist}}}}_{t}\left.{\left({{\rm{belt}}}\_{{{\rm{vertex}}}}_{k},{{{\rm{target}}}}_{k}\right)}^{2}/2\right)\right\}-p({{\bf{x}}})$. The search space is five waypoints per arm, executed by both arms simultaneously, for a total of ten 3D waypoints.

#### Multi-robot exploration of permanently shadowed regions

In our third robot environment, we have a swarm of *n* robots collaboratively exploring a Permanently Shadowed Region (PSR) in order to find a maximum density of subsurface water. Here the task is executed in two stages. In stage one, the swarm of robots explore the interior of the PSR to find the maximum estimated subsurface water density. They do this exploration under the constraints of solar power, and need to exit the PSR to recharge and share map data. In the second stage the PSR is entered by a robot that carries a drill, which goes to the location of maximum estimated subsurface water density, and collects a soil sample before exiting the PSR. The first stage of activity acts as the Bayesian optimization, learning a function before executing on the learned knowledge.

The reward function during the exploration stage is the maximum of the estimated water density observed on each robot path. The control action is a set of waypoint trajectories assigned to the robots in the swarm. We assume that the robots will reverse their trajectories to return to the crater rim to recharge in sunlight.

Observations of subsurface water were modeled on an approximation of a neutron spectrometer. Neutron spectrometer data can be modeled as Poisson-distributed counts driven by the abundance of water in the subsurface under the instrument. In this experiment we are using high count values as a proxy for high water abundance.

We created a water map using the method described in (author?) [^158^, Ch. 4]. We constructed a Voronoi map from 10 seed points uniformly sampled from the crater interior. Each seed point was assigned values sampled uniformly in the range [20, 140], mimicking values observed in the Mojave desert during NASA’s Mojave Volatiles Prospector project. To prevent high-value locations from being generated outside of the PSR, a ring of low-value counts were added to the permimeter of the simulated PSR. We then convolved the Voronoi water map with a Gaussian blur of 7 pixels, to account for the blurring process of the field of view of the instrument. The above process creates a map from location to neutron counts, *c*(**x**), independent of terrain geometry. Observed counts are then defined as follows:

39 $${c}_{{{{\rm{obs}}}}}({{{\bf{x}}}})=\max \left\{0,c({{{\bf{x}}}})\cdot ({{{{\rm{height}}}}}_{\max }-{{{\rm{height}}}}({{{\bf{x}}}})/{{{{\rm{height}}}}}_{\max })\right\}$$

To account for the instability of water abundance outside of a permanently shadowed region, the count map is scaled down to zero when the elevation of the terrain exceeds the maximum height of the crater.

## Supplementary information

Supplementary Information

Transparent Peer Review file

## Data Availability

The performance and timing data generated in this work have been deposited in a *Zenodo* repository at (https://doi.org/10.5281/zenodo.19893169) under a Creative Commons Attribution 4.0 license.

## Code availability

The SSP-BO code is publicly available at (https://github.com/ctn-waterloo/ssp-bayesopt) under an MIT license. The robotics experiments code is available at (https://github.com/contactrika/bo-ssp) under an MIT license.
