# Supplementary material for: Compositional neurosymbolic representations enable efficient active exploration
Source: Nat Commun. 2026 Aug 22;17:10055. doi: 10.1038/s41467-026-75703-4 (PMC13594323; doi:10.1038/s41467-026-75703-4)
Supplement: Supplementary file 1 [file 41467_2026_75703_MOESM1_ESM.pdf]

Supplementary Material for
Compositional Neurosymbolic Representations Enable Efficient Active Exploration

Supplementary Note 1 Glossary of Essential Terms

Supplementary Table I provides a list of terms that are frequently mentioned in the paper.

Supplementary Table I: Explanation of the acronyms that are used frequently throughout the paper.

Acronym	Definition	Description
VSA	Vector Symbolic Architecture	A term coined by [58] to refer to a family of cognitive models that combine symbolic and connectionist architectures. Early examples include [149, 131, 126].
	Vector Symbolic Algebra	A term we adopt to distinguish the representations and algebraic operations of a VSA from the systems constructed using VSAs.
HDC	Hyperdimensional Computing	A more general term for VSAs and related models coined by [81] to emphasise that these models use hyperdimensional representations.
HRR	Holographic Reduced Representation	A specific example of a VSA introduced in [126].
SSP	Spatial Semantic Pointer	A hyperdimensional vector representation of continuous features specified as fractional binding in HRRs.
BO	Bayesian Optimization	A method for global optimization of black-box functions via iterative selection of sample points based on a probabilistic surrogate model.
BLR	Bayesian Linear Regression	An approach that combines linear regression with Bayesian inference, providing a probabilistic framework to model uncertainty in both the coefficients and predictions of the regression model.
GP	Gaussian Process	A probabilistic model that represents a distribution over functions, and is often used in BO.

Supplementary Note 2 Hardware

All non-neuromorphic experiments were run on compute clusters with 1 AMD EPYC 9655 (Zen 5) CPU. For the GPU-accelerated results (BoTorch and SVGP), 1 AMD EPYC 9454 (Zen 4) and 1 NVidia H100 80GB were used.

Neuromorphic experiments were run using SpiNNaker 1 and Loihi 2. SpiNNaker [83], released in 2011, is a massively parallel neuromorphic platform designed as a Globally Asynchronous, Locally Synchronous system. It uses energy-efficient digital ARM cores to simulate groups of spiking neurons with local memory and event-driven updates. Loihi 2 [120] – released in 2021 – is a fully digital spiking neural processor with 128 programmable cores. It supports custom neuron models, synaptic learning rules, and graded spike encoding, enabling real-time, low-power spiking computation via asynchronous networking and memory optimized for sparse connectivity.

In order to map the optimization circuit to neuromorphic hardware we use the Nengo software library [10] to compile the functional description of the optimization circuit into a population of neurons. To map the circuit to the SpiNNaker chip we used the `nengo_spinnaker` backend. To map the circuit to the Loihi 2 chip we wrote a custom script to translate the neural circuit to the Lava programming framework. On Loihi 2 we used ReLU neurons and took advantage of the graded spike capability to efficiently and allow for shorter simulation times. We converted synaptic weights to 8-bit signed fixed-point representations, using 4 fractional bits.

Supplementary Note 3 Baseline Methods

We compared SSP-BO to several BO baselines. Methods such as exact GP, BoTorch, and SVGP are standard and described in the main text. The RFF-BO baseline used in Supplementary Figure 4 was developed specifically for comparison to SSP-BO and is described in Algorithm 3. In the Supplementary Figure 5**a** and Supplementary Table I, we compare SSP-BO to a number of BO baselines for combinatorial tasks. These methods differ from each other in their choice of kernel and optimization method. The specifications of these methods are given in Supplementary Table II.

Supplementary Table II: A comparison of all methods used to generate the combinatorial optimization results shown in Supplementary Figure 5(a).

Algorithm	Kernel	Acquisition Function	Optimization Method	Optimization Space
SSP-BO	Similarity in feature space	Upper Confidence Bound	L-BFGS	Continuous, smooth
COMBO [118]	Diffusion kernel	Expected Improvement	Line Search	Combinatorial
BOiLS [65]	String Subsequence Kernel (SSK)	Expected Improvement	Hill climbing with trust region	Combinatorial
BODi [31]	Hamming embedding via dictionary kernel	Expected Improvement	Alternated between hill climbing and multi-armed bandit	Combinatorial
BOSS [115]	String Subsequence Kernel (SSK)	Expected Improvement	Genetic algorithm	Combinatorial
GP (SSK) [33]	String Subsequence Kernel (SSK)	Expected Improvement	Genetic algorithm with trust region	Combinatorial
GP (TO) [33]	Transformed Overlap	Expected Improvement	Genetic Algorithm with trust region	Combinatorial

Algorithm 3 RFF Bayesian Optimization (RFF-BO). N is the total sampling budget for the algorithm, β is the inverse variance parameter, α was chosen to be 0.01, $f(\cdot)$ is the target function being optimized, and $\mathcal{X}$ is the domain of $f(\cdot)$. γ_c is a scaling parameter that can be used to further control the influence of the exploration term.

procedure RFF-BO($N, f(\cdot), \frac{1}{\beta}, \alpha, \lambda$)
  $\mathbf{m}_0,\ \Sigma_0,\ \Sigma_0^{-1} \leftarrow \mathbf{0},\ \alpha\mathbf{I},\ \alpha^{-1}\mathbf{I}$
  for $k \leftarrow 1$ to N **do**
    $\mu_n(\mathbf{x}) \triangleq \mathbf{m}_n \cdot \phi(\mathbf{x})$
    $\sigma_n(\mathbf{x}) \triangleq \sqrt{1/\beta + \phi(\mathbf{x})^T \Sigma_{n-1} \phi(\mathbf{x})}$
    $\mathbf{x}_n \leftarrow \arg\max_{\mathbf{x}\in\mathcal{X}} \left[\mu_n(\mathbf{x}) + \lambda_n \sigma_n(\mathbf{x})\right]$
    $f_n \leftarrow f(\mathbf{x}_n)$
    $\mathbf{m}_n, \Sigma_n, \Sigma_n^{-1} \leftarrow \text{updateBLR}\left(\mathbf{m}_{n-1}, \Sigma_{n-1}, \Sigma_{n-1}^{-1}, \mathbf{x}_n, f_n\right)$
  end for
end procedure

Supplementary Note 4 Additional Results

4.1 Memory Complexity

Supplementary Figure 4**(d)** shows that the SSP-based algorithms have superior time complexity – constant in the number of samples collected. We can show that this property transfers to memory complexity as well. Supplementary Figure 1 captures how the memory requirements of the BO algorithms increase with the number of samples, relative to the initial heap memory size. Memory for logging data for both algorithms was pre-allocated and subtracted from the heap size after n samples. The GP-based methods have the anticipated quadratic memory growth, while the SSP-based methods have constant memory complexity.

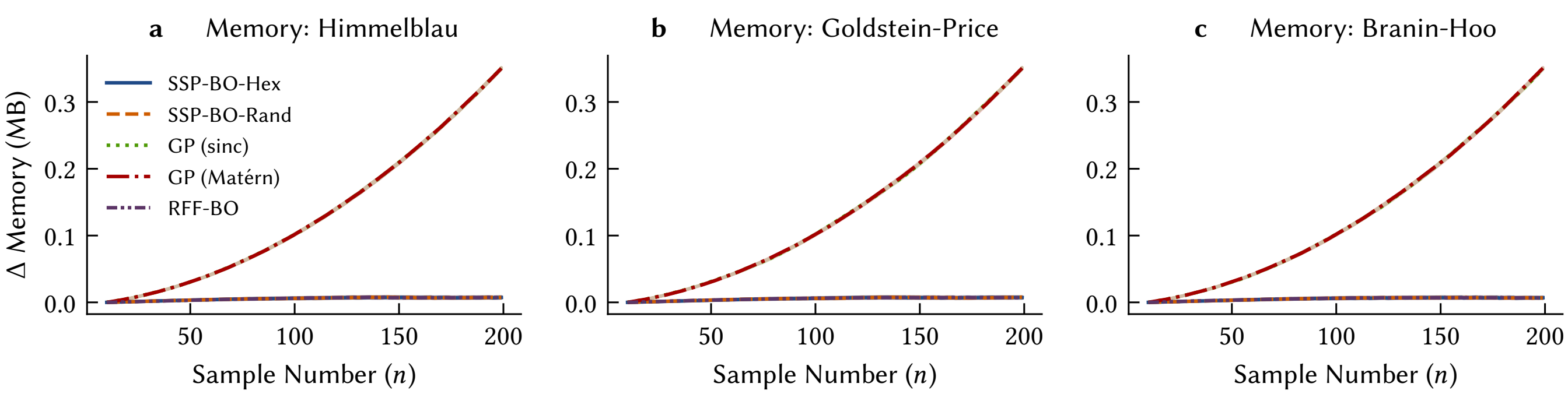

Supplementary Figure 1: The growth for the heap memory of the GP-BO and SSP-BO algorithms as a function of the number of samples collected. Values were computed by the heap size in megabytes (MB) minus the heap size before sampling begins. As anticipated, GP-MI has quadratic growth in memory requirements while SSP-BO has constant memory complexity. Shaded regions (not visible in the plot) represent a 95% confidence interval computed over 30 trials.

4.2 Terminal Regret Performance

Supplementary Figure 4**(a-c)** show the average regret of our algorithm using Hex-SSPs (SSP-BO-Hex) and Rand-SSPs (SSP-BO-Rand), compared to GP baselines using the sinc kernel and a Matérn kernel. By the end of the sampling budget of $N = 200$, we see that the SSP-BO algorithms are performing at least as well as, and in some cases, statistically significantly better than the GP-MI algorithms. Likewise, Supplementary Figure 5(a)i-ii shows the best objective value found (lower is better for these tasks) by SSP-BO, compared with 8 different GP baselines. Supplementary Figure 2 visualizes the terminal performance in these tasks with violin plots.

Overall, we found that the SSP-BO algorithm either lowered the terminal regret, or was statistically indistinguishable at a confidence level of $p < 0.01$ (Supplementary Figure 2). In the case of the Pest Control task, three algorithms had statistically better final performance at a confidence level of $p < 0.05$, but not $p < 0.01$. Significance was measured using a Wilcoxon signed rank test for 60 trials (test functions) or 10 trials (combinatorial tasks).

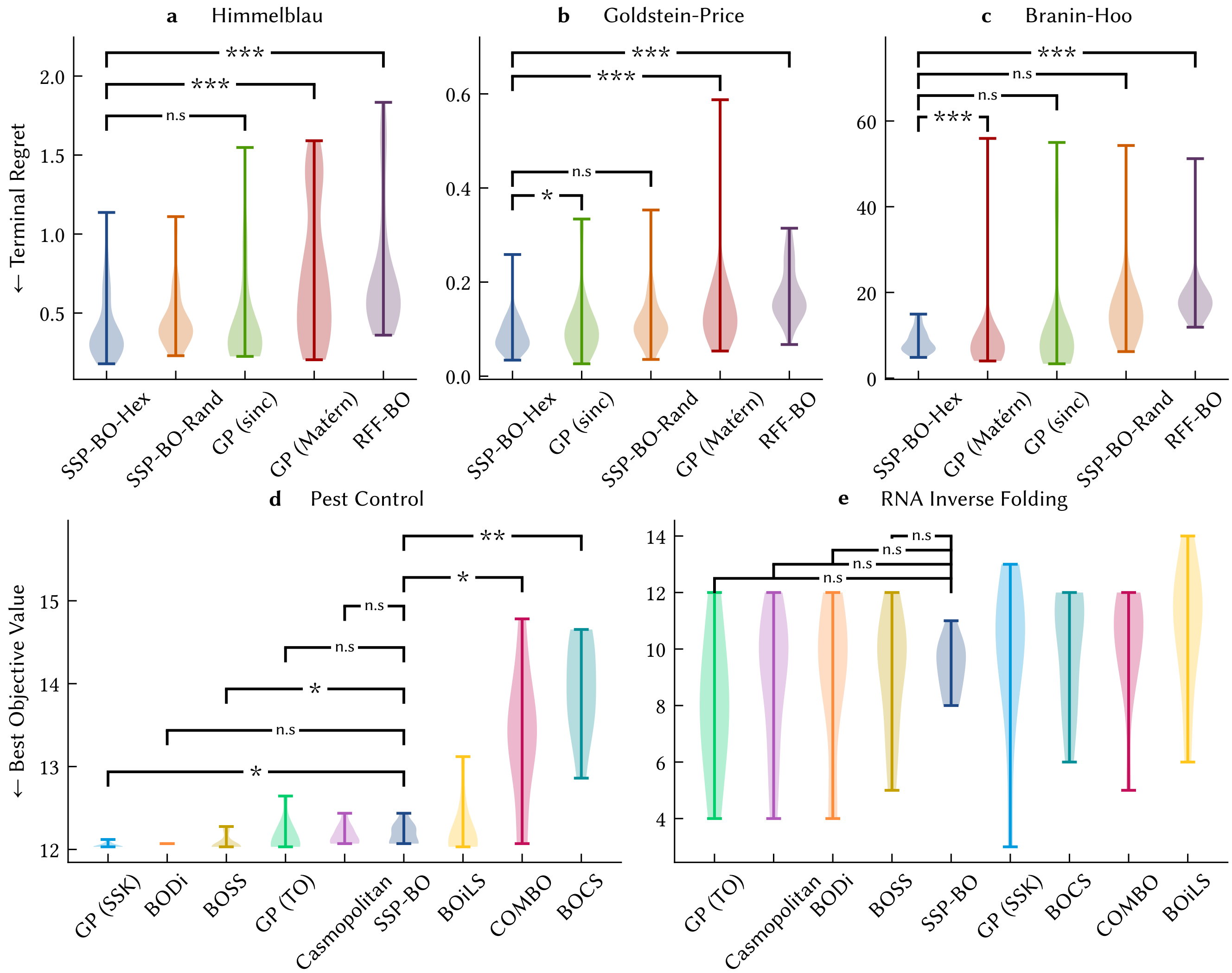

Supplementary Figure 2: **Terminal performance (regret or best objective value) for SSP-BO is either statistically significantly better or indistinguishable from baselines. (a-c)** Violin plots of the terminal regrets of the algorithms for the different test functions for 60 trials. **(d-e)** Violin plots of the best objective value found for for the combinatorial optimization functions for 10 trials. Algorithms are ordered by the mean performance for each task. Stars indicate level of statistical significance, using Wilcoxon signed rank test between pairs of algorithms with the null hypothesis that the right-sided algorithm is better performing. $* \implies p < 0.01, ** \implies p < 0.001, *** \implies p < 0.0001$, and n.s $\implies p > 0.05$ ($0.01 \leq p \leq 0.05$ is unmarked on the plots). We find that the SSP-BO-Hex algorithm either lowered the terminal performance, or was statistically indistinguishable at a confidence level of $p < 0.01$.

4.3 Dimensionality Experiments

Supplementary Figure 3 shows the robustness of Hex-SSPs to changes in the dimensionality. For the Hex-SSPs, we are able to substantially reduce the SSP dimensionality (from 151 to 25) before losing statistical equivalence with the best tested GP algorithm. For Rand-SSPs, the performance begins to degrade for $d < 100$, and becomes highly variable.

To assess how the required dimensionality scales with the data size in practice, we evaluated SSP-BO on the m-dimensional Styblinski–Tang function, varying m and embedding dimensionality d (Supplementary Figure 4). As shown in Supplementary Figure 4**a**, the terminal regret for all methods increases approximately linearly with the data dimensionality m. This trend reflects the increasing difficulty of optimizing in higher-dimensional spaces, where the search space volume grows exponentially.

Supplementary Figures 4**b–d** show heat maps of terminal regret as a function of both the data dimension m (x-axis) and the embedding dimension d (y-axis) for SSP-BO-Hex, SSP-BO-Rand, and RFF-BO, respectively. These results demonstrate that increasing the embedding size d generally improves performance, particularly at higher m, consistent with theoretical expectations: larger embeddings offer greater representational capacity, improving kernel approximations and decoding quality. Among the methods, SSP-BO-Rand appears most robust to variations in d and scales best with increasing m on this specific test function. Even with relatively small embeddings, SSP-BO-Rand maintains lower regret compared to RFF-BO.

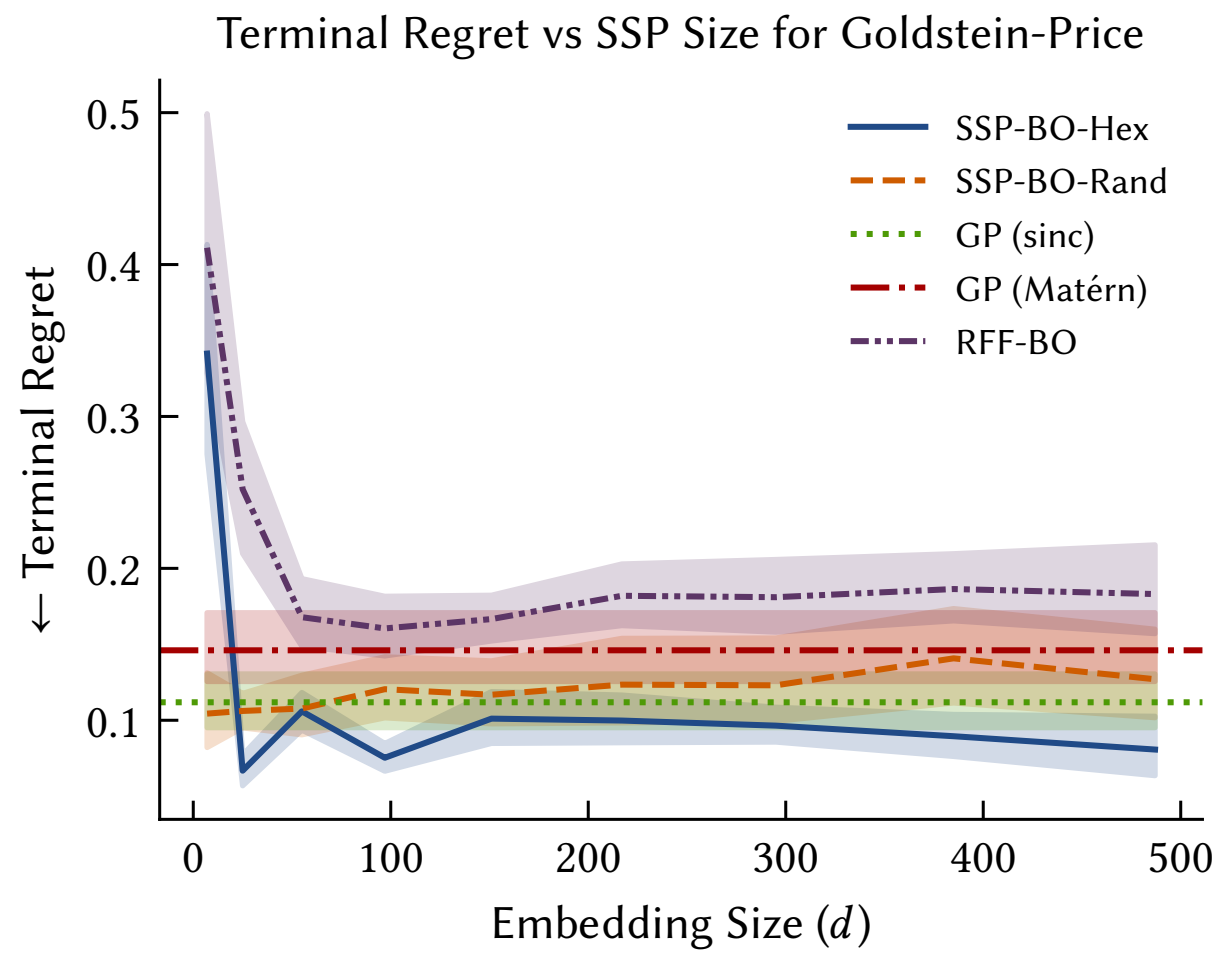

Supplementary Figure 3: **The terminal regret of SSP-BO using Hexagonal SSPs is robust to changes in dimensionality** The sensitivity of the SSP-BO algorithm to the dimensionality of the SSP representation on the Branin-Hoo function. For SSP-Hex, $\beta = -2.0 \times 10^{-4}$; for SSP-Rand, $\beta = 5.9 \times 10^{-5}$; for RFF-BO, $\beta = -2.1 \times 10^{-4}$ (β is the regression coefficient of a sensitivity analysis). Increases in SSP dimension correlate with decreases in terminal regret, with the Hex-SSP showing greater sensitivity to dimensionality. Shaded regions indicate 95% bootstrap confidence intervals from 30 trials. Solid black dots indicates SSP dimensions used in other experiments.

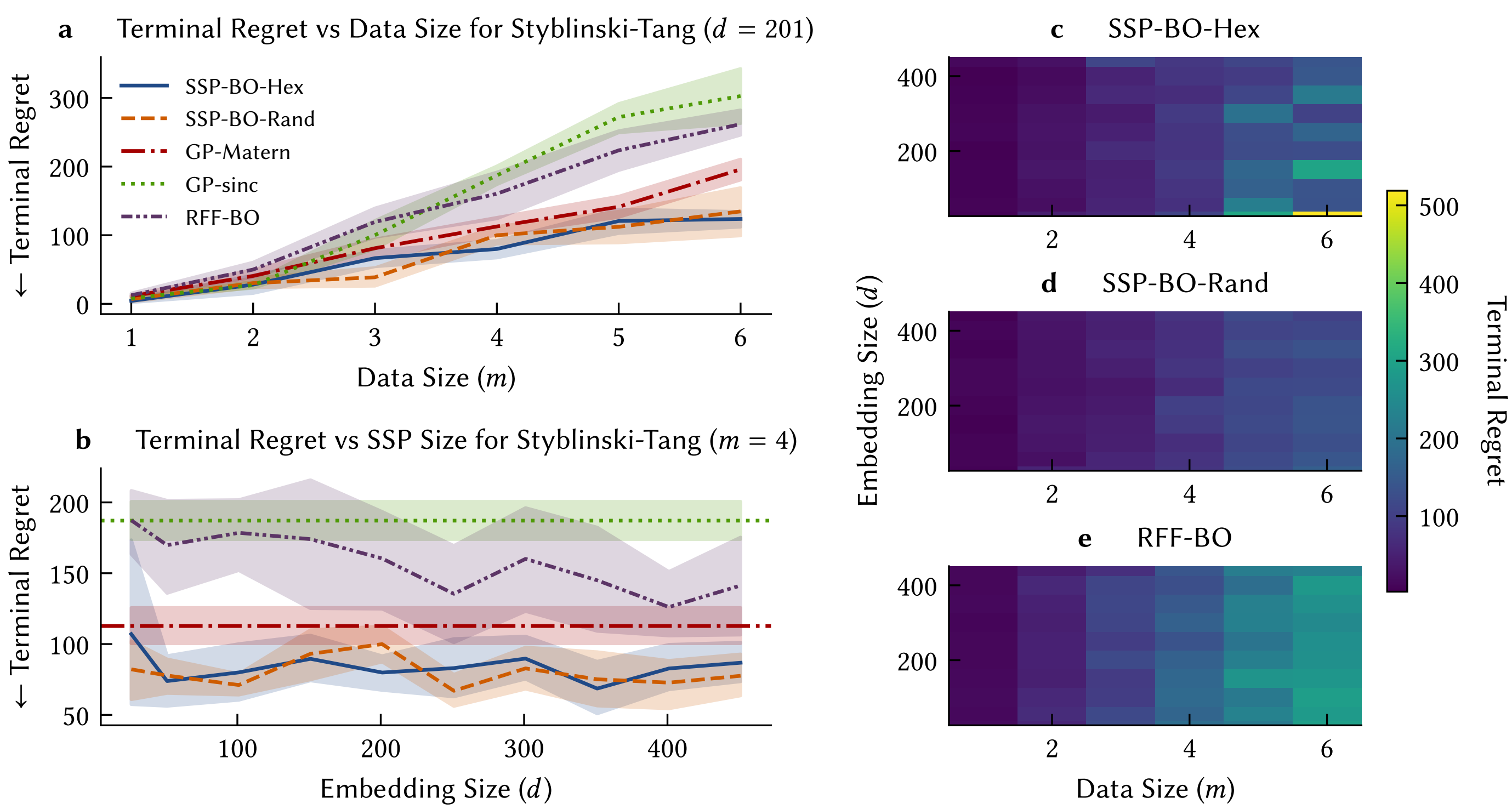

Supplementary Figure 4: **Performance scaling of SSP-BO with embedding dimensionality and data size. (a)** Average terminal regret on the Styblinski–Tang test function, $f : \mathbb{R}^m \to \mathbb{R}$, plotted as a function of the data size m. We compare two SSP-BO variants (Hex and Rand) against the GP baseline methods and RFF-BO. All embedding-based methods use fixed vector size $d = 101$. Since the HexSSP construction typically restricts the allowable values of d, its phase matrix was padded with random phases to match the embedding size of the other methods. While regret increases with m across all methods due to the exponentially increasing search space, SSP-BO methods outperform the baselines when $n \geq 3$. **(b)** Average terminal regret on the 4-d Styblinski–Tang test function plotted as a function of the d, size of vector embeddings (i.e., SSPs and RFFs). **(c-e)** Heat maps showing the final regret as a function of data size m (x-axis) and embedding size d (y-axis) for **(c)** SSP-BO-Hex, **(d)** SSP-BO-Rand, and **(e)** RFF-BO. Higher embedding dimensionality generally leads to lower regret, especially for larger n.

Supplementary Note 5 Choice of VSA

There are a number of VSAs that can be used to represent data in high-dimensional spaces, surveyed in detail by Schlegel et al. [141]. All of these algebras are designed to be compositional, by their very nature, but the choice of algebra constrains what data can be represented. Furthermore, there are limitations on the kinds of kernels that can be constructed. These limitations are imposed by the choice of binding operator and the base vectors the algebras operate on.

Three popular binding operators are tensor product [e.g., 150], circular convolution [e.g., 127], and element-wise

multiplication [e.g., 57]. All three distribute over vector addition, and with the right choice of base vectors, all three encoding schemes can implement a kernel product. However, to implement the techniques described above, there are two desirable characteristics: first, we should be able to iteratively bind vectors without limitations; second, the binding operation should admit a fractional interpretation.

The choice of base vectors can directly limit the representational capacity of the VSA. For example, using binary ($\phi \in \{0, 1\}^d$) or bipolar ($\phi \in \{-1, 1\}^d$) vectors limits the representational space to rational valued data. Similarly, in this work, we restrict ourselves to the space of unitary vectors – real-valued vectors whose Fourier components all have unit magnitude. If we did not make this choice we would not be able to apply iterative circular convolutions indefinitely, as the vector length would either shrink or grow with repeated application of the convolution operator. If one is using multiplicative binding with vectors other than unitary complex vectors (i.e., Plate's FHRR algebra), there would be a similar degradation of representations.

The tensor product does not preserve dimensionality, since each application of the tensor product raises the dimensionality of the representation, d, to a new value, i.e. $\bigotimes_{i=1}^{n} \mathbf{a}$ has dimensionality d^n. Similarly, a fractional interpretation is not readily available for the tensor product, which is also the case for the vector-derived transform binding operator of Gosmann and Eliasmith [63]. Consequently, given an agent with finite resources, there is an upper limit on the number of applications of an iterated tensor product.

Element-wise multiplicative binding does readily admit a fractional implementation – simple element-wise exponentiation. However, if the axis vectors are not complex numbers, it is not clear that this binding operation will correspond to a kernel product. Additionally, if the base representation is a bipolar vector, encoding a value which has a fractional component of the form 2^{-k}, $k \in \mathbb{Z}^+$, will cause the encoding to leave the space of bipolar vectors. If the base representation is a binary vector, fractional multiplicative binding in binary vectors will have no effect.

Permutation is another operation which can be used to encode integer data, up to the limit of the dimensionality of the vector, and again, it does not have a clear fractional interpretation. The naive interpretation – raising the permutation matrix's eigenvectors to the power of the encoded value – can cause the encoded vector to leave the binary or bipolar vector spaces.

Consequently, we conjecture that the HRR (or FHRR) with unitary vectors is the type of Vector Symbolic Algebras best suited to the modelling of compositional kernels. However, that does not mean that other algebras cannot be used in probabilistic modelling. For example, Smolensky's tensor product is the same operator as used in quantum probability [e.g., 132]. It may be that other algebras and representations can make strong predictions about the kinds of probabilities that can be modelled neurally, and may have different application-specific uses.

Supplementary Note 6 Complexity Analysis

Below, we provide details about the asymptotic time complexity of the SSP-BO algorithm. The algorithm has four distinct steps:

- Selection of the optimal point via gradient optimization: complexity $O(d^2)$;
- Encoding the observed sample point: complexity $O(d^2)$;
- Updating the BLR: $O(d^2)$ by virtue of the rank one updates to the inverted matrix;
- Decoding of the selected next sampling location: $O(dh)$ when using a neural network to decode, where h is the number of hidden units in the network.

Provided $h \leq d$ then the dominating term is $O(d^2)$. In all experiments we used $h = 8$, meaning that for this implementation the decoding complexity is dominated by the first three steps, since $d = 151$.

Comparing this to GP-BO, we note that the naive approach of implementing GP-BO requires inverting the covariance matrix (via Cholesky decomposition), which has an $O(n^3)$ time complexity. Consequently, the instantaneous sample point selection for SSP-BO is an improvement over naive GP-BO when $d < n^{\frac{3}{2}}$. However, the value of n changes during operations. To figure out which algorithm is superior, we analyze the total time complexity for a given sampling budget of N. SSP-BO dominates when it's total complexity is less than that of GP-BO:

$$\sum_{t=1}^{N} d^2 \leq \sum_{t=1}^{N} t^3$$
$$\implies d \leq \sqrt{\frac{N^3}{4} + \frac{N^2}{2} + \frac{N}{4}}$$

Supplementary Note 7 Neuroscience and cognition

The results presented in this paper show the pragmatic benefits of the SSP-BO algorithm in the context of robotics and reinforcement learning (RL), but it has other intriguing aspects. The techniques that we use in this paper originated in

cognitive science and neuroscience. As a result, the work also provides hypotheses about cognition that can be grounded in hypotheses about biology.

From a neuroscience perspective, the SSP-BO algorithm is a plausible model of planning in the brain that incorporates grid cells. We believe that SSP-BO is neurologically plausible for two core reasons: 1) we are building our representations using a mathematical model of neural representations of state; and 2) statements in our chosen VSA can be directly translated into networks of spiking neurons and the Hex-SSP representation can be encoded by the spiking activity of grid cells [35].

To the first point, we have demonstrated that the Hex-SSPs support the BO algorithm with vectors of lower dimensionality than randomly selected vectors. They were designed to replicate the hexagonal firing grid pattern found in grid cells in the medial entorhinal cortex (MEC) [35]. Hexagonal grid representations found in the hippocampal formation are known to represent a number of continuous parameters like place, head direction, and eye direction [114]. Their efficiency in our tasks, compared to purely random encodings, speaks to why they arise in biology and to why they have been observed to arise in deep RL tasks [8].

It has further been hypothesized that grid-cell like representations may extend to other areas of cognition, including language [9]. Our work optimizing over structured representations lends some credence to this hypothesis. Because we are approximating GPs over compositional structures, our work connects to cognitive science, and through the VSAs connection to neural networks, situates such hypotheses directly within neurobiology.

To the second point, VSA representations have been mapped to populations of spiking neurons [40, 146]. Indeed, we demonstrate that the sample selection step of SSP-BO can be performed by a recurrent spiking neural network in . The network architectures implied by VSA statements – including the proposed recurrent network – are hypotheses about neural structure and animal behavior. In this way, our work provides not only a resource-efficient exploration algorithm, but also a hypothesis about exploration in biological systems.

The neural plausibility of our work also provides a connection between normative models of human inference and biological structures. Lucas et al. [107] observed that GPs unify two schools of learning in organisms: rule based (symbolic) representations, and similarity based representations. Since GPs can be understood in terms of kernel functions (which impose strong inductive biases) and feature vector representations (which align with neurological representations), they bridge the gap between symbolic reasoning and the similarity-based inference of neural networks – precisely the same role VSAs were designed to play. This connection helps to explain the ability of humans to learn from small sample sizes while still operating in spaces representable by neural networks. Schulz et al. [143] expanded on the work of Lucas et al. [107] and Wilson et al. [172] by exploring the space of compositional kernels defined by the grammar of Duvenaud et al. [39]. They found that compositional kernels can explain human inference capabilities, while operating in the infinite dimensional space implied by exact kernel functions.

What is lacking from the proposed normative model in [107] and [143] is an explanatory model of how composition may be implemented by biological organisms. What we provide is a similarity-based system where one does not have to leave the finite-dimensional space to implement these rules. We can implement the same grammar as Duvenaud et al. [39], and our compositional methods act as a strong hypothesis regarding neurological structures and processes. For each VSA statement about a data encoding, there is at least one neural network that performs this encoding, and these encodings are exactly finite-dimensional approximations of the kinds of compositions that Shulz et al. have proposed as a normative model of cognition.

Our framework is a language for defining compositional kernels that defines neural network structures, which also replicates observations of state space representations found in human biology and in networks trained in RL settings. It has been established that reward prediction errors transmitted in biological brains resemble some aspects of model-free RL [74]. The evidence of model-based reasoning has also been found in biological systems [60]. However, the majority of modern model-free RL methods for non-biological systems are notoriously sample-inefficient [136], while model-based methods that have the potential to learn models needed for complex reasoning and physical interactions tend to also require a significant amount of memory. Concretely, state-of-the art deep RL methods, such as PPO [142] and SAC [66], need one-to-two orders of magnitude larger number of environment interaction steps than our method. Model-based methods can be more data-efficient, but scalable versions require much more memory. For example, [21] outperformed a number of model-based and model-free methods in data efficiency, but even for simpler problems it required an order of magnitude more memory than our method, even for a single expert in its mixture-of-experts ensemble. We aim to build data- and memory-efficient learning methods, which also have the potential to be biologically plausible. Here, we have demonstrated advances in the framework of model-free data-efficient active learning. In future work, we aim to explore enhancing model-based RL methods, starting with those based on Gaussian processes, such as [27]. SSP-based representations we developed can improve scalability, reduce memory consumption, leverage compositionality, and move the resulting methods closer to biological plausibility.

Indeed, our framework is a natural extension of the Semantic Pointer Architecture (SPA), which has been used to build spiking neural networks that are large-scale brain models exhibiting cognitive phenomena. With the expansion we propose here, we suspect that the SPA fills the gap between normative models of human function learning and inference, and implementations in the biological computational substrate that we know must generate these behaviours.

Finally, the conjecture that VSAs explain compositional function learning suggests further research directions. Specifically, finite dimensional random feature embeddings have known limitations, in the form of maximum capacities that can be stored in a given vector representation, errors in approximating the true underlying kernel, and how numerical precision requirements interact with the bounded firing rates of the neurons that must be representing these vectors. Clearly, human rationality is also bounded. It is thus interesting to consider whether the limitations of random feature embeddings induce the bounded human capabilities for learning and exploring. Having a bridge between the abstract, mathematically pure models of Lucas et al. [107] and Schulz et al. [143], and a biologically constrained model is necessary to further explore these questions.
