# Supplementary material for: Compositional neurosymbolic representations enable efficient active exploration
Source: Nat Commun. 2026 Aug 22;17:10055. doi: 10.1038/s41467-026-75703-4 (PMC13594323; doi:10.1038/s41467-026-75703-4)
Supplement: Supplementary file 2 [file 41467_2026_75703_MOESM2_ESM.pdf]

nature portfolio

Peer Review File

Compositional Neurosymbolic Representations Enable Efficient Active Exploration

Corresponding Author: Dr P. Michael Furlong

This file contains all reviewer reports in order by version, followed by all author rebuttals in order by version.

Attachments originally included by the reviewers as part of their assessment can be found at the end of this file.

Version 0:

Reviewer comments:

Reviewer #1

(Remarks to the Author)
This manuscript presents a novel approach to address the challenges of continual learning and exploration in AI, particularly in complex and high-dimensional spaces. The authors introduce a compositional neurosymbolic method that bridges biological and synthetic reasoning, applying it to Bayesian optimization (BO) tasks in robotics and learning. Their algorithm, SSP-BO, utilizes Vector Symbolic Algebras (VSAs) to achieve significant reductions in time and memory complexity while maintaining accuracy. The method transforms the BO sample selection problem into a simple convex optimization problem, enabling efficient computation even in real-world systems with limited resources. The authors report up to 175x reduction in compute time compared to state-of-the-art frameworks like BoTorch, making it a promising solution for autonomous systems requiring lifelong learning capabilities. While the paper presents a promising approach with significant improvements in computational efficiency and accuracy, several weaknesses and areas for improvement can be identified.

- The proposed method involves a sophisticated integration of compositional neurosymbolic representations and Bayesian optimization. This complexity might make it challenging to implement and understand for practitioners who are not familiar with neurosymbolic programming and Vector Symbolic Algebras (VSAs).

- The comparison with state-of-the-art methods like BoTorch is promising, but a more detailed comparative analysis with a broader range of existing techniques in Bayesian optimization and reinforcement learning could provide a clearer picture of the advantages and limitations of SSP-BO. This includes performance metrics, resource consumption, and applicability to different types of problems.

- The paper acknowledges the current inability to handle concept drift and change hyperparameters online. This limitation can be significant in real-world applications where environmental conditions and system dynamics frequently change, necessitating adaptive learning capabilities.

- The decoding process, which converts the optimal vector back to the domain space, relies on pre-trained neural networks. This introduces additional complexity and potential sources of error, especially when the length scales change. The need to retrain the network for different scales can be seen as a limitation in dynamic environments.

- The paper briefly discusses the trade-offs in approximation accuracy. The reliance on VSAs and the induced kernels might introduce approximation errors that can affect the overall performance, especially in highly sensitive applications.

- The practical integration of SSP-BO with existing autonomous systems and frameworks is not extensively covered in the manuscript. Understanding how this method can be seamlessly integrated into current robotics and AI pipelines would be beneficial for wider adoption.

- The paper lacks a detailed error analysis of the results. Understanding the types and sources of errors, especially in complex environments, would help in identifying the strengths and limitations of the proposed method. This includes investigating failure cases and analyzing why SSP-BO outperforms or underperforms in certain scenarios.

- The reported results focus on the average performance and improvements in computational efficiency. However, there is limited discussion on the robustness and stability of SSP-BO under varying conditions, such as noisy data, dynamic changes in the environment, and adversarial conditions.

- No comparison with hardware-based designs (ASIC, FPGA, in-memory) is provided.

- The authors only discuss reduction in computation time as the important benefit from their method. How about power and energy consumption?

- The results are primarily based on simulated environments. Validating SSP-BO in real-world settings with physical robots and actual sensors would provide stronger evidence of its practical applicability and effectiveness.

- It surprises me that “Section II. Results” appears right after the introduction section. Is this a requirement for this journal? Otherwise, it is expected from the methodology is discussed and then we see the results. The paper organization can be improved.

- The baseline framework in this work is BoTorch. While reference [4] is provided on page 15 for BoTorch, its discussion starts way earlier in the paper. The reference should appear sooner.

- But why BoTorch is selected as the baseline in this work? More justification is needed to accept BoTorch is the state-of-the-art framework with best performance in this area.

- Table I appears in page 4 but it’s cross-referenced for the first time in page 5.

(Remarks on code availability)

Reviewer #2

(Remarks to the Author)
This paper proposes a spatial semantic pointer (SSP) based Bayesian optimization (BO) framework that reduces Gaussian process BO's time and memory complexity while maintaining a high reward performance. The key ingredient is to use fractional power encoding (FPE) with hexagonal grid representation, yielding a novel inner-product kernel that can be used with Bayesian linear regression for optimization. Experiments are conducted on synthetic test functions as well as multi-agent robotic simulations.

In general, the paper is very well written, and the application of vector symbolic architectures (VSAs) seems to be meaningful in BO. However, I have some concerns that may be answered in a revised version of the manuscript:

Novelty
The key concept of using VSA for BO has already been proposed in [39]. This manuscript mainly explores an alternative hexagonal kernel [23] (instead of a random one) to encode multiple feature dimensions. Apart from additional robotic benchmarks that required multi-trajectory exploration, this manuscript does not seem to introduce a sufficiently novel concept. It would be helpful if the authors could explicitly state the novelties introduced in this paper, given the already impactful work in [23] and [39].

Practical applications/motivation
The introduction motivates Bayesian optimization (BO) for reinforcement learning and optimizing hyperparameters of machine learning algorithms. Reducing BO’s complexity at iso-accuracy is obviously a useful thing to do; however, the paper does not explicitly state where the presented approach revolutionizes intelligent systems. Hence, it would be helpful to propose/show applications where SSP-BO can solve a problem that has not been possible with the previous methods (e.g., on specific hardware). In hyperparameter search for ML models, SSP-BO’s advantage might not be significant as the bottleneck in hyperparameter search will instead be in the environment evaluation (i.e., the ML model training). Moreover, the memory requirements of SSP-BO are higher than BOTorch in practical domains: experiments on PSR (24D) reach convergence after n=100 samples (Fig. 3c), whereby the SSP-BO exceeds the memory requirements for n<187.

Missing comparison to efficient SOTA BO
The results mainly compare SSP-BO with vanilla BO, whereby optimized versions are ignored (e.g., Sparse GPs or discretized state spaces mentioned in the introduction). It would be fair to include these methods in the experiments.

Other questions/remarks
Is there a specific reason to use the sinc-kernel (i.e., uniform phase distribution) for the base vectors? Could you consider other alternatives kernels (shown in [34])?

I could not find a direct relationship/approximation between GP and the SSP kernel, as one knows typically from the RBF

kernel. What exactly does the SSP kernel approximate? Is it simply a kernel that might work well in these scenarios with no direct relationship to a Gaussian process?

It would be helpful to describe m_n and Σ_n, used in Eq. (2) and Eq. (3), after or before the equations.

Counterintuitively, the total time in Table I is much shorter for more complex robotics applications compared to synthetic test functions. I guess this could be due to the different number of samples. It would be interesting to see how the method scales with the number of state space dimensionalities, e.g., by having the same number of samples across different tasks.

I could not find any results in the robotic simulations using the SSP-BO-Rand. It would be good to show the superiority of SSP-BO-Hex by having this baseline, too.

Appendix E states that a neuromorphic computer would enable the advertised complexity of O(1); however, the current experiments were run on a traditional computer with O(d^2) (or O(d) when properly utilizing the GPU). It would also be good to make this assumption clear in the main text and abstract.

The ablation in Fig. 11 shows that the vector dimensionality can be as low as d~30 at iso-accuracy. This is quite surprising as VSAs typically require a dimensionality of hundreds or thousands. How can the kernel approximation still work in these low dimensions? Is it because we do not have any kernel addition in this example? What is the behavior of robotic applications?

(Remarks on code availability)

Reviewer #3

(Remarks to the Author)
Given that the primary purpose of this review is to provide feedback on the soundness of the research reported in order to help the authors to improve their manuscript and the editors to reach a decision, I am in a slightly odd position; I think the reported research is sound and there are no simple suggestions I could make for improving the manuscript. I could document that very briefly (which I will do below) and be done. However, I think this manuscript has hidden depths which I would like to discuss because it may assist the editors. (It may also be of interest to the authors, although I presume they are already more than aware of this depth.)

Allow me to start by stating my limitations. I have considerable experience in the field of Vector Symbolic Architectures/Algebras (VSAs), limited skills in mathematics, and only a passing acquaintance with Bayesian optimisation for learning and robotics tasks. The authors' claims with respect to VSA are consistent with the state of the art in the field (to which the authors have contributed significantly). I am not really qualified to assess the authors' claims with respect to Bayesian optimisation, however, there are no obvious red flags and the claims are supported by the empirical results. So, I am happy that the results claimed are reasonable and supported by the reported work.

My brief report organised under the pro forma headings:

* What are the noteworthy results?

- Development of a VSA representational scheme for compositional structured entities with discrete and continuous components able to be used in a Bayesian optimisation process and also compatible with neuromorphic implementation (spiking neuron neural networks). This is important because optimisation over mixed and discrete and continuous spaces can be problematic. (VSA avoids this by representing all structures in a continuous vector space.)

- Development of a VSA representational scheme for spatial representation that is consistent with current knowledge of spatial representation in the hippocampus. The value of this is tha the representation has valuable mathematical properties which can serve as an explanation for why the hippocampus behaves the way it does.

- Demonstrating constant time and memory complexity of the Bayesian optimisation process. This is a big deal, both practically, for anyone implementing a BO on a constrained computational device (e.g. edge computing / internet of things), and theoretically, because cognitive scientists should take computational complexity and this manuscript demonstrates that using a VSA representation can dramatically change the complexity.

* Will the work be of significance to the field and related fields? How does it compare to the established literature? If the work is not original, please provide relevant references.

- This work will be of significance in the field of Bayesian optimisation because it demonstrates how to achieve constant computational complexity. It is potentially significant in a wide range of other fields because it demonstrates how VSAs can be applied to problems to achieve computational advantage (see meta discussion below).

- Based on the cited works, it extends the current BO literature by using a completely new (VSA based) representation. This appears to be completely new in that field.

* Does the work support the conclusions and claims, or is additional evidence needed?

- The reported work supports the conclusions and claims. The claims can be simplified to: It is possible to build a VSA-based Bayesian optimisation process with space and time computational complexity that is constant. The theoretical analysis and empirical results clearly support this claim. (Viewing the work as a proof-of-concept demonstration, the empirical component is conceptually straightforward: Can I build it? Yes; Does it do what it is supposed to? Yes; How well does it perform the chosen tasks? At least as well as the standard alternatives; What are the computational costs? Less than the standard alternatives.)

* Are there any flaws in the data analysis, interpretation and conclusions? - Do these prohibit publication or require revision?

- No. Again, viewing the work as a proof-of-concept demonstration, the empirical component is conceptually straightforward: Design the system; Design the tasks; Simulate the system executing the tasks and gather as much data as you need. (Of course there is a great deal of detail to be attended to, which appears to have been done successfully.)

* Is the methodology sound? Does the work meet the expected standards in your field?

- Yes. There are two broad component here: the design and justification of the VSA system and the empirical assessment of the system. The structure and content of the manuscript with respect to the design and justification of the VSA system is completely compatible with standard practice in the VSA field and there is nothing that would cause any raised eyebrows. Likewise, the structure and content of the manuscript with respect to the empirical assessment of the system appears completely standard with respect to empirical computational research (e.g. machine learning).

* Is there enough detail provided in the methods for the work to be reproduced?

- Yes. The equations and algorithms in the manuscript provide enough information for the conceptual kernel of the system. As usual, an actual implementation requires many detailed (and theoretically irrelevant) decisions on how to instantiate the conceptual model. I expect this level of detail will be available when “Source code for the SSP-BO algorithm and code to re-produce the experiments with environments and baseline
algorithms [is] made public on publication.”

=====

My more meta-level thoughts on the manuscript:

Let me start by making an analogy between VSA and complex numbers. The heart of the VSA field is working out what VSA representations and operations are and how they relate to each other. Similarly, the heart of complex numbers and the operations on them is essentially about their definitions and internal consistency. However, the primary value of complex numbers lies outside purely internal considerations: How can they be used elsewhere in mathematics? What possibilities do they enable? What practical applications do they enable? How should they be used in practical applications? Mapping back to the VSA domain: the basic principles of VSA have been known for some while and the current research frontier is in teasing out further implications within VSA and working out how to apply VSA practically. Just as complex numbers enable a vast universe of mathematical applications, so VSA (as an unconventional basis for computation) is expected to enable a vast universe of computational applications.

This manuscript can be read on multiple levels. The surface reading is that it is about a VSA-based system for Bayesian optimisation for active exploration. The deeper level reading is that it is about the utility, adaptability, and value of VSA as a basis for implementing computational systems. Because it’s not a book-length manuscript it uses the tactic of providing a specific example (Bayesian optimisation for active exploration) and implicitly invites the reader to generalise beyond that example.

Taking that deeper reading there are some further interesting points in the manuscript not captured in the pro forma assessment above:

- The principal conceptual work component of this manuscript is a design process: How to design a VSA-based computational mechanism that achieves the computational objectives?

- The design of the VSA system for Bayesian optimisation is a technical tour de force. This is a complex mechanism with multiple moving parts. These all have to be designed to be compatible with each other and jointly achieve the computational objective of the entire system. Unlike traditional neural networks (e.g. deep learning networks) with vast numbers of trainable parameters, VSA systems have no or few trainable parameters and when they work it is because they have been designed correctly rather than by having enough parameters to do anything given enough computer power and data for parameter optimisation.

- The manuscript exposes much of the reasoning behind the design process. This is very valuable for readers trying to understand VSA and how they might apply it to their own problem domains. (Many papers are inclined to present the resulting mechanism without giving any insight into the design process.)

- The manuscript goes to considerable lengths to make links to other fields, e.g. machine learning, psychology, cognitive science, robotics, neuromorphic computing, and neuroscience. The citations throughout the paper are wide ranging. I found the Appendix from Section D onwards to be very interesting in this respect.

I hope these notes have been of use to someone in the exercise.

Ross W. Gayler

(Remarks on code availability)
Code not available until publication and even then I am probably not in a position to review it.

Reviewer #4

(Remarks to the Author)
Summary

The gist of this paper is to approximate Gaussian process (GP) regression by running standard Bayesian linear regression on VSA encodings of data. The highlights, as reported at the end of Section 1, are
(1) Complexity: The standard formulation of GP regression on n data points requires O(n^3) time and O(n^2) space. By approximating this using a linear regression on the d-dimensional VSA encodings φ(x), this becomes O(d^3) and O(d^2) respectively. Provided we can obtain a satisfactory approximation for d < n, this is better from the perspective of time and space complexity.
(2) Integrating Heterogeneous Types: The machinery of VSA bundling and binding provides a natural way to integrate heterogeneous inputs by bundling and binding their encodings. This has a natural interpretation in terms of the underlying kernel functions being approximated.
(3) Possible Connections to Neuroscience: The coding rules used here have been used (by the same authors) to model aspects of the hippocampus.

General comments

This has the potential to be an interesting paper, however it is poorly written, and does not frame its claims within the context of things that are already well-known and studied in the machine learning literature. Overall, the paper feels like a rush job, and I don't get the feeling the authors put much thought into how to communicate these ideas to a broad audience such as Nature Communications. For example the description of the BO problem being solved is extremely terse, and how these variables are being represented by high-d vectors will not at all be obvious to the reader not already acquainted with VSA. In addition the machinery of bundling and binding is buried in the appendix, not in the body of the paper, which would seem to take the wind out of the sails of the "compositionality" concept touted in the title and intro of the paper. In fact, how compositionality relates at all to the BO problem the authors are solving is entirely unclear just from reading the body of the paper.

In terms of relating to the machine literature - the idea of approximating a kernel function using dot-products between random embeddings of modest dimension is widely studied and explored in the ML literature. There are by now dozens of papers on this topic in the context of kernel machines in general (Rahimi and Recht, 2008; Lopez-Paz et al., 2014; Li et al., 2018), and several which discuss this in the context of GP specifically (Lazaro-Gredilla et al., 2010; Gal and Turner, 2015; Hensman et al., 2018). So, I feel there is a need to more clearly flesh out the messaging of this paper to a broader audience: the general strategy being advocated here is, to a large extent, what people already would do. Moreover, the specific random embedding method used here is, as the authors state, closely related to random Fourier features, which is a particularly widely studied instance of the principle described above. However, this fact is only stated (and even then obliquely) on page (9) in the methods section. These connections should be stated bluntly and clearly early on: there is a large body of work in the broader machine learning community that applies very similar methods to achieve very similar ends vis-a-vis efficient realizations of kernel methods in general, and specifically, Gaussian Processes. Without this contextualization, it is difficult to understand why the framing in terms of VSA is helpful or should be of interest to the broader community. If the answer is item (2) above, then state this and let the reader decide how excited they are about it.

Specific/technical comments

• Abstract: "We apply our approach to Bayesian optimization in complex spaces, outperforming state-of-the-art algorithms...while reducing the time and memory complexity from O(n^3) and O(n^2) to O(1)." It would be more accurate to say "reducing time and memory complexity from O(n^3) and O(n^2) to O(d^3) and O(d^2)," where d is the hypervector dimension. Moreover, the statement as written is only accurate if d does not depend on n. It's not clear this is true: most of the available theory on RFF/VSA indicates that there is, in general, a dependence of d on n, or some other fundamental property of the input space. For results of this nature in RFF see (Rahimi and Recht, 2008; Bach, 2017). In the context of VSA see (Thomas et al., 2021; Clarkson et al., 2023). Reducing this to an O(1) complexity is a bit of an oversimplification.
• Top of page 2: "From this we gain several benefits: 1) time and memory efficiency by transforming kernel machines to linear methods; 2) unified representations of mixed discrete and continuous spaces; and 3) an algebraic framework for understanding the content of neural activities." Re (1), see comment above w.r.t random feature models in general.
• Top of page 2: "Additionally, by operating in the high-dimensional vector space, using VSAs recasts the BO sample selection problem as a convex optimization problem, instead of an NP-hard optimization." This statement makes it sound like VSAs have managed to obtain a tractable solution to an NP hard problem. Rather, a more accurate description would be

that the reward function is being approximated using a GP, which it is possible to estimate and sample from efficiently (e.g. in polynomial time). This idea, approximating an NP-hard objective by solving a tractable surrogate problem, is foundational to stat ML and doesn't have anything to do with VSA per-se. The step involving VSA is a second layer of approximation that uses the idea of RFF/random features to approximate a GP using Bayesian linear regression in a random feature space. See also the note below about convexity.

• Top of page 3: "In the introduction above, we noted that the vector dot product induces an admissible kernel, or similarity function, when we represent continuous valued data using SSPs [119, 39, 23]." The dot-product between two vector-representations of data is always a positive-semidefinite function and hence an admissible kernel in the ML sense of the word (see Lemma 14 here:
https://www.gatsby.ucl.ac.uk/~gretton/coursefiles/lecture4_introToRKHS.pdf).
Thus, this statement is true for any embedding, be it continuous, an SSP, or otherwise, and hence seems vacuous. I believe what is meant here is that the dot-product between two VSA embeddings approximates another kernel of interest. Note as well that there is nothing preventing one from approximating a continuous kernel using discontinuous embeddings (also a widely studied topic in RFF e.g. (Raginsky and Lazebnik, 2009)) and so continuity of the embeddings doesn't seem an obviously important property here.

• Top of page 3: "We also showed that VSA encodings can replace kernel functions, $k(x, x') \approx \varphi(x) \cdot \varphi(x')$ in the style of the kernel trick [98]." Several comments:

– The citation [98] is to (Rahimi and Recht, 2008). It is weird to say "We also showed" and then to cite a paper by different authors that makes no mention of VSA. Also, this oblique reference to RFF is the only mention of the technique in the main body of the paper. FYI: per the bibliography at the end of Chapter 12 of (Wainwright, 2019), the "kernel trick" is usually attributed to (Aizerman, 1964).

– The kernel trick is explicitly about not materializing the embeddings and working with the kernel function directly. This is the opposite of what is being done here: approximating a kernel by an explicit embedding. It is another "trick" related to kernels, but it is not the kernel trick.

– There are many others in the VSA community who have noted the connection between VSAs and kernel approximation. The earliest of which I'm aware is (Plate, 2003) who talked about his HRR as an approximate form of string kernel. The connection between VSA and RFF/Bochner's Theorem was described in (Thomas et al., 2021), and in the subsequent work of (Frady et al., 2021) who also discussed the algebraic interpretations of bundling and binding in the context of RKHS functions represented using random features. These should be mentioned in this context.

• Top of page 3: "By approximating the kernel, we have transformed the sample selection problem into a convex optimization problem." It is not clear how this makes the sample selection problem convex. While $m \cdot \varphi(x)$ is linear in $\varphi(x)$, it is still a nonlinear, possibly non-convex, function of x over which the optimization is defined. I believe this is related to the topic of the final bullet in this list. Please see the comment there as well.

• Page 4: "In these scenarios, we test the SSP-BO algorithm against a state-of-the-art baseline, BoTorch, in three tasks relevant to robotics and machine learning." On page 6 the authors make a note of RFF as a related kernel approximation method, so it seems like this would be a salient comparison here. Moreover, there are existing Python packages that do this:
https://www. tensorflow.org/api_docs/python/tfm/nlp/layers/RandomFeatureGaussianProcess
and
https://pypi.org/project/pyrff/

• Page 6: "One related kernel approximation method, RFFs [98], offers good empirical perfor- mance in many cases, but its accuracy is ultimately limited by the number of features. Since VSAs can be viewed as a superset of RFFs, investigation is needed to assess the bounds on their representational capacity." Several comments:

– VSA is a broad term for a variety of different methods for systematically manipulating vector representations of data. Are the specific VSA techniques used in this paper a superset of RFF? This is a strong claim, and I'd like to see this proven as I'm not sure that it is, in fact, true.

– I see no reason why VSA is not also so limited. All available theory about VSAs indicates that they attain similar rates for kernel approximation as are known for RFF (Thomas et al., 2021; Clarkson et al., 2023). It's a stretch to use this to contrast the present work with RFF.

– This seems like an easy question to investigate empirically: just run both for a bunch of values for the dimension and plot the regret/loss they attain.

• Page 9: "Since fractional binding relates directly to the method of Random Fourier Features [98], we can appeal to that extensive literature for the design of particular kernel shapes." What does "relates directly" mean? If they are the same, just say so. If they are different, explain how, and why we can appeal to the literature of the RFF community to understand theoretical properties of the coding scheme in this paper nonetheless.

• Top of page 12: "We appeal to the method of Random Fourier features to confirm that we can shape the kernels to our liking, as demonstrated by Frady et al. [34], Komer et al. [63], and Dumont and Eliasmith [23]." Seems like the citation here should be to the originators of RFF (Rahimi and Recht, 2008).

• Bottom of page 14: "This means we no longer optimize in the domain X , instead we find φ_* in Rd that should correspond to the optimal point $x_* \in X$. We then decode φ_* by finding the point $x \in X$ that maximized $\varphi(x) \cdot \varphi_*$." Is there a guarantee that φ_* actually is the image of $x_* \in X$? Doesn't seem obvious and it would be good to explain why if so, and clarify if not. In general, it seems like $\{\varphi(x) : x \in X\}$ would be a sub-manifold of Rd. Are we guaranteed that φ_* lies on this sub-manifold?

References

Mark A Aizerman. Theoretical foundations of the potential function method in pattern recognition learning. Automation and remote control, 25:821–837, 1964.

Francis Bach. On the equivalence between kernel quadrature rules and random feature expansions. Journal of Machine Learning Research, 18(21):1–38, 2017.

Kenneth L Clarkson, Shashanka Ubaru, and Elizabeth Yang. Capacity analysis of vector symbolic architectures. arXiv preprint arXiv:2301.10352, 2023.

E Paxon Frady, Denis Kleyko, Christopher J Kymn, Bruno A Olshausen, and Friedrich T Sommer. Computing on functions using randomized vector representations. arXiv preprint arXiv:2109.03429, 2021.

Yarin Gal and Richard Turner. Improving the gaussian process sparse spectrum approximation by representing uncertainty in frequency inputs. In International Conference on Machine Learning, pages 655–664. PMLR, 2015.

James Hensman, Nicolas Durrande, and Arno Solin. Variational fourier features for gaussian processes. Journal of Machine Learning Research, 18(151):1–52, 2018.

Miguel L´azaro-Gredilla, Joaquin Quinonero-Candela, Carl Edward Rasmussen, and An´ıbal R Figueiras-Vidal. Sparse spectrum gaussian process regression. The Journal of Machine Learning Research, 11:1865–1881, 2010.

Zhu Li, Jean-Francois Ton, Dino Oglic, and Dino Sejdinovic. Towards a unified analysis of random Fourier features. arXiv preprint arXiv:1806.09178, 2018.

David Lopez-Paz, Suvrit Sra, Alex Smola, Zoubin Ghahramani, and Bernhard Sch¨olkopf. Randomized nonlinear component analysis. In International conference on machine learning, pages 1359–1367. PMLR, 2014.

Tony A Plate. Holographic reduced representation: Distributed representation for cognitive structures. 2003.

Maxim Raginsky and Svetlana Lazebnik. Locality-sensitive binary codes from shift-invariant kernels. Advances in neural information processing systems, 22, 2009.

Ali Rahimi and Benjamin Recht. Random features for large-scale kernel machines. In Advances in Neural Information Processing systems, pages 1177–1184, 2008.

Anthony Thomas, Sanjoy Dasgupta, and Tajana Rosing. A theoretical perspective on hyperdimensional computing. Journal of Artificial Intelligence Research, 72:215–249, 2021.

Martin J Wainwright. High-dimensional statistics: A non-asymptotic viewpoint, volume 48. Cambridge University Press, 2019.

(Remarks on code availability)

Version 1:

Reviewer comments:

Reviewer #2

(Remarks to the Author)
I would like to thank the authors for preparing this very comprehensive revision. The additional experiments on combinatorial optimization problems, the inclusion of new baselines, and, in particular, the demonstrated hardware deployment on two neuromorphic platforms make this work substantially complete. Therefore, I believe my initial concerns regarding novelty have been addressed.

Regarding practicality and motivation, the reiterated concept of representing both continuous and discrete spaces through the SSP kernel is indeed a key enabler. Moreover, the continuous VSA space facilitates energy-efficient sampling on neuromorphic hardware.

Although I am still missing a concrete example where the achieved speedup and energy efficiency lead to a significant improvement in an end-to-end application, I recommend this work for publication.

(Remarks on code availability)

Reviewer #3

(Remarks to the Author)
I am completely satisfied with the authors' responses to my previous comments.

With respect to the authors' responses to the other reviewers' comments, these have significantly helped my understanding of the sections that fall outside my primary area of expertise. I also appreciated the changes to the introduction to make it

more explicitly structured and by making conceptual points more explicit, rather than relying on the reader to have the background required to join the dots.

Ross W. Gayler

=====

The points below are some trivial copy-editing/typo-like points I picked up on reading the revised manuscript (referenced to the PDF version).

(1) Page 2, Footnote 2: referenced to ==> referred to

(2) Page 7, Figure 3, caption: solid blue line ==> dashed blue line [or, drop "dashed" because there is only one blue line]

(3) Page 7, Figure 3, caption: after five samples ==> after ten samples [assuming the label on panel b is correct]

(4) Page 8, Footnote 7: that to initialize ==> that are used to initialize

(5) Page 10, Figure 7, caption: solid lines show ==> lines show [only one of the lines in each panel is solid]

(6) Page 10, Table III, CPU row: 0.234 +/- 0.062 ==> (234 +/- 62) x 10^-3 [for easier comparison with the other rows]

(7) Page 11, Figure 8, caption: Solid lines show ==> The lines show [only one of the lines in each panel is solid]

(8) Page 14, Footnote 12: is not a probability ==> it is not a probability

(9) Page 16, Paragraph 1: language for compositing kernels ==> language for composing kernels

(10) Page 16, Paragraph 2: This compliments and expands ==> This complements and expands

(11) Page 20, Data Availability: Please drop the "upon request" and put all the data with a permissive licence (e.g. CC-BY) in a findable and permanent public repository. Every meta-science study I have seen that looks at "upon request" has found it to be woefully ineffective in practice. [Not a copy-edit, obviously, just a firmly held view about how things should be done.]

(12) Page 27, Table V, caption: all methods used methods used to ==> all methods used to

(13) Page 29, Figure 15, plot: Panels "Pest Control" and "RNA Inverse Folding" have been labelled as panels a and b, whereas they should be d and e

(14) Page 29, Figure 15, caption: p > 0.05 ==> p > 0.05 (0.01 <= p <= 0.05 is unmarked on the plots) [I think it's helpful to explicitly point out that that there is a range of p values that is not displayed on the plots as significant or non-significant]

(15) Page 29, Paragraph 3: scales best with increasing m ==> scales best with increasing m on this specific test function [Given the results of your earlier comparisons of Hex and Rand I doubt that Rand would always be better for similar tasks on different functions.]

(Remarks on code availability)

Reviewer #4

(Remarks to the Author)
See attached pdf.

(Remarks on code availability)

Version 2:

Reviewer comments:

Reviewer #2

(Remarks to the Author)
I thank the authors for the comprehensive responses and the substantially revised manuscript. I find the new framing of SSP-BO as a fixed-resource parametric model (explicitly motivated by constraints of neuromorphic and hardware deployment) to be effective and well justified.

That said, allocating sufficient resources (i.e., choosing the embedding dimensionality d) a priori remains a nontrivial challenge. Over-dimensioning can lead to unnecessary computational overhead, while under-dimensioning risks rapidly saturating capacity and degrading performance. The authors' empirical exploration of this trade-off (Fig. 17) is therefore particularly valuable, and the results suggest that SSP-BO degrades more gracefully than alternative embeddings as capacity becomes constrained.

Finally, I agree with the authors that the compositional structure inherent to VSA representations could offer additional functional benefits beyond those explored here. I see this as an exciting and well-motivated direction for future work.

(Remarks on code availability)
The first bullet point in the introduction (Scalability) has a type. I think it should be as in the abstract "our approach reduces this to O(d^2) and O(d^2), respectively".

Reviewer #3

(Remarks to the Author)
I am completely satisfied with the authors' responses to my previous comments.

Ross W. Gayler

(Remarks on code availability)

Open Access This Peer Review File is licensed under a Creative Commons Attribution 4.0 International License, which permits use, sharing, adaptation, distribution and reproduction in any medium or format, as long as you give appropriate credit to the original author(s) and the source, provide a link to the Creative Commons license, and indicate if changes were made.
In cases where reviewers are anonymous, credit should be given to 'Anonymous Referee' and the source.
The images or other third party material in this Peer Review File are included in the article's Creative Commons license, unless indicated otherwise in a credit line to the material. If material is not included in the article's Creative Commons license and your intended use is not permitted by statutory regulation or exceeds the permitted use, you will need to obtain permission directly from the copyright holder.
To view a copy of this license, visit https://creativecommons.org/licenses/by/4.0/

Authors' Response to Reviews of

Compositional Neurosymbolic Representations Enable Efficient Active Exploration

P. Michael Furlong, Nicole S.-Y. Dumont, Rika Antonova, Jeff Orchard, Chris Eliasmith
Nature Communications,

RC: ***Reviewers' Comment***, AR: Authors' Response, ☐ Manuscript Text

Dear Editor and Reviewers,

We would like to thank you for the time and effort you dedicated to reviewing our manuscript and for the constructive feedback. We have spent considerable time making substantial revisions to address the concerns raised and to improve the clarity, scope, and overall presentation of our work. We believe that the manuscript has been significantly strengthened as a result of this feedback. Below, we summarize the major changes and additions. Detailed, point-by-point responses to each reviewer (including explanations of how the listed changes address specific suggestions) are provided in the following sections. Reviewer 1, Reviewer 2, Reviewer 3, Reviewer 4.

- **New tasks and experiments:**
  - Added three new benchmark tasks to demonstrate SSP-BO's applicability across a variety of optimization domains, with comparisons to appropriate state-of-the-art methods. These include two combinatorial optimization problems and one graph optimization task. Results are presented in Sections II-C2 and II-C3, and in Figure 6, Table I, and Table II.
  - Conducted a new experiment analyzing performance as a function of action space size (Figure 17).
- **Neuromorphic hardware demonstration:** Developed a neural model for acquisition function optimization and demonstrated its performance on two neuromorphic hardware platforms (SpiNNaker and Loihi 2), making this the first neuromorphic implementation of BO (see the new Section III-C for discussion of related work). Performance on global optimization problems is reported for both platforms, along with power usage of the models. These results are given in Section II-C5, Figure 9, and Table III.
- **Additional comparison baselines:**
  - Included a comparison to a Random Fourier Feature (RFF)-based GP approximation on global optimization tasks (Figure 5).
  - Added a sparse GP baseline for the robotics tasks on the request of Reviewer 2.
  - The new benchmarks include new baseline algorithms. For the combinatorial tasks, these are COMBO [187], BOiLS [102], BODi [59], and BOSS [174]. For the graph task, baselines include NASBOT [122], NAO [165], BANANAS [259], BONAS [222], AG-Net [164], and DiNAS [12].
- **Streamlined methods:** Aspects of our approach were simplified for consistency and to allow seamlessly integration into existing systems.
  - Replaced the Mutual Information-based acquisition function with the more commonly used Upper Confidence Bound (UCB) to ensure consistency with baseline agents in the robotics tasks. All experiments were re-run using this acquisition strategy.

- Replaced the decoder network with a simpler similarity-based decoding method and removed decoder runtime comparisons accordingly. Although this slightly increases sample selection time, it simplifies the methods and offsets the increased word count from other additions.
- Removed β decay in the acquisition function for the PSR task to maintain consistency with other tasks and baselines.

- **Modified tasks:** The SSP size used for the global optimization benchmarks was changed from 151 to 97. The robotics tasks were updated for realism in several ways. Note that the changes to the objective functions mean that the reward values in the current manuscript (Figure 7) are not directly comparable to the values from the previous draft.
  - Added energy costs to the Box Reach and Belt Stretch tasks for realism and consistency with the PSR task.
  - Changed the energy cost term in the PSR objective from a divisive to a subtractive form, allowing high-rewarding solutions to more directly correspond to locations of maximum subsurface water density.
  - Updated the PSR objective to use the *maximum* subsurface water density over trajectories (instead of the average). This better reflects the realistic use case of guiding a drilling robot, as such a robot that would drill at a single location (the maximum over trajectories) rather than drilling along a trajectory.
- **Improved BoTorch baseline:**
  - BoTorch now refits the length-scale parameters online.
  - BoTorch now runs on a GPU (H100). Previously, a CPU was used to be consistent with SSP-BO. The GPU results have been added to the timing plots (Figure 8) and Table II to make the baseline timing more competitive.
- **Significant restructuring, rewriting, and review expansion:** In response to reviewer feedback, we significantly expanded prior work, improved the flow and organization of the manuscript, and highlight our contributions more clearly.
  - Rewrote the Introduction section and added new subsections to more thoroughly cover prior work on kernel methods and approximations, related work using VSAs, and to better highlight the main contributions of our work.
  - Added a new figure (Figure 2) to visualize the different action spaces tested, emphasizing the main contribution of the paper: that VSA embeddings enable efficient BO in diverse domains.
  - Expanded comparison to prior work in the new Discussion section (Section III-A).
  - Reorganized the Results sections to present the VSA formalism, embedding schemes, and SSP-BO algorithm earlier.
  - Moved the task overview figure (Figure 4) earlier in the manuscript to better orient the reader.
  - Added a new Methods subsection (Section V-D) discussing the representational capacity and scaling properties of VSAs.

1. Reviewer #1

RC: ***The proposed method involves a sophisticated integration of compositional neurosymbolic representations and Bayesian optimization. This complexity might make it challenging to implement and understand for practitioners who are not familiar with neurosymbolic programming and Vector Symbolic Algebras (VSAs).***

AR: We recognize the complexity involved in integrating VSAs and Bayesian optimization. To aid understanding, we have expanded and reorganized the background material. Specifically, we have added more background and references to prior work using VSAs in the Introduction to better contextualize our approach. We have also attempted to draw the connection more clearly between VSAs and kernel embeddings, which should be more familiar to a broader audience. Additionally, the *Vector Symbolic Algebra (VSA)* subsection has been moved earlier in the manuscript, from Methods to Results, to improve clarity. To further support practitioners, we will release the experiment code and a Python package upon publication to facilitate adoption of our method.

RC: ***The comparison with state-of-the-art methods like BoTorch is promising, but a more detailed comparative analysis with a broader range of existing techniques in Bayesian optimization and reinforcement learning could provide a clearer picture of the advantages and limitations of SSP-BO. This includes performance metrics, resource consumption, and applicability to different types of problems.***

AR: We have both significantly extended our experimental evaluation to include a broader range of problem types and comparison methods, and have considered other methods. First, we introduce two combinatorial optimization tasks: a synthetic pest control task [187] and an RNA inverse folding task [169]. We compare SSP-BO with several GP-based Bayesian optimization methods, including COMBO [187], BOiLS [102], BODi [59], and BOSS [174]. These use different kernels and optimization algorithms tailored to combinatorial variables.

These new results are presented in the Section II-C2 *VSAs Enable Fast BO for Combinatorial Tasks*, which states:

> Within a budget of $N = 200$, our approach achieves a best objective value within the 95% confidence intervals of the top-performing BO methods in Dreczkowski et al. [30], demonstrating competitive performance while substantially reducing computation time. The total time spent by SSP-BO planning actions and updating its surrogate model is 205 to 3891 times faster than the total time for the other BO methods on the pest control task and 176 to 2763 times faster on the RNA inverse folding task.

Second, we evaluate SSP-BO on NAS-Bench-101 [275], a graph-based benchmark for Neural Architecture Search (NAS), comparing our method with NAO [165], BANANAS [259], BONAS [222], AG-Net [164], and DiNAS [12]. We find that SSP-BO performs comparatively to BO-based NAS methods and slightly worse than state-of-the-art non-BO NAS methods. Results are reported in the new section Section II-C3 *VSAs Enable Optimization over Graphs*.

Additionally, we expanded the baseline comparisons on existing tasks: on the global optimization benchmarks we included RFF-BO, a Random Fourier Feature-based GP approximation adapted from [192], and on the robotics tasks we have added a sparse variational GP model [111] with a fixed number of inducing points, initialized following [31]. These baselines were added on the request of other reviewers.

We carefully considered several classes of other reinforcement learning (RL) methods as potential baselines. Modern model-free RL achieved notable breakthroughs. However, leading methods such as PPO [216]

and SAC [103] need one-to-two orders of magnitude more environment interaction steps than our method. Model-based methods can be more data-efficient, but scalable versions require much more memory. For example, [38] outperformed a number of model-based and model-free methods in data-efficiency, but even for simpler problems it required an order of magnitude more memory (even for a single expert in its model ensemble) than our method. Hence, we compared our method to scalable versions of active learning, e.g. with sparse GPs, since these are known to be both memory- and data-efficient. Nonetheless, our approach can help GP-based RL methods, such as [54], to leverage compositionality and scale up more effectively in the future. We included this initial discussion in Section G (*Neuroscience and cognition*) of the supplementary material.

Overall, the new results are used to demonstrate the advantages and limitations of SSP-BO: it can be applied to a diverse set of problem types, is highly efficient in terms of time and memory, can be deployed on neuromorphic hardware (and thus be useful for energy-efficient optimization in edge computing applications), but it is not the top-performing optimizer on *all* problems.

RC: ***The paper acknowledges the current inability to handle concept drift and change hyperparameters online. This limitation can be significant in real-world applications where environmental conditions and system dynamics frequently change, necessitating adaptive learning capabilities.***

AR: We discuss this limitation in Section III-D *Limitations and Areas for Improvement*. While SSP-BO currently assumes stationary kernels, we acknowledge the importance of adapting to concept drift and online hyperparameter tuning. Promising directions for future work include leveraging non-stationary SSPs [64] or recursive SSPs [254], which would allow SSP-BO to adapt dynamically. However, we consider this to be a significant extension and believe it merits dedicated investigation in a future publication.

RC: ***The decoding process, which converts the optimal vector back to the domain space, relies on pre-trained neural networks. This introduces additional complexity and potential sources of error, especially when the length scales change. The need to retrain the network for different scales can be seen as a limitation in dynamic environments.***

AR: Due to this concern and the increased word count from the new experiments and baselines, we removed the decoder networks and now employ a standard similarity-based decoding approach, which is simpler and widely used in VSA literature (see the revised Section V-C *Feature Decoding* section). This method is robust, but slower than the decoding network. However, all timing results include decoding time and we still see significant improvements over all baseline methods. While recent work has proposed more advanced decoding techniques [26, 40, 148], decoding is not the focus of this paper.

RC: ***The paper briefly discusses the trade-offs in approximation accuracy. The reliance on VSAs and the induced kernels might introduce approximation errors that can affect the overall performance, especially in highly sensitive applications.***

AR: SSP-BO approximates Gaussian Processes and therefore introduces some approximation error. However, our experimental results show that it performs on par with, or even better than, exact GP-based methods across diverse tasks. To address this issue more thoroughly, we have added a new section titled Section V-D *Representational Capacity and Scaling Properties*, which discusses existing theoretical results on the approximation capacity of VSA encodings. Additionally, we conducted a new experiment to examine how performance scales with dataset size, and observed slightly better scaling behaviour compared to the GP and RFF baselines (see Section D *Dimensionality Experiments* in the supplementary material).

RC: ***The practical integration of SSP-BO with existing autonomous systems and frameworks is not extensively covered in the manuscript. Understanding how this method can be seamlessly integrated into current robotics and AI pipelines would be beneficial for wider adoption.***

AR: We have streamlined aspects of our methods so that SSP-BO can be easily integrated into various frameworks (see the *Streamlined methods* bullet point on the first page of this response document). In addition, we will release the SSP-BO codebase along with a user-friendly Python package to facilitate integration into existing systems. SSP-BO is designed to match the interface of standard BO frameworks, such as BoTorch, making it a drop-in replacement. As BO methods are already commonly used in robotics and AI pipelines, this compatibility ensures ease of adoption.

RC: ***No comparison with hardware-based designs (ASIC, FPGA, in-memory) is provided.***

AR: We have added experiments demonstrating SSP-BO's sample selection on two neuromorphic platforms (SpiNNaker and Loihi 2) for the global optimization benchmarks, making this the first neuromorphic implementation of BO. These results, presented in Section II-C5, demonstrate SSP-BO's suitability for deployment on low-power hardware. We have not been able to find literature on BO methods designed specifically for ASIC or FPGA platforms, but we have found examples of GP inferences being speed-up with specialized hardware. This work is discussed in a new section in the discussion, Section III-C *Relation to Past Work using Efficient Hardware for BO*:

> This is the first work to implement part of a BO loop – sample selection – on neuromorphic hardware. Previous efforts like LavaBO [139, 140] implement BO-compatible GPs, but only on CPUs, with future neuromorphic deploy- ment intended. Likewise, earlier work on SSP-BO presented Neuromorphic-compatible optimization but was tested on a CPU [48]. In contrast, here we test our method on both SpiNNaker and Loihi 2.
>
> Several efforts have explored accelerating GP inference using other specialized hardware. In particular, Cholesky decomposition and matrix-vector operations have been im- plemented on FPGA [29, 144] and ASIC [26] platforms. However, these works focus on low-level GP inference rather than addressing the overall scaling limitations of GP-based BO or its application to high-dimensional action spaces.

As we do not have access to this hardware, we are unable to use their methods in a BO loop applied to our tasks, and thus are unable to make direct comparisons. However, this past work has a different focus than our method. They use hardware to speed up the matrix inversion step of GP inference but do not address the problem of cubic scaling, nor do they explore approximate GP approaches.

RC: ***The authors only discuss reduction in computation time as the important benefit from their method. How about power and energy consumption?***

AR: This is an excellent point – especially because power savings are a major promise of research in neuromorphic computing. We have decided to demonstrate the sample selection step of SSP-BO and energy reduction on two neuromorphic chips to address your feedback. In addition to reduced computation time, we now report the energy use of our neuromorphic implementations of SSP-BO. These results show that using neuromorphic hardware yields an improvement in energy efficiency by a factor of 34 (see Table III). This demonstrates that SSP-BO can be deployed on low-power hardware, supporting its use in resource-constrained autonomous systems.

RC: ***The results are primarily based on simulated environments. Validating SSP-BO in real-world settings with physical robots and actual sensors would provide stronger evidence of its practical applicability and effectiveness.***

AR: We agree with the reviewer that real-world validation would further strengthen the work. However, BO is a very well-established tool, integrated into many physical robotic pipelines. What is missing from the literature is exploration of BO on neuromorphic platforms. Thus, for this revision, we prioritized demonstrating the

method's hardware efficiency through neuromorphic deployment. We view real-world neuromorphic robotic experiments as an interesting direction for future work.

RC: ***It surprises me that "Section II. Results" appears right after the introduction section. Is this a requirement for this journal? Otherwise, it is expected from the methodology is discussed and then we see the results. The paper organization can be improved.***

AR: Yes, the current structure follows the Nature Communications format, which requires sections in the following order: Introduction, Results, Discussion, and Methods. We agree that for a theoretically oriented paper, a traditional layout with Methods before Results would aid comprehension. To address this, we have reorganized and moved several key methodological elements into the Results section, treating them as core contributions. We also added a *Structure of the Paper* subsection (Section I-D) in the Introduction to help guide the reader.

RC: ***The baseline framework in this work is BoTorch. While reference [4] is provided on page 15 for BoTorch, its discussion starts way earlier in the paper. The reference should appear sooner.***

AR: Thank you for noting this. The citation for BoTorch now appears at the first relevant mention earlier in the manuscript.

RC: ***But why BoTorch is selected as the baseline in this work? More justification is needed to accept BoTorch is the state-of-the-art framework with best performance in this area.***

AR: We selected BoTorch as a baseline due to its wide adoption, scalability, and versatility across BO tasks. It serves as the foundation for many modern BO pipelines and is actively maintained. We have now included additional references supporting BoTorch's status as a state-of-the-art framework.

> For the robotics experiments, we use a state-of-the-art Bayesian optimization library called BoTorch [6]. BoTorch offers a variety of options for Gaussian processes (built on top of GPyTorch [50]) and their kernels. Additionally, it al- lows for kernel hyperparameters to be fit automatically using marginal likelihood, as described in Section V-A in [132]. Many recent BO methods are built on top of BoTorch and use the standard library as a baseline [2, 102, 13, 28].

See `https://botorch.org/docs/papers/` for more examples of recent papers using BoTorch.

Furthermore, we have added other state-of-the-art BO methods for comparison. On the combinatorial optimization tasks we compare SSP-BO with several GP-based Bayesian optimization methods, including COMBO [187], BOiLS [102], BODi [59], and BOSS [174]. These use different kernels and optimization algorithms that operate on combinatorial variable spaces, which is why they were not used for the trajectory space tasks. For the graph task (NAS-Bench-101), we compare our method with NAO [165], BANANAS [259], BONAS [222], AG-Net [164], and DiNAS [12]. Again, these methods are for graph optimization and are not directly applicable to the robotics tasks.

Additionally, we expanded the baseline comparisons on existing tasks: on the global optimization benchmarks we included RFF-BO, a Random Fourier Feature-based GP approximation adapted from [192], and on the robotics tasks we have added a sparse variational GP model [111] with a fixed number of inducing points, initialized following [31]. These baselines were added on the request of other reviewers.

RC: ***Table I appears in page 4 but it's cross-referenced for the first time in page 5.***

AR: We have corrected this.

References

[12] Rohan Asthana et al. "Multi-conditioned graph diffusion for neural architecture search". In: *arXiv preprint arXiv:2403.06020* (2024).

[26] Alicia Bremer and Jeff Orchard. "Improved Cleanup and Decoding of Fractional Power Encodings". In: *Proc. Neuro-Inspired Computational Elements*. 2025.

[31] David R Burt, Carl Edward Rasmussen, and Mark Van Der Wilk. "Convergence of sparse variational inference in Gaussian processes regression". In: *Journal of Machine Learning Research* 21.131 (2020), pp. 1–63.

[38] Kurtland Chua et al. "Deep Reinforcement Learning in a Handful of Trials using Probabilistic Dynamics Models". In: *Advances in Neural Information Processing Systems*. 2018.

[40] Kenneth L Clarkson, Shashanka Ubaru, and Elizabeth Yang. "Capacity analysis of vector symbolic architectures". In: *arXiv preprint arXiv:2301.10352* (2023).

[54] Marc Deisenroth and Carl E Rasmussen. "PILCO: A model-based and data-efficient approach to policy search". In: *Proceedings of the 28th International Conference on machine learning (ICML-11)*. 2011, pp. 465–472.

[59] Aryan Deshwal et al. "Bayesian optimization over high-dimensional combinatorial spaces via dictionary-based embeddings". In: *International Conference on Artificial Intelligence and Statistics*. PMLR. 2023, pp. 7021–7039.

[64] Nicole Sandra-Yaffa Dumont. "Symbols, Dynamics, and Maps: A Neurosymbolic Approach to Spatial Cognition". PhD Thesis. Waterloo, ON: University of Waterloo, 2025.

[102] Antoine Grosnit et al. "Boils: Bayesian optimisation for logic synthesis". In: *2022 Design, Automation & Test in Europe Conference & Exhibition (DATE)*. IEEE. 2022, pp. 1193–1196.

[103] Tuomas Haarnoja et al. "Soft actor-critic algorithms and applications". In: *arXiv preprint arXiv:1812.05905* (2018).

[111] James Hensman, Nicolo Fusi, and Neil D Lawrence. "Gaussian processes for big data". In: *arXiv preprint arXiv:1309.6835* (2013).

[148] Christopher J Kymn et al. "Computing with residue numbers in high-dimensional representation". In: *Neural Computation* 37.1 (2024), pp. 1–37.

[164] Jovita Lukasik, Steffen Jung, and Margret Keuper. "Learning where to look–generative nas is surprisingly efficient". In: *European Conference on Computer Vision*. Springer. 2022, pp. 257–273.

[165] Renqian Luo et al. "Neural architecture optimization". In: *Advances in neural information processing systems* 31 (2018).

[169] Nono SC Merleau and Matteo Smerlak. "A simple evolutionary algorithm guided by local mutations for an efficient RNA design". In: *Proceedings of the genetic and evolutionary computation conference*. 2021, pp. 1027–1034.

[174] Henry Moss et al. "Boss: Bayesian optimization over string spaces". In: *Advances in neural information processing systems* 33 (2020), pp. 15476–15486.

[187] Changyong Oh et al. "Combinatorial bayesian optimization using the graph cartesian product". In: *Advances in Neural Information Processing Systems* 32 (2019).

[192] Michael Osthege and Kobi Felton. *michaelosthege/pyrff: v2.0.3*. Version v2.0.3. Dec. 2020. DOI: 10.5281/zenodo.15048204. URL: https://doi.org/10.5281/zenodo.15048204.

[216] John Schulman et al. "Proximal policy optimization algorithms". In: *arXiv preprint arXiv:1707.06347* (2017).

[222] Han Shi et al. "Bridging the gap between sample-based and one-shot neural architecture search with bonas". In: *Advances in Neural Information Processing Systems* 33 (2020), pp. 1808–1819.

[254] Aaron R Voelker et al. "Simulating and predicting dynamical systems with spatial semantic pointers". In: *Neural Computation* 33.8 (2021), pp. 2033–2067.

[259] Colin White, Willie Neiswanger, and Yash Savani. "Bananas: Bayesian optimization with neural architectures for neural architecture search". In: *Proceedings of the AAAI conference on artificial intelligence*. Vol. 35. 12. 2021, pp. 10293–10301.

[275] Chris Ying et al. "Nas-bench-101: Towards reproducible neural architecture search". In: *International conference on machine learning*. PMLR. 2019, pp. 7105–7114.

2. Reviewer #2

RC: ***This paper proposes a spatial semantic pointer (SSP) based Bayesian optimization (BO) framework that reduces Gaussian process BO's time and memory complexity while maintaining a high reward performance. The key ingredient is to use fractional power encoding (FPE) with hexagonal grid representation, yielding a novel inner-product kernel that can be used with Bayesian linear regression for optimization. Experiments are conducted on synthetic test functions as well as multi-agent robotic simulations. In general, the paper is very well written, and the application of vector symbolic architectures (VSAs) seems to be meaningful in BO. However, I have some concerns that may be answered in a revised version of the manuscript:***

AR: Thank you for the constructive feedback. We hope we have addressed these concerns.

RC: ***Novelty***

The key concept of using VSA for BO has already been proposed in [39]. This manuscript mainly explores an alternative hexagonal kernel [23] (instead of a random one) to encode multiple feature dimensions. Apart from additional robotic benchmarks that required multi-trajectory exploration, this manuscript does not seem to introduce a sufficiently novel concept. It would be helpful if the authors could explicitly state the novelties introduced in this paper, given the already impactful work in [23] and [39].

AR: Indeed, [23] and [39] represent our earlier work, published respectively in a conference and workshop setting. As noted in Nature Communications' guidelines, submissions based on prior conference versions are welcome provided they include extensions. Our manuscript meets this criterion as there are several major contributions over past work:

- **Generalization Across Structured Spaces:** While earlier work served as a proof of concept for using SSPs in BO on small-scale synthetic problems, the current manuscript demonstrates the real strength of SSP-BO – its ability to naturally operate over a wide variety of structured domains due to the algebra of VSAs. In the first draft, we show that the method is effective on:
  - Continuous action spaces (also covered in our prior work);
  - Multi-agent trajectory spaces (a new addition),

  To further demonstrate the versatility of VSAs, we have significantly extended our experimental evaluation to show that SSP-BO can also be applied to:
  - Combinatorial optimization problems (*e.g.*, pest control [187], RNA inverse folding [169]). These new results are presented in the new section *VSAs Enable Fast BO for Combinatorial Tasks*.
  - Graph-structured optimization problems (*e.g.*, NAS-Bench-101 [275]). Results are reported in the new section *VSAs Enable Optimization over Graphs*.

  These new action spaces and the multi-agent trajectory domains are substantially more complex and diverse than those in our prior work, clearly demonstrating novel capabilities of our framework.
- **Extended Experimental Evaluation and Baselines:** We have expanded the number of methods we compare against. See our response to the comment ' Missing comparison to efficient SOTA BO.'
- **Neuromorphic Hardware Implementation:** We have implemented SSP-BO on two different neuromorphic computing platforms (SpiNNaker and Loihi 2), making this, to our knowledge, the first neuromorphic realization of BO. This illustrates the method's suitability for real-time, energy-efficient optimization tasks and highlights its potential in embedded and low-power AI applications.

We believe that these enhancements, along with our expanded theoretical discussion, constitute a substantial advance over previous work.

RC: ***Practical applications/motivation***

The introduction motivates Bayesian optimization (BO) for reinforcement learning and optimizing hyperparameters of machine learning algorithms. Reducing BO's complexity at iso-accuracy is obviously a useful thing to do; however, the paper does not explicitly state where the presented approach revolutionizes intelligent systems. Hence, it would be helpful to propose/show applications where SSP-BO can solve a problem that has not been possible with the previous methods (e.g., on specific hardware). In hyperparameter search for ML models, SSP-BO's advantage might not be significant as the bottleneck in hyperparameter search will instead be in the environment evaluation (i.e., the ML model training). Moreover, the memory requirements of SSP-BO are higher than BOTorch in practical domains: experiments on PSR (24D) reach convergence after n=100 samples (Fig. 3c), whereby the SSP-BO exceeds the memory requirements for n<187.

AR: Here we list the main contributions – the ways in which SSP-BO revolutionizes intelligent systems – and reference parts of the new manuscript where these contributions are highlighted.

- **Structured Search Spaces with Unified Embedding and Optimization:** While it is true that other BO methods can be extended to work with combinatorial, mixed, and graph-structured spaces through custom kernel design, these often require significant task-specific adaptation. In contrast, SSP-BO offers a systematic and generalizable approach: the algebra of VSAs provides a principled way to construct high-dimensional embeddings for arbitrary structured action spaces. Crucially, SSP-BO performs optimization not in the original action space, but in the continuous convex embedding space defined by these representations. This allows the same optimization procedure – whether a conventional gradient-based algorithm or a recurrent spiking neural network implementing gradient ascent dynamics, as demonstrated in our neuromorphic implementation – to be applied uniformly across diverse tasks. Furthermore, the surrogate model used in SSP-BO has a consistent form and learning procedure across these spaces, regardless of the underlying action type. This uniformity is a key strength of our framework, as it makes neuromorphic implementation feasible, and is a major departure from prior methods that require handcrafted solutions for each domain. See Section I-C, Figure 2, and Section III-B in the manuscript.
- **Neuromorphic and Energy-Efficient Hardware:** We demonstrate SSP-BO running on neuromorphic hardware, making it the first BO method implemented on such systems. BO using exact GP would not be suitable to such neural implementation, as the model size (and thus the required neural resources) grows unbounded. Approximate or exact GP approaches that optimize in the data space are also not suitable, as one would need to define specific neural population codes and recurrent dynamics for optimization based on the data domain. While in SSP-BO, all data types are represented by high dimensional vectors optimized with the same equation, making neural implementation straightforward. This opens up new applications in low-power, real-time systems (embedded autonomous agents, edge computing, and field robotics) where traditional GP-based BO is not feasible. This is particularly relevant given this special issue's focus on neural computation. SSP-BO provides a way to program neuromorphic hardware to exhibit the kinds of exploratory behaviours we may desire out of neuromorphic systems. See Section I-C, Section V-E and Supplementary Section G in the manuscript.

Regarding applications like hyperparameter or network architecture search, it is true that objective function evaluation often dominates total time cost. However, this is not true for all problems. Additionally, SSP-BO

has approximately a 200$\times$ improvement in total sample selection time compared to state-of-the-art BO methods in some cases, which is significant.

With the changes made to the objective functions, the settings of the baseline approach, and our algorithm (see the first page of this document), the results on robotics tasks have changed significantly. For example, the added energy costs make the robotics tasks more challenging. We have increased the budget of these tasks from $N = 150$ to 700, as there is still improvement at higher numbers of samples. Note that the online hyperparameter optimization now used with BoTorch allows it to further improve. This changes the memory comparison (see Figure 9 and 10) and convergence is reached at a point at which SSP-BO is more efficiency.

Additionally, we point out that the memory asymptotics of our representation are preferable to the standard approach. It is the asymptotics of this method that make it compatible with neural implementation. By maintaining a fixed dimensionality, we do not place additional burdens of neuron genesis in action selection neural circuits. It also means, modulo the points outlined in Section V-D *Representational Capacity and Scaling Properties*, that the resource requirements for the algorithm can be well characterized before deployment. This permits intelligent systems, especially those with compute resource constraints, to operate in the long-term with bounded resources.

RC: ***Missing comparison to efficient SOTA BO***

The results mainly compare SSP-BO with vanilla BO, whereby optimized versions are ignored (e.g., Sparse GPs or discretized state spaces mentioned in the introduction). It would be fair to include these methods in the experiments.

AR: We have added a sparse GP method for the robotics tasks:

> We compare against both exact GP inference – using BoTorch to compute Gaussian posteriors – and SVGP, a sparse variational GP model [65] with a fixed number of inducing points, initialized following Burt et al. [15]. The number of inducing points is chosen to match the memory usage of SSP-BO

See page 19 of the document.

Additionally, we have included RFF-BO, a Random Fourier Feature-based GP approximation, in the global optimization benchmark results. For the new combinatorial optimization problems, we compare SSP-BO with several GP-based Bayesian optimization methods, including COMBO [187], BOiLS [102], BODi [59], and BOSS [174]. For NAS-Bench-101 [275], the graph optimization benchmark, we compare SSP-BO with NAO [165], BANANAS [259], BONAS [222], AG-Net [164], and DiNAS [12].

RC: ***Other questions/remarks***

Is there a specific reason to use the sinc-kernel (i.e., uniform phase distribution) for the base vectors? Could you consider other alternatives kernels (shown in [34])?

AR: In most of our experiments, we do not use the standard Random SSPs (RandSSPs) with uniformly distributed phases – which in the limit $d \to \infty$ yields a sinc-shaped kernel – but instead use HexSSPs, which are constructed with carefully designed phase patterns based on a hexagonal lattice. These are not based on a uniform phase distribution and result in a non-sinc kernel.

That said, the underlying kernel shape in SSPs can indeed be modified by changing the phase sample distribution [80]. For instance, sampling phases from a normal distribution rather than a uniform one results in Gaussian kernel in the limit $d \to \infty$. However, since we are using finite dimensional embeddings, the kernel

retains (sinc-like) ripples regardless of the sampling distribution (as the kernel is a finite sum of cosines), particularly with a small size d as we use in this work.

We have explored both uniform and normal phase distributions in preliminary experiments and found that the choice of distribution had minimal impact on the performance of SSP-BO-Rand for the tasks we evaluated. Though it is true alternative kernel constructions are certainly possible and may offer advantages in some settings, they did not provide significant improvements in our domains of interest.

RC: ***I could not find a direct relationship/approximation between GP and the SSP kernel, as one knows typically from the RBF kernel. What exactly does the SSP kernel approximate? Is it simply a kernel that might work well in these scenarios with no direct relationship to a Gaussian process?***

AR: We have added the following to page 15 of the document to clarify:

> The dot product between SSPs is a kernel function, $k(\mathbf{x}, \mathbf{y}) = k(\mathbf{x} - \mathbf{y}) = \phi(\mathbf{x}) \cdot \phi(\mathbf{y})$. In general, the kernel's shape is dependent on the sampling distribution of the phase matrix components, $\mathbf{a}_j$ – in particular, it resembles the function whose power spectral density is the sampling distribution [126,152]. Suppose that the columns of the phase matrix are independent and identically distributed, sampled from a distribution P with probability density function $p(\mathbf{a})$, then the expected kernel is
>
> $$\mathbb{E}_{A\sim P}\left[\phi(\mathbf{x}) \cdot \phi(\mathbf{x} + \Delta\mathbf{x})\right] = \mathbb{E}_{A\sim P}\left[\frac{1}{d} e^{iA\mathbf{x}/\ell} \cdot e^{iA(\mathbf{x}+\Delta\mathbf{x})/\ell}\right], \tag{1}$$
>
> $$= \mathbb{E}_{A\sim P}\left[\frac{1}{d}\sum_{j=1}^{d} e^{i\mathbf{a}_j^T \Delta\mathbf{x}/\ell}\right], \tag{2}$$
>
> $$= \int_{\mathbb{R}^n} e^{i\mathbf{a}^T \Delta\mathbf{x}/\ell} p(\mathbf{a}) d\mathbf{a}, \tag{3}$$
>
> obtained using Parseval's theorem and the law of the unconscious statistician. This is the inverse Fourier transform of the distribution $p(\mathbf{a})$ – *i.e.*, its characteristic function.

See page 15.

The SSP kernel induces a band-limited kernel and what it approximates depends on $p(\mathbf{a})$ (just like RFFs). In the case of HexSSPs, the phases are constructed rather than sampled. The HexSSP kernel is characterized by directionally symmetric interference patterns that reflect the geometry of the underlying grid-like representation.

Also, note that while GPs are most commonly associated with the RBF kernel, they are, of course, compatible with any positive-definite kernel function. Although less common, band-limited kernels such as those derived from sinc functions have been explored in the GP literature – for example, in [242].

The kernel becomes more complicated once we introduce structure into the representations, which is the main benefit of the VSA approach over vanilla RFFs. The kernel that is induced by the dot product is an additive kernel of sub-components. Given a data structure, defined by $x = (\mathbf{x}_1, \mathbf{x}_2, \ldots, \mathbf{x}_n)$, and a vector embedding of the data structure, $\phi(x) = \sum_{i=1}^{n} \mathbf{g}_i \otimes \phi_i(\mathbf{x}_i)$, where $\mathbf{g}_i$ is a randomly generated "guard" vector (as discussed in Section V-B4), then the induced kernel is approximately:

$$\phi(x) \cdot \phi(x') \approx \sum_{i}^{n} k_i(\mathbf{x}_i, \mathbf{x'}_i).$$

RC: ***It would be helpful to describe m_n and Σ_n, used in Eq. (2) and Eq. (3), after or before the equations.***

AR: All terms in those equations are now explained in Section I-B *SSP-BO algorithm*.

> The parameters of this distribution are computed using standard BLR formulas [10, §3.3]:
>
> $$\mu_n(\mathbf{x}) = \mathbf{m}_{n-1}^T \phi(\mathbf{x}), \tag{4}$$
> $$\sigma_n^2(\mathbf{x}) = 1\beta + \phi(\mathbf{x})^T \Sigma_{n-1} \phi(\mathbf{x}), \tag{5}$$
>
> where $\mu_n(\mathbf{x})$ is the posterior mean of the predicted reward at $\mathbf{x}$, parameterized by the mean vector $\mathbf{m}_{n-1}$, and $\sigma_n^2(\mathbf{x})$ is the posterior variance, parameterized by the covariance matrix Σ_{n-1}. The precision parameter β is set as the inverse variance of the observed rewards and is estimated from the initial samples. The mean vector and covariance matrix are updated incrementally with each new observation.

RC: ***Counterintuitively, the total time in Table I is much shorter for more complex robotics applications compared to synthetic test functions. I guess this could be due to the different number of samples. It would be interesting to see how the method scales with the number of state space dimensionalities, e.g., by having the same number of samples across different tasks.***

AR: We have added such an experiment in the revised manuscript in Supplmentary Section D3 *Dimensionality Experiments*. We evaluate SSP-BO on the m-D Styblinski-Tang function, varying m to assess how performance scales with input size and embedding dimensionality d.

RC: ***I could not find any results in the robotic simulations using the SSP-BO-Rand. It would be good to show the superiority of SSP-BO-Hex by having this baseline, too.***

AR: We have added this to Figure 7. Note that the combinatorial tasks do not have results for SSP-BO-Rand (or SSP-BO-Hex), just SSP-BO. This is because these tasks do not include any continuous variables, and so the hexagonal versus random encoding is not relevant.

RC: ***Appendix E states that a neuromorphic computer would enable the advertised complexity of $\mathcal{O}(1)$; however, the current experiments were run on a traditional computer with $\mathcal{O}(d^2)$ (or $\mathcal{O}(d)$ when properly utilizing the GPU). It would also be good to make this assumption clear in the main text and abstract.***

AR: We agree and have removed that text to avoid confusion.

RC: ***The ablation in Fig. 11 shows that the vector dimensionality can be as low as d∼30 at iso-accuracy. This is quite surprising as VSAs typically require a dimensionality of hundreds or thousands. How can the kernel approximation still work in these low dimensions? Is it because we do not have any kernel addition in this example?***

AR: It is indeed somewhat surprising that SSP-BO performs well with relatively low-dimensional vectors (*e.g.*, $d = 30$), especially given that traditional VSAs (particularly binary VSAs) often require thousands of dimensions for reliable performance. There are several reasons why SSP-BO is effective even at these lower dimensionalities:

- **Type of VSA:** Our method uses the real-valued HRR VSA (equivalently, one could use the complex-valued FHRR VSA), which tend to be more compact and information-efficient than binary VSAs. These continuous representations allow useful kernel approximations even in lower-dimensional spaces.

- **Smoothness of the Objective Function:** The functions optimized in our tasks tend to have relatively smooth and low-frequency structures. In such cases, fewer basis functions (and thus lower dimensional embeddings) are sufficient to capture the relevant structure for optimization.
- **Decoding and Clean-up:** Even when the optimization process yields points that are far from valid embeddings on the VSA manifold, our similarity-based decoding acts as a form of projection or clean-up, guiding the selected vectors back toward interpretable or valid actions. This clean-up mechanism can compensate for inaccuracies due to reduced dimensionality.
- **Surrogate Model:** BLR is more forgiving of lower-dimensional representations compared to more expressive models like full GPs. In SSP-BO, the surrogate model operates directly on the VSA embeddings, and due to the relatively low sample complexity of the tasks, the model can learn accurate predictions with fewer degrees of freedom.

References

[12] Rohan Asthana et al. "Multi-conditioned graph diffusion for neural architecture search". In: *arXiv preprint arXiv:2403.06020* (2024).

[59] Aryan Deshwal et al. "Bayesian optimization over high-dimensional combinatorial spaces via dictionary-based embeddings". In: *International Conference on Artificial Intelligence and Statistics*. PMLR. 2023, pp. 7021–7039.

[80] E Paxon Frady et al. "Computing on functions using randomized vector representations". In: *arXiv preprint arXiv:2109.03429* (2021).

[102] Antoine Grosnit et al. "Boils: Bayesian optimisation for logic synthesis". In: *2022 Design, Automation & Test in Europe Conference & Exhibition (DATE)*. IEEE. 2022, pp. 1193–1196.

[164] Jovita Lukasik, Steffen Jung, and Margret Keuper. "Learning where to look–generative nas is surprisingly efficient". In: *European Conference on Computer Vision*. Springer. 2022, pp. 257–273.

[165] Renqian Luo et al. "Neural architecture optimization". In: *Advances in neural information processing systems* 31 (2018).

[169] Nono SC Merleau and Matteo Smerlak. "A simple evolutionary algorithm guided by local mutations for an efficient RNA design". In: *Proceedings of the genetic and evolutionary computation conference*. 2021, pp. 1027–1034.

[174] Henry Moss et al. "Boss: Bayesian optimization over string spaces". In: *Advances in neural information processing systems* 33 (2020), pp. 15476–15486.

[187] Changyong Oh et al. "Combinatorial bayesian optimization using the graph cartesian product". In: *Advances in Neural Information Processing Systems* 32 (2019).

[222] Han Shi et al. "Bridging the gap between sample-based and one-shot neural architecture search with bonas". In: *Advances in Neural Information Processing Systems* 33 (2020), pp. 1808–1819.

[242] Felipe Tobar. "Band-limited Gaussian processes: The sinc kernel". In: *Advances in Neural Information Processing Systems* 32 (2019).

[259] Colin White, Willie Neiswanger, and Yash Savani. "Bananas: Bayesian optimization with neural architectures for neural architecture search". In: *Proceedings of the AAAI conference on artificial intelligence*. Vol. 35. 12. 2021, pp. 10293–10301.

[275] Chris Ying et al. “Nas-bench-101: Towards reproducible neural architecture search”. In: *International conference on machine learning*. PMLR. 2019, pp. 7105–7114.

3. Reviewer #3

RC: ***Given that the primary purpose of this review is to provide feedback on the soundness of the research reported in order to help the authors to improve their manuscript and the editors to reach a decision, I am in a slightly odd position; I think the reported research is sound and there are no simple suggestions I could make for improving the manuscript. I could document that very briefly (which I will do below) and be done. However, I think this manuscript has hidden depths which I would like to discuss because it may assist the editors. (It may also be of interest to the authors, although I presume they are already more than aware of this depth.)***

AR: We are very grateful for your detailed and insightful review. Your engagement with the manuscript and comments are sincerely appreciated.

RC: ***- Development of a VSA representational scheme for compositional structured entities with discrete and continuous components able to be used in a Bayesian optimisation process and also compatible with neuromorphic implementation (spiking neuron neural networks). This is important because optimisation over mixed and discrete and continuous spaces can be problematic. (VSA avoids this by representing all structures in a continuous vector space.)***

AR: We are glad that you highlighted the compatibility with neuromorphic hardware. This was an important motivation for us – demonstrating that VSA-based optimization can be implemented not just in principle, but in a form usable in real-world systems with strict resource constraints. We believe this connection to energy-efficient, event-driven computation (such as with spiking neural networks) opens important new avenues for applying BO in autonomous agents and edge devices. To further emphasis this point we have added additional experiences demonstrating sample point selection on two neuromorphic platforms (SpiNNaker and Loihi 2).

RC: ***This manuscript can be read on multiple levels. The surface reading is that it is about a VSA-based system for Bayesian optimisation for active exploration. The deeper level reading is that it is about the utility, adaptability, and value of VSA as a basis for implementing computational systems. Because it's not a book-length manuscript it uses the tactic of providing a specific example (Bayesian optimisation for active exploration) and implicitly invites the reader to generalise beyond that example.***

The principal conceptual work component of this manuscript is a design process: How to design a VSA-based computational mechanism that achieves the computational objectives?

...

- The manuscript exposes much of the reasoning behind the design process. This is very valuable for readers trying to understand VSA and how they might apply it to their own problem domains. (Many papers are inclined to present the resulting mechanism without giving any insight into the design process.)

AR: This was one of our aims with this work: to demonstrate the utility and use of VSAs in a way accessible to a broader ML audience. We did this with the framing of VSAs as an efficient method for introducing compositional structure into kernel approximations. This framing is more heavily emphasized in the revised manuscript as per reviewers' feedback. In addition, by explaining the action embeddings (done earlier on in the revised document) and algorithmic design, we aim to equipped readers with the knowledge (and motivation) to adopt VSAs into their own work.

RC: ***- The manuscript goes to considerable lengths to make links to other fields, e.g. machine learning,***

psychology, cognitive science, robotics, neuromorphic computing, and neuroscience. The citations throughout the paper are wide ranging. I found the Appendix from Section D onwards to be very interesting in this respect.

AR: Thank you. Indeed, this speaks to a core motivation of the work. One of our goals was to position VSAs not just as an abstract computational tool but as a bridge between symbolic and neural representations, with relevance to the ML, robotics, and neuroscience communities systems. We are glad the broad citation base and interdisciplinary framing supported that aim.

4. Reviewer #4

RC: ***This has the potential to be an interesting paper, however it is poorly written, and does not frame its claims within the context of things that are already well-known and studied in the machine learning literature. Overall, the paper feels like a rush job, and I don't get the feeling the authors put much thought into how to communicate these ideas to a broad audience such as Nature Communications. For example the description of the BO problem being solved is extremely terse, and how these variables are being represented by high-d vectors will not at all be obvious to the reader not already acquainted with VSA. In addition the machinery of bundling and binding is buried in the appendix, not in the body of the paper, which would seem to take the wind out of the sails of the "compositionality" concept touted in the title and intro of the paper. In fact, how compositionality relates at all to the BO problem the authors are solving is entirely unclear just from reading the body of the paper.***

AR: Thank you for your candid and critical feedback. We have spent considerable time refining the manuscript after receiving reviewer feedback to improve both its clarity and its grounding in the broader literature and emphasize the main contributions.

We acknowledge your point that parts of the original presentation, especially the structure and exposition, made the work difficult to follow. This was in part due to the journal's format requirements, which place the Results section immediately after the Introduction and Methods after the Conclusion (this is not an appendix section, but its placement does make it appear to be one). While this works well for experimental papers, it can be suboptimal for theory-heavy work such as ours. As a result, some critical explanations (particularly of the VSA operations) were originally placed in the Methods, making the key ideas of compositionality harder to access from the main narrative.

We have now addressed this in several ways:

- **Reorganized Content:** Core methodological details – including the mechanisms of bundling, binding, and how discrete and continuous variables are embedded into high-dimensional spaces using VSAs – have been moved from Methods into the Results (in Section II-A *Vector Symbolic Algebra (VSA)* to Section II-A4 *Representing Trajectories and Sets*). These are a central part of our work and are now presented earlier in the manuscript.
- **Contextualization in the Introduction:** In the original manuscript, references to relevant past work were primarily in the Discussion and Methods sections. In the current version, we have rewritten the Introduction with several subsections to better situate the work within both the machine learning and VSA literature. This includes two new subsections: one reviewing prior work on kernel methods and kernel approximations in BO, and another on prior applications of VSAs. These help clarify how our work relates to existing approaches and what the compositional properties of VSAs bring to the BO setting.
- **Role of Compositionality:** We now more explicitly connect compositionality to the central challenge of optimization in structured and hybrid spaces (e.g., sequences, graphs, trajectories). We talk about prior approaches to this in Sections I-B *Compositional Kernels and Approximations* and III-A *Relation to Past Work using Kernels and Approximations*, and explain why this is an important problem. The VSA framework enables these composite structures to be embedded into a shared continuous space where optimization can proceed using a unified surrogate model and acquisition strategy. We have revised the text to highlight this clearly and earlier in the paper.

- **Addressed Terseness in BO Problem Setup:** We expanded our explanation of the BO problem, including how the action variables are represented using high-dimensional vectors (Section II-A1 *Representing Discrete Symbols* to Section II-A4 *Representing Trajectories and Sets*). Additional diagrams and clarifying examples have been added to help readers unfamiliar with VSA understand the intuition behind our encoding and optimization process.

RC: ***In terms of relating to the machine literature - the idea of approximating a kernel function using dot-products between random embeddings of modest dimension is widely studied and explored in the ML literature. There are by now dozens of papers on this topic in the context of kernel machines in general (Rahimi and Recht, 2008; Lopez-Paz et al., 2014; Li et al., 2018), and several which discuss this in the context of GP specifically (Lazaro-Gredilla et al., 2010; Gal and Turner, 2015; Hensman et al., 2018). So, I feel there is a need to more clearly flesh out the messaging of this paper to a broader audience: the general strategy being advocated here is, to a large extent, what people already would do.***

AR: We agree that this is a well-established area in the machine learning literature, and we have revised the manuscript to better situate our contributions in relation to this line of work. In particular, the re-written Introduction beginning and the new Section I-A, Section I-B, and Section III-A thoroughly discuss foundational and recent efforts, including those you cited (e.g., Rahimi and Recht, 2007; Gal and Turner, 2015; Hensman et al., 2018). The Introduction now clearly acknowledges that the strategy of using dot products between random embeddings to approximate kernel functions is a well-studied technique in both kernel machines and Gaussian process (GP) modelling.

In this section, we also highlight a gap in this field, which our work addresses:

> In machine learning, kernel methods offer a principled way to encode structured inductive biases [128], support data-efficient learning, and model uncertainty. Compositional kernels, constructed by combining simpler kernels via addition or multiplication, enable the expression of rich relational structures [36]. However, the practical application of such kernels is hindered by scalability: inference with Gaussian processes (GPs) requires $\mathcal{O}(n^3)$ time and $\mathcal{O}(n^2)$ memory, where n is the number of observations. While kernel approximation methods such as Random Fourier Features [RFFs; 124] reduce this complexity, approximating compositional kernels with RFFs often leads to unbounded growth in embedding dimensionality [106], and representing structured or symbolic inputs remains challenging.
>
> Vector Symbolic Algebras (VSAs) – specifically, Holographic Reduced Representations (HRRs) and their complex- valued variant, Fourier HRRs (FHRRs) [116, 118] – offer a promising solution by providing a dimensionality-preserving algebra for compositional representation. Vector embeddings used in kernel approximation – such as RFFs – typically implement composition using operations like concatenation (for additive kernels) or tensor products (for multiplicative kernels), both of which increase dimensionality.

In addition, comparison with previous work in the Discussion section has been expanded (Section III-A). Overall, we argue that we contribute the following:

- **Unified Algebra for Composition:** While many prior methods combine random feature embeddings through concatenation, tensor product, or learned neural embeddings, these approaches often suffer from unbounded growth in resource requirements as compositional complexity increases. In contrast, our method leverages a VSA, particularly HRR and FHRR, which enable structured, compressive, and

interpretable operations like binding and bundling. This allows for the construction of compositional kernels over structured and hybrid action spaces with fixed vector dimensionality (at the cost of approximation, which is discussed more later in this document).
Prior work on compositional kernels (e.g., Duvenaud et al., 2013; Daniely et al., 2017) often constructs compositional structure through kernel algebra (e.g., via sum and product), but lacks a unified representational system for encoding structured data directly into embeddings that preserve this compositionality. VSAs, by contrast, provide an explicit dimensionality-preserving algebra for the construction of representations and thus their induced kernels – offering a principled, interpretable, and systematic way to handle structured, discrete, and mixed-type data within a continuous optimization framework.

- **Closed-Form, Non-Learned Embeddings:** Unlike encoder-decoder architectures that require learning a mapping to and from continuous latent spaces (e.g., via autoencoders), VSA-based embeddings can be constructed directly via algebraic rules. This reduces computational overhead and improves interpretability.
- **Compatibility with Neuromorphic Implementation:** Our kernel representation is explicitly designed to support efficient Bayesian optimization in low-resource environments. By working within the VSA framework, we demonstrate a fully implemented BO algorithm on two neuromorphic platforms (SpiNNaker and Loihi 2), which to our knowledge is the first of its kind. This is primarily enabled by optimizing in the embedding space.

We hope that this revised framing and additional discussion help clarify both the connections to existing literature and the specific contributions our method makes to kernel-based BO. In essence, we do not dispute that the general idea of approximating kernels with random projections is well-known, but we argue that our work advances this line of research by 1) introducing a principled algebraic framework for constructing compositional kernels over hybrid and structured spaces, and 2) demonstrating the practical viability of this framework through implementation on neuromorphic hardware. Your feedback has helped us in clarifying this and we believe that it has improved the document.

RC: ***Moreover, the specific random embedding method used here is, as the authors state, closely related to random Fourier features, which is a particularly widely studied instance of the principle described above. However, this fact is only stated (and even then obliquely) on page (9) in the methods section. These connections should be stated bluntly and clearly early on: there is a large body of work in the broader machine learning community that applies very similar methods to achieve very similar ends vis-a-vis efficient realizations of kernel methods in general, and specifically, Gaussian Processes. Without this contextualization, it is difficult to understand why the framing in terms of VSA is helpful or should be of interest to the broader community. If the answer is item (2) above, then state this and let the reader decide how excited they are about it.***

AR: We agree and now mention Random Fourier Features (RFFs) for the first time in the Introduction on the first page, rather than in the Methods. The similarities and differences between our approach and standard RFF-based GP approximations are also discussed in both the Discussion and Methods sections (Section V-F). This helps clarify how our approach relates to widely known techniques in the GP literature.

We have also added a baseline comparison to an RFF-based Bayesian optimization method (RFF-BO) on global test functions, as well as a sparse GP method on the robotics tasks. These comparisons highlight where and how our approach differs in both performance and applicability.

We agree that computational efficiency (item 1) is a known and valuable feature of RFF-based approximations (which we show with the new RFF-BO baseline) and similar kernel approximation techniques. While our

method shares this benefit – and we believe it is important to highlight this in the paper given the special issue's theme of neuromorphic computing – we do agree that it is not the primary novelty of our work. Instead, the core contribution we aim to highlight is item (2): the integration of heterogeneous and structured inputs through the algebraic compositionality of VSA embeddings. This enables a unified optimization framework that operates across a variety of structured action spaces—continuous, discrete, mixed, graph-structured, and trajectories—without requiring custom kernel engineering or learned encoders for each task. The same optimization mechanism (and surrogate model) can be applied uniformly across domains.

To better emphasize this contribution, we have expanded the experimental evaluation to include additional task types – specifically, two combinatorial and one graph-based optimization problems – and added a new figure illustrating the induced kernel structures across different action spaces derived from VSA embeddings (Figure 2).

We believe that this contribution is of particular interest to the broader machine learning community because it offers a systematic and interpretable framework for constructing compositional kernels over structured data types. By enabling efficient optimization across diverse domains without the need for retraining, the method facilitates generalization while preserving the ability to capture structural relationships. This aligns well with growing interest in approaches that combine symbolic reasoning and continuous optimization, particularly in contexts where sample efficiency, flexibility, and interpretability are important.

RC: ***Abstract: "We apply our approach to Bayesian optimization in complex spaces, outperforming state-of-the-art algorithms...while reducing the time and memory complexity from O(n^3) and O(n^2) to O(1)." It would be more accurate to say "reducing time and memory complexity from O(n^3) and O(n^2) to O(d^3) and O(d^2)," where d is the hypervector dimension. Moreover, the statement as written is only accurate if d does not depend on n. It's not clear this is true: most of the available theory on RFF/VSA indicates that there is, in general, a dependence of d on n, or some other fundamental property of the input space. For results of this nature in RFF see (Rahimi and Recht, 2008; Bach, 2017). In the context of VSA see (Thomas et al., 2021; Clarkson et al., 2023). Reducing this to an O(1) complexity is a bit of an oversimplification.***

AR: We agree that the statement in the abstract was an oversimplification. The computational complexity of SSP-BO is more accurately described as reducing from O(n^3) time and O(n^2) memory complexity (for standard GP inference) to O(d^3) and O(d^2) (which O(1) in the number of samples). We have edited the abstract and the main text to state this.

> We apply our approach to Bayesian optimization in complex spaces, outperforming state-of-the-art algorithms on a number of learning and robotics tasks, while reducing the time and memory complexity from $O(n^3)$ and $O(n^2)$ to $O(1)$ in the number of samples.
>
> . . .
>
> We obtain constant-time sample selection with respect to the number of environment observations, n – reducing the computational complexity of standard BO from $\mathcal{O}(n^3)$ time and $\mathcal{O}(n^2)$ memory to $\mathcal{O}(d^3)$ and $\mathcal{O}(d^2)$, respectively, where d is the fixed size of the VSA embeddings. This parameter is set prior to optimization and is fixed over the course of exploration. Thus, the complexity of our algorithm is $\mathcal{O}(1)$ in the number of samples.

From page 1 and 2.

Moreover, it is true that the optimal d generally depends on properties of the data, including the size of the dataset n and the structure of the input space, as discussed in the literature on Random Fourier Features (RFFs) and Vector Symbolic Architectures. To address this issue more thoroughly, we have added a Section V-D

in the Methods discussing the representational capacity of VSA-based encodings, including the theoretical scaling relationships. This section cites the works you helpfully pointed out (e.g., [206, 13, 240, 40]) and others. We now clearly explain that:

- Although d must, in theory, scale with problem size or complexity to maintain high-quality approximations, it can be set once at deployment and remains constant during runtime.
- The required dimensionality d tends to scale sublinearly with n for smooth kernels and well-structured problems, and can often be surprisingly small (tens to low hundreds of dimensions) while maintaining high performance, especially with real- or complex-valued VSAs like HRR and FHRR.

We have also added a new experiment (described in the Supplementary Materials, Section D3) analyzing how regret changes as a function of data size m and embedding dimensionality d.

RC: ***Top of page 2: "From this we gain several benefits: 1) time and memory efficiency by transforming kernel machines to linear methods; 2) unified representations of mixed discrete and continuous spaces; and 3) an algebraic framework for understanding the content of neural activities." Re (1), see comment above w.r.t random feature models in general.***

AR: We have removed that sentence and now summarize the contributions in the Introduction as follows:

> This yields several advantages:
>
> - **Scalability:** We obtain constant-time sample selection with respect to the number of environment observations, n – reducing the computational complexity of standard BO from $\mathcal{O}(n^3)$ time and $\mathcal{O}(n^2)$ memory to $\mathcal{O}(d^3)$ and $\mathcal{O}(d^2)$, respectively, where d is the fixed size of the VSA embeddings. This parameter is set prior to optimization and is fixed over the course of exploration. Thus, the complexity of our algorithm is $\mathcal{O}(1)$ in the number of samples.
> - **Expressivity:** Our method supports structured, symbolic, and mixed discrete-continuous input spaces (*e.g.*, graphs, sets, trajectories) via a single algebraic framework.
> - **Neuromorphic compatibility:** Sample selection in SSP-BO occurs in the continuous embedding space. This makes it possible to perform sample selection over structured action spaces on neuromorphic hardware, providing a biologically grounded pathway for efficient decision-making systems.

We also state that it is the *combination* of the Scalability and Expressivity that is novel (page 2):

> Whereas existing approaches typically provide either scalable BO or expressive BO, this work combines both within a single compositional kernel embedding framework, enabling efficient optimization in structured domains and supporting neuromorphic implementation.

RC: ***Top of page 2: "Additionally, by operating in the high-dimensional vector space, using VSAs recasts the BO sample selection problem as a convex optimization problem, instead of an NP-hard optimization." This statement makes it sound like VSAs have managed to obtain a tractable solution to an NP hard problem. Rather, a more accurate description would be that the reward function is being approximated using a GP, which it is possible to estimate and sample from efficiently (e.g. in polynomial time). This idea, approximating an NP-hard objective by solving a tractable surrogate problem, is foundational to***

stat ML and doesn't have anything to do with VSA per-se. The step involving VSA is a second layer of approximation that uses the idea of RFF/random features to approximate a GP using Bayesian linear regression in a random feature space. See also the note below about convexity.

AR: As you correctly state, tractability is achieved by approximating the original (potentially NP-hard) optimization objective using a surrogate model, specifically a GP or its approximation via Bayesian linear regression on random feature embeddings. This general strategy – approximating an intractable problem with a more manageable surrogate – is indeed a foundational principle in many computational fields and is not unique to VSAs.

The role of VSAs in our approach is to provide a structured, fixed-size embedding framework that enables this without requiring learning or complex engineering for each new task. We have revised the text in the Introduction and Discussion to better reflect this distinction and removed any mention of NP-hard optimization. It now emphasizes that convexity and tractability arise from the surrogate modelling approach, and that VSAs provide the framework that enables efficient and unified application of this strategy across varied, structured spaces. For example, the Discussion now states (page 12):

> This design has several advantages. First, it yields interpretable embeddings: each component of the representation corresponds to a defined algebraic structure, not an opaque neural transformation. Second, unlike learned encoders, VSAs have established theory regarding their representational capacity (see Section V-D), offering stronger guarantees about generalization and expressivity. Moreover, many structured kernels – such as those used in graph and string domains [51, 109] – can be reformulated using VSAs. This formulation provides significant flexibility while keeping resource demands bounded. Because SSP-BO operates entirely in the embedding space, it avoids costly pairwise kernel computations and eliminates the need for multi-stage optimization pipelines. This makes SSP-BO especially attractive for scaling BO to complex, symbolic, or hybrid domains under finite-resource constraints.

RC: ***Top of page 3: "In the introduction above, we noted that the vector dot product induces an admissible kernel, or similarity function, when we represent continuous valued data using SSPs [119, 39, 23]." The dot-product between two vector-representations of data is always a positive-semidefinite function and hence an admissible kernel in the ML sense of the word. Thus, this statement is true for any embedding, be it continuous, an SSP, or otherwise, and hence seems vacuous. I believe what is meant here is that the dot-product between two VSA embeddings approximates another kernel of interest. Note as well that there is nothing preventing one from approximating a continuous kernel using discontinuous embeddings (also a widely studied topic in RFF e.g. (Raginsky and Lazebnik, 2009)) and so continuity of the embeddings doesn't seem an obviously important property here.***

AR: This statement reflects the origins of VSA work in the cognitive science community. Early VSA work focused on representing discrete symbols, with the focus on continuous-valued quantities only more recently garnering attention [138, 80], despite the capacity being introduced early in the HRR literature [199]. So it is not the continunity of the representation as much as it is the continuity of the represented data that is surprising. Further, while the reviewer is correct to point out that the kernels induced by embeddings is a well-studied problem in the machine learning literature, it is less well considered in the cognitive science community.

We have removed the quoted sentence and added a figure to illustrate the induced kernel and explain why it is interesting. The figure has the following caption:

> Fig. 2: VSA encodings and kernels for different action spaces. (a) Example action spaces used in our optimization tasks, including (i) trajectory, (ii) combinatorial, and (iii) graph-based spaces. A reference action from each space is shown: (i) a trajectory represented as waypoints connected by arrows, (ii) a combinatorial variable displayed as a color-coded grid where each square represents a categorical value, and (iii) a directed graph representing a convolutional neural network architecture, with nodes as layers (colored by operation type) and arrows as connections [160]. (b) VSA-based representations of randomly sampled actions projected into a 3D space using locally linear embedding for visualization (actual representations are high-dimensional). The black cross marks the reference action's location. (c) The induced kernel values under VSA encodings. Each point represents a randomly sampled action, with insets showing example actions (in i the reference trajectory is plotted underneath in gray and in ii overlap with the reference variable is shown). The y-axis shows cosine similarity between the VSA representation of the reference action (from a) and other actions, while the x-axis corresponds to a standard distance or similarity measure specific to each action space: (i) distance between trajectories, (ii) categorical overlap similarity, and (iii) a random walk similarity measure for graphs. These results show that VSA-based embeddings induce meaningful similarity measures aligned with known metrics in each space.

RC: ***Top of page 3: "We also showed that VSA encodings can replace kernel functions,*** $k(x, x')\phi(x) \cdot \phi(x')$ ***in the style of the kernel trick [98]." Several comments: – The citation [98] is to (Rahimi and Recht, 2008). It is weird to say "We also showed" and then to cite a paper by different authors that makes no mention of VSA. Also, this oblique reference to RFF is the only mention of the technique in the main body of the paper. FYI: per the bibliography at the end of Chapter 12 of (Wainwright, 2019), the "kernel trick" is usually attributed to (Aizerman, 1964).***
The kernel trick is explicitly about not materializing the embeddings and working with the kernel function directly. This is the opposite of what is being done here: approximating a kernel by an explicit embedding. It is another "trick" related to kernels, but it is not the kernel trick.

AR: We have changed the text (now on page 9) to "inverting the kernel trick."

RC: ***There are many others in the VSA community who have noted the connection between VSAs and kernel approximation. The earliest of which I'm aware is (Plate, 2003) who talked about his HRR as an approximate form of string kernel. The connection between VSA and RFF/Bochner's Theorem was described in (Thomas et al., 2021), and in the subsequent work of (Frady et al., 2021) who also discussed the algebraic interpretations of bundling and binding in the context of RKHS functions represented using random features. These should be mentioned in this context.***

AR: We had added the following statement to the introductory text:

> Indeed, the connection between VSAs and kernel embeddings has been well understood since their inception [118], but has garnered recent attention [151, 146, 42, 46, 80]

where references 118, 151, 146, 42, 46, and 80 are (Plate, 2003), (Voelker, 2020), (Thomas et al., 2021), (Frady et al., 2021), (Furlong et al., 2023), and (Kleyko et al., 2025) respectively. In the Section V-B4, we show the compositionality of kernels for the HRR algebra and cite the above references.

> This compliments and expands on past work connecting VSAs to kernel approximation [146] and work

> on compositionality of functions embedded in reproducing kernel Hilbert spaces, using the FHRR algebra [42]

See the updated manuscript for the full discussion.

RC: ***Top of page 3: "By approximating the kernel, we have transformed the sample selection problem into a convex optimization problem." It is not clear how this makes the sample selection problem convex. While $\mathbf{m} \cdot \phi(x)$ is linear in $\phi(x)$, it is still a nonlinear, possibly non-convex, function of x over which the optimization is defined. I believe this is related to the topic of the final bullet in this list. Please see the comment there as well.***

...

Bottom of page 14: "This means we no longer optimize in the domain X , instead we find in Rd that should correspond to the optimal point x X . We then decode by finding the point x X that maximized (x) · ." Is there a guarantee that actually is the image of x X ? Doesn't seem obvious and it would be good to explain why if so, and clarify if not. In general, it seems like (x) : x X would be a sub-manifold of Rd. Are we guaranteed that lies on this sub-manifold?

AR: First, regarding the line on page 3 – "By approximating the kernel, we have transformed the sample selection problem into a convex optimization problem" – this convexity applies only in the embedding space (and when using certain acquisition functions, e.g., UCB or MI). This does not imply convexity with respect to the original domain $x \in \mathcal{X}$. As you note, $\phi(x)$ is often a nonlinear (and potentially non-convex) function of x, and the set $\{\phi(x) : x \in \mathcal{X}\}$ defines a submanifold within $\mathbb{R}^d$. However, we are optimizing in the embedding space, so it is fair to say that it is a convex optimization. We now emphasize this point (page 7):

> By operating in the embedding space, SSP-BO transforms a complex optimization problem in a potentially structured or discrete action space into a continuous, convex optimization problem – at the cost of approximation. This optimization can be solved efficiently using simple optimization methods (e.g., gradient ascent or neural dynamics). This formulation also lends itself to neuromorphic implementation, as the same recurrent neural architecture can perform sample selection across diverse data types and structures (e.g., discrete, continuous, sets, graphs, and trajectories)

We now clarify that there is no guarantee that the optimizer ϕ^* lies on the image of the embedding manifold (page 7).

> Note that ϕ^* may not correspond exactly to any valid action embedding – *i.e.*, ϕ^* may not lie in the image of $\mathbf{x}^* \in \mathcal{X}$. Thus, we decode it by selecting the action whose embedding is most similar in feature space. This decoding step is conceptually similar to projection methods in constrained optimization, but in our case is based on similarity rather than geometric projection.

In practice, this is expected: ϕ^* will typically lie off the manifold defined by valid embedded actions. We handle this by performing a similarity-based projection (a "clean-up" step): we decode ϕ^* by finding the $x \in \mathcal{X}$ whose embedding $\phi(x)$ is most similar to ϕ^*, by maximizing the inner product $\phi(x) \cdot \phi^*$.

Off-manifold optimization followed by projection (clean-up) does not harm performance and, in fact, improves exploration. This strategy is analogous to how many latent space optimization methods operate, and we believe it is a valuable property of SSP-BO in enabling sample-efficient and scalable optimization across structured domains.

RC: ***Page 4: "In these scenarios, we test the SSP-BO algorithm against a state-of-the-art baseline, BoTorch, in three tasks relevant to robotics and machine learning." On page 6 the authors make a note of RFF as a related kernel approximation method, so it seems like this would be a salient comparison here.***

AR: We have added a RFF-BO baseline comparison for the test functions (see Figure 5).

RC: ***VSA is a broad term for a variety of different methods for systematically manipulating vector representations of data. Are the specific VSA techniques used in this paper a superset of RFF? This is a strong claim, and I'd like to see this proven as I'm not sure that it is, in fact, true.***

...

I see no reason why VSA is not also so limited. All available theory about VSAs indicates that they attain similar rates for kernel approximation as are known for RFF (Thomas et al., 2021; Clarkson et al., 2023). It's a stretch to use this to contrast the present work with RFF.

AR: This is terminology we have found challenging to pin down. On the one hand, HRRs, as we use them, are a strict subset of RFFs, because we enforce conjugate symmetry in the frequencies in order to ensure a real-valued output of the inverse fourier transfrom (see eq. 1). On the other hand, RFFs, natively, do not come equipped with the algebra that VSAs provide, even if RFFs are suitable vectors to be manipulated in the FHRR algebra. From the first perspective, the VSA we are using is a subset of RFFs, from the second, it is a superset. On the third hand, if we consider the tensor product algebras (discussed in section I-B) to be part of the RFF concept, then we return to a subset, as the dimensionality expanding nature of the TPA, which renders it incompatible with constrained computing environments, is less lossy than the dimensionalty-preserving algebra HRRs. At time of writing, we did not feel this discussion served the purpose of the paper. Based on your feedback, we have modified the statement to read:

> In kernel approximation methods, such as RFFs, compositional constructions can be obtained using concatenation and tensor products of features [e.g., 106, 147, 107, 19]. However, these operations result in feature dimensionality that grows linearly or multiplicatively with composition depth, leading to unbounded resource use as kernel complexity increases. A notable exception is the method proposed by Daniely et al. [23], which constructs product kernels using element-wise multiplication of complex-valued vectors – a strategy closely related to the FHRR binding operator. In contrast, VSA-based embeddings used in SSP-BO main- tain constant dimensionality through fixed-width algebraic operations, such as bundling and binding (a compressed tensor product). This enables scalable kernel approximation without sacrificing expressivity and supports compositional representations over arbitrary data types. Our method falls within the class of syntactic-driven kernels [51, 52], in which each structured representation induces a corresponding ker- nel function. Overall, VSAs preserve compositional expres- siveness while keeping memory and compute costs bounded – an essential property for neuromorphic implementation.

RC: ***Page 9: "Since fractional binding relates directly to the method of Random Fourier Features [98], we can appeal to that extensive literature for the design of particular kernel shapes." What does "relates directly" mean? If they are the same, just say so. If they are different, explain how, and why we can appeal to the literature of the RFF community to understand theoretical properties of the coding scheme in this paper nonetheless.***

AR: We have added the following explanation to Section V-F of the paper (this section compares SSP-BO with the new RFF-BO baseline, but includes distinctions between SSPs and RFFs specifically).

We also compare to approximate GP using RFFs, adapted from [192], which we denote as RFF-BO (see Algorithm 3). This method uses the same acquisition function and BLR updates as SSP-BO (Eq. (14) and Eq. (12), but differs in three ways, with the last two being the most significant.

- **Linear transform:** The RFFs are given by $\frac{2\sigma^2}{d}\cos(A\mathbf{x})$, where σ is fit using the initial samples. This could be obtained from an SSP constructed using A via $\frac{2\sigma^2}{d}\mathrm{Re}\ \mathcal{F}\{\phi(\mathbf{x})\}$ – in other words, it differs from an SSP by a linear transform and an additional scaling term.
- **Phase matrix generation:** The phase matrix elements, A, in the RFFs are sampled from $\mathcal{N}(0, 1\ell)$, where ℓ is optimized using the scikit-learn GP regressor with an RBF kernel over the initial samples. While in SSP-BO-Rand the elements are sampled from a uniform distribution and in SSP-BO-Hex they are generated using Algorithm 2.
- **Optimization domain:** In RFF-BO, the acquisition function is optimized over the data domain, $\max_{\mathbf{x}\in\mathcal{R}^n}\ \psi_n(\mathbf{x})$, making it a non-convex optimization problem, unlike SSP-BO which optimizes in the embedding space and then decodes (Eq. (15)).

An additional distinction is that RFF embeddings are not defined for the combinatorial, graph, or trajectory tasks.

RC: ***Top of page 12: "We appeal to the method of Random Fourier features to confirm that we can shape the kernels to our liking, as demonstrated by Frady et al. [34], Komer et al. [63], and Dumont and Eliasmith [23]." Seems like the citation here should be to the originators of RFF (Rahimi and Recht, 2008).***

AR: We have added the following text to section V-B4 of the paper:

> We appeal to the Random Fourier feature method, originated by Rahimi and Recht [124], and to demonstrations from the VSA literature by Frady et al. [34], Komer et al. [63], and Dumont and Eliasmith [23], to confirm that we can shape the kernels to our liking.

RC: ***References***

AR: These are very helpful and most have now been added to the manuscript where appropriate.

References

[13] Francis Bach. "On the equivalence between kernel quadrature rules and random feature expansions". In: *Journal of machine learning research* 18.21 (2017), pp. 1–38.

[40] Kenneth L Clarkson, Shashanka Ubaru, and Elizabeth Yang. "Capacity analysis of vector symbolic architectures". In: *arXiv preprint arXiv:2301.10352* (2023).

[80] E Paxon Frady et al. "Computing on functions using randomized vector representations". In: *arXiv preprint arXiv:2109.03429* (2021).

[138] Brent Komer et al. "A neural representation of continuous space using fractional binding." In: *CogSci*. 2019, pp. 2038–2043.

[192] Michael Osthege and Kobi Felton. *michaelosthege/pyrff: v2.0.3*. Version v2.0.3. Dec. 2020. DOI: 10.5281/zenodo.15048204. URL: https://doi.org/10.5281/zenodo.15048204.

[199] Tony A Plate. “Holographic recurrent networks”. In: *Advances in neural information processing systems* 5 (1992).

[206] Ali Rahimi, Benjamin Recht, et al. “Random Features for Large-Scale Kernel Machines.” In: *NIPS* 3.4 (2007), p. 5.

[240] Anthony Thomas, Sanjoy Dasgupta, and Tajana Rosing. “A theoretical perspective on hyperdimensional computing”. In: *Journal of Artificial Intelligence Research* 72 (2021), pp. 215–249.

Authors' Response to Reviews of

Compositional Neurosymbolic Representations Enable Efficient Active Exploration

P. Michael Furlong, Nicole S.-Y. Dumont, Rika Antonova, Jeff Orchard, Chris Eliasmith
Nature Communications,

RC: ***Reviewers' Comment***, AR: Authors' Response, ☐ Manuscript Text

Dear Editor and Reviewers,

We would like to thank the reviewers for their insightful and rigorous engagement with our work. Reviewer 4's feedback has been invaluable in helping us clarify the theoretical positioning of our manuscript.

1. Reviewer #3

Thank you for the feedback. We have made all necessary edits (these are highlighted in the new manuscript). Regarding the data, we will host it on Zenodo upon publication (so that we can include the DOI) and have edited the text to reflect this.

2. Reviewer #4

Re: VSA, Compositionality, and Algebraic Structure

RC: ***VSA is an approach for performing symbolic reasoning in neural systems, but it is instead being co-opted here simply to form an embedding for Bayesian linear regression. As such, the key ideas could actually be realized much more succinctly using standard theory. Thus, the extent to which VSA or "neurosymbolic representations" are truly an enabling component in this setting, in comparison to standard approaches, is questionable. In particular, regarding binding: Let f, g be (scalar) random feature maps for kernels K_f, K_g. Instantiate both feature maps independently. Then, it follows trivially by independence that $\mathbb{E}_{f,g}[f(x)f(x')g(y)g(y')] = \mathbb{E}_f[f(x)f(x')]\mathbb{E}_g[g(y)g(y')] = K_f(x,x')K_g(y,y')$. The analogous statement regarding sums of random features in equation 30 can be likewise derived. If the random features are not independent, then I would need to be convinced that the claims of the paper in this regard remain true in general. So it doesn't seem one need introduce any machinery related to binding to capture this idea. There is an argument to be made that the VSA framing is helpful for conceptualizing "bundles of bindings" as being useful for approximating sums of kernels by eliminating the crosstalk term. However, this idea could also have been derived as a straightforward consequence of the above (e.g. take either f or g to be the identity kernel for which there are many natural random feature maps).***

AR: We thank the reviewer for their in-depth comments. We agree that the operations within VSAs can be well understood through the lens of standard learning theory and kernel methods and we recognize that our initial presentation may have obscured our primary intent. Our goal is not to argue that VSAs provide a superior mathematical foundation for kernel theory in the abstract, but rather to demonstrate that VSAs provide a computationally efficient and "neuromorphic-friendly" means of implementing these theories.

By framing the problem through VSAs, we address three points:

- Programming Neuromorphic Hardware: VSAs can be mapped directly onto spiking neural networks and neuromorphic hardware. By showing that VSA representations can be used for Bayesian optimization, we provide a concrete pathway for deploying complex optimization tasks on low-power, neuromorphic architectures.
- Alignment with the Special Issue: As this submission is for a special issue on programming neuromorphic systems, the VSA framing is a primary contribution. Since Kanerva's 1988 work, VSAs have been positioned as a symbolic method for programming neural systems. Demonstrating this algebra's utility in Bayesian optimization, particularly with a physical hardware demonstration, addresses a significant gap in the neuromorphic literature.
- Utility of VSA Formulation: We are pleased that the reviewer notes the conceptual utility of "bundles of bindings." While these concepts can certainty be derived post-hoc with standard kernel theory, the provenance of our methodology lies firmly within the VSA literature. Further, we are unaware of

non-VSA literature that proposes such a concept or approach. The VSA framework offers a native, fixed-dimensional algebra that made the realization of these structures intuitive and computationally accessible.

Retaining this framing is essential for intellectual transparency regarding the development of the SSP-BO algorithm and for encouraging its adoption in the neurosymbolic and neuromorphic communities. We aim to enable researchers in these fields to integrate Bayesian optimization directly into existing VSA-based cognitive architectures. Text has been added to Section III-D to state this.

Nature Communications has a broad readership, and we believe the machine learning community at large stands to gain significant inspiration from the architectural constraints and representational schemes in the VSA framework. A goal of the current manuscript is to bridge disciplines and cross-pollinate ideas regarding resource-efficient computation. We have ended the conclusion with the following to highlight this:

> Beyond the specific results reported here, we hope this work illustrates a broader point: that the architectural constraints of neuromorphic and biological systems (fixed resources, compositional structure, constant-time operations) need not limit the sophistication of the algorithms they support, and that established machine learning methods can benefit from the representational frameworks developed in these communities.

RC: ***Finally, having generated our compositional feature map, the application to random-feature approximation of GP-regression is standard. In this regard, it makes no difference to the development of this paper whether ϕ is compositional or not.***

AR: The reviewer is correct that from the perspective of standard GP regression, the internal structure of ϕ is secondary to its ability to approximate a kernel. However, the value of the VSA framework lies in providing a unified, fixed-dimensional interface for optimization across disparate data structures. While the regression math remains standard, the VSA algebra allows us to map diverse spaces into a common embedding of constant dimensionality. This enables a single, fixed-size circuit to perform optimization over various domains without requiring a re-configuration of the underlying hardware or the commensurate increase in resource requirements that would come from using a non-dimensionality preserving algebra.

Compositionality is an important aspect of the feature map as it enables mixed domain representations. However, compositionality is not directly used during our optimization procedure. One could imagine an alterative approach that generates samples by composing part of past ones, but such a scheme would need to be tuned to the domain/task at hand and would not be trivial to implement on neuromorphic hardware.

RC: ***While these ideas can be cast in the language of VSA, the benefit of doing so is not made clear in this paper. Nor are the VSA notions of bundling and binding uniquely enabling here. Rather, it seems we are taking a straightforward idea that can be succinctly expressed using standard terminology familiar to a wide audience and re-casting it as "neurosymbolic," which confounds what is actually going on.***

AR: We appreciate this critique and recognize the importance of clarity in our theoretical positioning. An objective of this work is to bridge the gap between the neuromorphic communities and machine learning. To the neuromorphic and neurosymbolic communities, we are translating the powerful Bayesian optimization method into their existing toolkit. VSAs are one of the oldest neurosymbolic techniques, dating back at least to Kanerva's Sparse Distributed Memories. To the machine learning community we hope to show commonalities between their existing techniques as these methods developed in the neurocognitive community. We also hope to provide fruitful directions of future work in the space of dimensionality-preserving algebras over

vector embeddings of kernels, something useful to anyone working under hardware constraints, as we are in neuromorphics.

We have demonstrated that the VSA operations of bundling and binding are ‘enabling’ in a functional sense: they allow a single, fixed-size circuit to solve a wide variety of optimization problems across diverse spaces. We use the language of VSAs as it is the body of work that informed the development of our method and to make this work accessible/relevant to those building neurocognitive models. The reviewer’s feedback has been instrumental in helping us strike a balance between VSA-centric and standard ML framing, and we hope this clarifies that VSA is not ‘masking’ standard theory, but rather serving as a functional and architectural vehicle for it.

In our current draft, we have worked to ensure this framing complements, rather than obscures, the underlying mathematical principles. For example,

> Our method extends the applicability of kernel embeddings in several key ways, by providing an algebraic framework that realizes it within fixed computational resources, complementing standard kernel theory.

Re: Complexity

RC: ***The authors continue to push the false notion that their approach is $O(1)$ in the number of samples. As we both now agree, one is not at liberty to set d arbitrarily, independent of problem-specific concerns like the number of samples to be processed. Equation (33) even quotes a result from the RFF literature ((Rudi and Rosasco, 2017)) to that effect. So, in general, d must be chosen as a function of N , the number of samples, or some property of the underlying space/function class of interest. For example, if the algorithm requires $O(d^2)$ time, and d is $O(f(N))$ for some non-constant function f of N (and possibly other things), then the algorithm uses $O(f(N)^2)$ time. The statement that because d is fixed at runtime, we may treat this as $O(1)$ in the number of samples would be true only for a specific N which is fixed in advance, or if we elide all concerns about model accuracy. But such a statement would be true in this sense for any parametric model (e.g., LLMs with hundreds of billions of parameters) and hence vacuous.***

AR: We thank the reviewer for their continued engagement on this point. This critique has been instrumental in helping us clarify our thinking and position.

At the heart of this discussion, we believe, is the distinction between parametric and non-parametric models. We acknowledge the reviewer’s concern: the complexity of a parametric model is only $O(1)$ in the number of samples N if one assumes the model capacity d is fixed. As the reviewer correctly points out, if a problem requires $d = f(N)$ to maintain a specific error bound, the complexity is effectively $O(f(N)^2)$.

The proposition of such a function $f(N)$ suggests exciting new research directions, requiring representations that grow with experience, *i.e.*, with N. This proposition has interesting implications at the intersection of machine learning models, neurogenesis, and the constraints of currently available neuromorphic hardware. While exciting, such an effort would represent a substantial project in its own right, and is outside the scope of this paper. Absent these development of a dynamic representation and the necessary learning rules, one is left to analyze the proposed algorithm, which is theoretically constant in the number of samples, a claim that is further backed up by the empirical results seen in figures 5 b, 6 c, and 8 b. This returns us to the problem of comparing parametric and non-parametric algorithms.

We agree that this is not an "apples-to-apples" comparison; our approach trades the theoretical optimality

of a full non-parametric GP for the efficiency of a fixed-capacity parametric model. However, we believe this trade-off is precisely what makes the method valuable for the neuromorphic systems targeted by this special issue. In resource-constrained online optimization, the physical hardware provides a "hard cap" on d. At some point, the user will not be at liberty to *not* set d arbitrarily. Our contribution is demonstrating that within fixed-resource constraints, we can achieve high-performance optimization across sample sizes that exceeds the dimensionality of the embedding by several multiples.

To address these concerns and ensure total clarity for the reader, we have made the following changes:

- Removal of Unqualified Claims: We have explicitly stated the complexity claims. For example, the abstract now reads:

  > We present a Bayesian optimization algorithm that can operate in diverse complex spaces and reduces the time and memory requirements compared to state-of-the-art BO algorithms on a number of learning and robotics tasks. Our approach reduces the time and memory complexity from $O(n^3)$ and $O(n^2)$ in the number of samples, n, to $O(d^2)$ and $O(d^2)$, where $d < n$ is representation dimensionality, a constant for the algorithm's lifetime.

- Precision in Complexity Notation: In the main text, we have replaced mentions of $O(1)$ with $O(d^2)$. We explicitly state that for a pre-configured deployment, d is fixed, rendering the cost constant relative to N during runtime. However, we now explicitly acknowledge the trade-off regarding model capacity in several locations. For example,

  > We note that the embedding dimensionality d required to maintain a given error bound may grow with the number of observations for certain function classes. In many applications, including neuromorphic settings, d is fixed by hardware capacity, and one accepts a trade-off between representational fidelity and constant-time performance.

- Empirical Performance: While we cannot guarantee performance for an unbounded N, our results show that the "crosstalk" that occurs as N increases does not prevent the algorithm from finding good samples (comparable performance in GP-BO) in the tasks tested. We demonstrate a 60–200× reduction in compute time compared to standard baselines while maintaining competitive optimization performance and a significant reduction in memory growth.

We believe this improved framing (positioning SSP-BO as a fixed-resource parametric approximation) is clearer and highly relevant to the neuromorphic community, where "memory at any cost" is not an option. We thank the reviewer for helping us ensure our theoretical claims are precise.

Re: Convexity/Formulation of Optimization Problem

RC: ***Equation 15 states ... and then "decoding" that to recover*** x_n ***via equation 16.***

AR: This is a substantial improvement on our notation, thank you, we have made the modifications to equations 15 and 16 and Algorithm 1.

RC: ***Something seems off here: a convex optimization problem is generally understood to be the minimization of a convex objective (or the maximization of a concave objective). The maximizer(s) of a convex function***

are located at the extreme points of the feasible set (think about maximizing x2 over [-1, +1]) and yet the set here is unconstrained. So if this is really a convex problem, then equation (15) is not well defined as the objective is unbounded above. It is not entirely clear whether (15) is convex, concave, or neither since it is a non-trivial composition of different constituents. But something, in either the math, or the description is wrong

AR: We thank the reviewer for catching this error. First, the optimization is over the unit hypersphere (so the magnitude of θ cannot grow arbitrarily). This constraint was used in all experiments, but we did not state this in the text. We have corrected the equation to read:

$$\text{Optimize:} \quad \theta^* = \arg\max_{\theta \in \mathcal{S}^d} \mathbf{m}_{n-1}^{\mathrm{T}} \theta + \lambda \sqrt{\beta^{-1} + \theta^{\mathrm{T}} \Sigma_{n-1} \theta}, \tag{1}$$

$$\text{Decode:} \quad \mathbf{x}_n = \arg\max_{\mathbf{x} \in \mathcal{X}} \theta^* \cdot \phi(\mathbf{x}). \tag{2}$$

where $\mathcal{S}^d$ is the d-dimensional unit hypersphere.

Second, the UCB acquisition function is convex in θ (not concave) so maximizing it over $\mathcal{S}^d$ is a non-convex optimization problem. We have corrected the manuscript on pages 1, 9, 14, 19. As the reviewer points out in a later comment, the practical tractability of this optimization rests on the smoothness of the GP posterior and the compactness of the domain, not on convexity.

The revised text reads:

> By operating in the embedding space, SSP-BO transforms a complex optimization problem in a potentially structured or discrete action space, $\mathcal{X}$, into a continuous optimization problem on $\mathcal{S}^d$, at the cost of approximation. The objective is smooth and defined on a compact domain, lending itself to efficient optimization via gradient ascent or neural dynamics. This formulation also lends itself to neuromorphic implementation, as one recurrent neural architecture can perform sample selection across diverse data types and structures (*e.g.*, discrete, continuous, sets, graphs, and trajectories).

RC: ***[I]t is inaccurate to claim this is transforming a non-convex problem into a convex one. Ultimately, what we care about is x_n, which is obtained by "decoding" the output of the problem in equation (15) which, as discussed in section V-C, is, in general, non-convex. Thus, we have, at best, "convexified" only one piece, and the problem remains non convex in general.***

AR: We agree. The revised text no longer claims convexity for either stage. The benefit of the decomposition is that the latent-space optimization is continuous, smooth, and fixed-dimensional regardless of the structure of $\mathcal{X}$, making it amenable to gradient-based or neural-dynamics-based optimization. The decoding step is more complex, but approximate solutions suffice in practice. We have added language in Section II-C directing readers to Section V-C for decoding details.

Note that decoding time is included in the wall-clock run times in all experiments. Text has also been added to Section V-C stating that decoding might be bypassed in hardware implementations where motor/action systems translate embeddings directly to low-level commands rather than using Euclidean space as an intermediate representation.

RC: ***It would be helpful to provide a reference for the UCB criterion.***

AR: We have added references to Robbins 1952 (137), Lai & Robbins 1985 (99), Auer et al, 2002 (5), and Srinivas et al 2012 (154).

> *Acquisition Function:* Actions are selected by maximizing an acquisition function, $\psi_n(\mathbf{x})$. We use the Upper Confidence Bound [UCB; 137, 99, 5, 154] criterion to balance exploration and exploitation.

RC: ***It is a standard idea to apply gradient methods in non-convex settings and, while one loses guarantees about global optimality, this works well in practice on a wide variety of problems ranging from the very canonical and well studied (e.g. dictionary learning) to the contemporary (e.g. neural networks trained using SGD). So it does not seem that there is much benefit to claiming the problem is convex in any event.***

AR: We fully agree, and this is a point well taken. The revised manuscript no longer claims convexity. As the reviewer notes, gradient methods are effective in many non-convex settings, and our formulation has favourable properties that make it well-suited to such methods regardless of convexity guarantees. We believe the revised framing more accurately reflects both the problem structure and the reasons the optimization works well in practice. We thank the reviewer again for their corrections and feedback on these points.

Re: Encoding Functions and Random Fourier Features

RC: ***Equations (3) and (4) give us procedures for embedding various kinds of data without any concrete explanation of what these embeddings mean in terms of kernel approximation (which is crucial to understanding the BO formulation). That intuition comes 11 pages later in 23-30.***

AR: In order to comply with the structure and word limits of this journal, we made the decision to provide those additional methodological details in the methods section. We have added additional text to the first paragraph of Section II-A:

> Below we provide the mathematical introduction to VSAs, intuition about the kernels induced by these structures are given in Section V.

RC: ***[I]t is not clear why we need to do the inverse Fourier transform. ... So why not cut out the middle-man and just work with complex exponentials? Or, if complex numbers are bothersome, use cosine features as is done in most practical applications of RFF.***

AR: The inverse Fourier transform is to return the complex vectors to the real-domain in order to use the Holographic Reduced Representations (HRR) algebra. If we were to use complex exponentials, then we would be working with the Fourier Holographic Reduced Representations (FHRR) algorithm. A sine-cosine representation of FHRR is certainly possible, but HRR and FHRR are often considered equivalent, so the choice can be arbitrary. We have kept the inverse Fourier transform/HRR convention to reflect the current code implementation.

RC: ***After equation (3): "purely bundling variables results in an additive kernel and so, for greater expressivity (i.e. a multiplicative kernel), the mixed term is needed." It is entirely unclear what is meant by "greater expressivity."***

AR: By "greater expressivity," we refer to the capacity of the representation to approximate a broader class of kernel functions beyond simple additive models. Purely bundling variables restricts the system to an additive kernel (dimensions of the input space do not interact). By including the mixed term, we enable

the representation of multiplicative kernels, which can capture higher-order dependencies and non-linear interactions between variables. We have replaced "greater expressivity" with an explanation to clarify this:

> The first term bundles all bound slot-filler pairs, $\kappa = 0.1$ is a weighting constant, and the second term is the bound combination of all values. As discussed later in Section V-B4, purely bundling variables restricts the representation to additive kernels. To represent multiplicative kernels and capture interactions between input dimensions, the all-bound term is required.

RC: ***It is also not clear what the purpose of the "mixed" term is.***

AR: The purpose is to be able to represent products of kernels in addition to sums of kernels. We have added a footnote to explain this and have changed the language from "mixed term" to "all-bound term."

RC: ***What in (Muandet et al., 2017) suggests that the application of RFF to composite kernels results in an "unbounded" increase in dimension? Does this combine a statement about RFF with a distinct one about concatenations/tensor-products of feature maps?***

AR: The work of the Kernel Mean Embedding literature is to provide an algebraic method for composing RFF embeddings of kernels, permitting the construction of complex kernels. It is the application of the algebra specified in the KME literature to RFFs that result (not the reverse) in the unbounded increase in dimensionality. The choice of "unbounded" was to reflect the fact that if real-valued numbers were to be embedded using this technique the dimensionality would necessarily increase without bound – a limitation found in the VSA literature of Smolensky's tensor product algebra. However, the point is well taken that one need not attempt that approach using the KME algebra. We have changed the language to reflect that the algebra is not dimensionality preserving.

RC: ***There is clearly no reason why RFF has to be used in this way and the fact that a few papers have done so does not constitute a fundamental limitation on them.***

AR: This work was identified because there is a healthy body of literature on this approach, and because of the obvious parallels to the algebraic approach we use, that was developed in the cognitive science literature, and the KME approach, which was developed in the machine learning literature. We are not claiming a fundamental limitation on RFFs, only on the algebra proposed to compose them.

RC: ***Before equation 32: "Thus capacity results from the SSP literature are applicable to SSP-BO." This should be "RFF literature," the subsequent equation is a standard result from the original RFF work (which has subsequently been improved upon (Sriperumbudur and Szab´o, 2015)).***

AR: Thank you for the correction. We have changed the text to read as follows:

> Thus, capacity results from the RFF literature are applicable to SSP-BO. For example, the error of approximating a kernel is less than ϵ with high probability when
>
> $$d \geq \mathcal{O}\left(\frac{\log \operatorname{diam}(\mathcal{X})}{\epsilon^2}\right),$$
>
> where $\operatorname{diam}(\mathcal{X})$ is the diameter of the compact space that the values of $\mathbf{x}$ occupy [156,Theorem 1].

RC: ***The comparison between RFF-BO and HEX-SSP-BO conflates two things: the kernel shape and the kernel approximation method. So it seems hard to disentangle differences in the kernel approximation method (to***

the extent there are any) from the effect of the kernel choice. This possibly undercuts a useful point of novelty: that Hex-SSPs are somehow a fundamentally better choice of kernel for a certain class of BO problems. This would be interesting if so, and would have relevance for both neuroscience and ML, but it would need to be developed more.

AR: We believe that the better comparison to make is between the RFF-BO and the SSP-BO, in order to account for the difference in kernel approximation method alone. Alternatively, one might compare SSP-BO to the GP (sinc) method, the latter being the exact implementation of the former. In this comparison we see the kernel approximation method introduces a slight increase in regret. Then one can compare the difference between SSP-BO and HEX-SSP-BO to find a further improvement in performance.

Other Comments

RC: ***The illustrations in Figure 1 are more distracting than helpful in understanding. The flowchart by itself is helpful though and just focusing on that would simplify the figure.***

AR: We believe that the images demonstrating the action space and decoding are useful are concrete examples, but can agree that the images of the toroidal manifold and hexagonal basis may be distracting. We have removed those images and have made the other action images smaller.

RC: ***Subsection II-A3: aJ in the exponent: J is undefined.***

AR: We have added text to define $\mathcal{J}$ and $a_{\mathcal{J}}$.

RC: ***Section II-B: BLR is not defined until Figure 3 caption.***

AR: We have added the definition of BLR in Section II-B on the page before the Figure 3.

RC: ***After equation 16: "i.e. might not lie in the image of x X ." This should be: " might not be the image of x X ," unless you are saying that (x) is not unique. Also x does not appear to be defined anywhere, but I may have missed it.***

AR: We have clarified this text:

> θ^* might not lie in the set $\{\phi(\mathbf{x}) : \mathbf{x} \in \mathcal{X}\}$.

RC: ***At the top of section II: "The agent can observe the reward function by sampling actions from a domain x X ." This statement seems off. I understand x to be the action, and X is the domain from which it is drawn.***

AR: The text now reads:

> The agent can observe the reward function by sampling actions $\mathbf{x}$ from a domain $\mathcal{X}$, up to some maximum sampling budget.

RC: ***In section III-B: "the VSA framework naturally represents mixed discrete-continuous data in a unified space." It is a standard idea in ML to embed different types of data into a common vector space so that they can be used in a downstream learning problem. Is there something special about VSA in this regard?***

AR: What distinguishes the VSA framework is the closed algebra over the embedding space: its operations are dimensionality-preserving and compositional, and its representations are constructed rather than learned,

ensuring interpretable compositional structure by design. In standard embedding approaches, composing representations typically requires concatenation or tensor products, which grow the representation size, or learned encoders, which lack this algebraic structure.

Authors' Response to Reviews of

Compositional Neurosymbolic Representations Enable Efficient Active Exploration

P. Michael Furlong, Nicole S.-Y. Dumont, Rika Antonova, Jeff Orchard, Chris Eliasmith
Nature Communications,

RC: ***Reviewers' Comment***, AR: Authors' Response, ☐ Manuscript Text

Dear Editor and Reviewers,

Once again, we would like to thank the reviewers for their helpful commentary and rigorous critiques. We believe there is one final remark to resolve from Reviewer #2, addressed below.

RC: ***The first bullet point in the introduction (Scalability) has a typo. I think it should be as in the abstract "our approach reduces this to*** $O(d^2)$ ***and*** $O(d^2)$***, respectively".***

AR: The time complexity of the algorithm is actually order $O(d^2)$. The dominating component on time complexity is the inversion of the matrix Σ_n^{-1} in equation 14 of the paper. However, because we are only ever performing rank-one updates to the matrix, we can employ the Sherman-Morrison formula (given in equation 15) which has complexity $O(d^2)$ instead of the $O(d^3)$ of standard matrix inversion. We have added the following text to the section "Representational Capacity and Scaling Properties":

> One of the key advantages of SSP-BO is that its runtime and memory requirements remain constant with respect to the number of collected observations, n. Specifically, it avoids the $\mathcal{O}(n^3)$ time and $\mathcal{O}(n^2)$ memory costs of standard GP surrogate models, replacing them with $\mathcal{O}(d^2)$ and $\mathcal{O}(d^2)$, respectively, where d is the dimensionality of the vector embeddings. The $\mathcal{O}(d^2)$ time complexity arises from using the Sherman-Morrison formula to perform rank-one updates to Σ_n (Eq. 15). Since d is a fixed, user-selected parameter that can be chosen based on the available computational budget, and the theoretical bounds reviewed later, SSP-BO is well suited to deployment on neuromorphic hardware and other resource-constrained platforms.

Comments on: "Compositional Neurosymbolic Representations Enable Efficient Active Exploration"

Summary

The authors have substantially revised their paper in a way that improves clarity, and they have made an effort to contextualize their work alongside the broader literature on random features. However, given this context it now becomes clear that the novel contribution of this work is thin. Moreover, the way in which the approach is framed using the language of VSA is counterproductive and obscures its relation to standard methods of machine learning. Finally, the math is not sound, and the authors continue to make claims regarding O(1) sample complexity that are patently false, or at best highly misleading.

Re: VSA, Compositionality, and Algebraic Structure

As we now agree, the general idea of approximating a kernel function using a vector of random features, and using this to accelerate a downstream kernel machine (e.g. a GP as used here), is a canonical one in the broader literature. In light of this, the authors have refined the scope of their contribution in this regard to be that the "algebraic" properties of VSA provide a principled, and computationally tractable, framework for building compositional kernels out of random feature maps of various forms. VSA is an approach for performing symbolic reasoning in neural systems, but it is instead being co-opted here simply to form an embedding for Bayesian linear regression. As such, the key ideas could actually be realized much more succinctly using standard theory. Thus, the extent to which VSA or "neurosymbolic representations" are truly an enabling component in this setting, in comparison to standard approaches, is questionable.

In particular, regarding binding: Let f, g be (scalar) random feature maps for kernels K_f, K_g. Instantiate both feature maps independently. Then, it follows trivially by independence that $\mathbb{E}_{f,g}[f(x)f(x')g(y)g(y')] = \mathbb{E}_f[f(x)f(x')]\mathbb{E}_g[g(y)g(y')] = K_f(x, x')K_g(y, y')$. The analogous statement regarding sums of random features in equation 30 can be likewise derived. If the random features are not independent, then I would need to be convinced that the claims of the paper in this regard remain true in general. So it doesn't seem one need introduce any machinery related to binding to capture this idea.

There is an argument to be made that the VSA framing is helpful for conceptualizing "bundles of bindings" as being useful for approximating sums of kernels by eliminating the crosstalk term. However, this idea could also have been derived as a straightforward consequence of the above (e.g. take either f or g to be the identity kernel for which there are many natural random feature maps).

Finally, having generated our compositional feature map, the application to random-feature approximation of GP-regression is standard. In this regard, it makes no difference to the development of this paper whether ϕ is compositional or not.

While these ideas *can* be cast in the language of VSA, the benefit of doing so is not made clear in this paper. Nor are the VSA notions of bundling and binding uniquely enabling here. Rather, it seems we are taking a straightforward idea that can be succinctly expressed using standard terminology familiar to a wide audience and re-casting it as "neurosymbolic," which confounds what is actually going on.

Re: Complexity

The authors continue to push the false notion that their approach is $O(1)$ in the number of samples. As we both now agree, one is not at liberty to set d arbitrarily, independent of problem-specific concerns like the number of samples to be processed. Equation (33) even quotes a result from the RFF literature ((Rudi and Rosasco, 2017)) to that effect. So, in general, d must be chosen as a function of N, the number of samples, or some property of the underlying space/function class of interest. For example, if the algorithm requires $O(d^2)$ time, and d is $O(f(N))$ for some non-constant function f of N (and possibly other things), then the algorithm uses $O(f(N)^2)$ time. The statement that because d is fixed at runtime, we may treat this as $O(1)$ in the number of samples would be true *only for a specific N which is fixed in advance*, or if we elide all concerns about model accuracy. But such a statement would be true in this sense for any parametric model (e.g., LLMs with hundreds of billions of parameters) and hence vacuous.

Re: Convexity/Formulation of Optimization Problem

Equation 15 states:

$$\phi^* = \operatorname*{argmax}_{\phi \in \mathbb{R}^d} \psi_n(x).$$

It is totally unclear what is meant by this because in the previous exposition ϕ is a fixed embedding function. How are we optimizing over it? Additionally, the objective ψ_n is a function of x, but x is seemingly not in the domain of optimization? It seems what is meant here is that we are relaxing the domain of the problem to all of $\mathbb{R}^d$ (as opposed to $\{\phi(x) : x \in \mathcal{X}\}$) and solving:

$$\theta^* = \operatorname*{argmax}_{\theta \in \mathbb{R}^d} \boldsymbol{m}_{n-1}^T \theta + \lambda \sqrt{\beta^{-1} + \theta^\top \Sigma_{n-1} \theta},$$

and then "decoding" that to recover $\boldsymbol{x}_n$ via equation 16. Several follow up questions:

- Something seems off here: a convex optimization problem is generally understood to be the *minimization* of a convex objective (or the maximization of a concave objective). The maximizer(s) of a convex function are located at the extreme points of the feasible set (think about maximizing x^2 over $[-1, +1]$) and yet the set here is unconstrained. So if this is really a convex problem, then equation (15) is not well defined as the objective is unbounded above. It is not entirely clear whether (15) is convex, concave, or neither since it is a non-trivial composition of different constituents. But something, in either the math, or the description is wrong.

- Even absent the issues about maximizing a convex function over an unbounded domain described above, it is inaccurate to claim this is transforming a non-convex problem into a convex one. Ultimately, what we care about is x_n, which is obtained by "decoding" the output of the problem in equation (15) which, as discussed in section V-C, is, in general, non-convex. Thus, we have, at best, "convexified" only one piece, and the problem remains non convex in general.

- It would be helpful to provide a reference for the UCB criterion.

- It is a standard idea to apply gradient methods in non-convex settings and, while one loses guarantees about global optimality, this works well in practice on a wide variety of problems

ranging from the very canonical and well studied (e.g. dictionary learning) to the contemporary (e.g. neural networks trained using SGD). So it does not seem that there is much benefit to claiming the problem is convex in any event.

As it stands, the underlying problem the authors are trying to solve has not been clearly formulated and so it is hard to understand the extent to which the various approximations/relaxations done are reasonable.

Re: Encoding Functions and Random Fourier Features

Good effort has been put into expanding the discussion of encoding and how data is represented using VSA. However, the presentation is still opaque and does not provide intuition on why the VSA encoding methods used here are good ones. Equations (3) and (4) give us procedures for embedding various kinds of data without any concrete explanation of what these embeddings mean in terms of kernel approximation (which is crucial to understanding the BO formulation). That intuition comes 11 pages later in 23-30.

Several more detailed comments/questions on this point:

- In equation (1) and others stemming therefrom, it is not clear why we need to do the inverse Fourier transform. First, if we consider the standard dot-product between encodings then:

  $$\phi(s)^\top \phi(s') = \phi(s)^H \phi(s') = \exp(ia)^H F^{-H} F^{-1} \exp(ia') = \exp(ia)^H \exp(ia').$$

  So the IDFT falls out anyway when we take inner-products. Moreover, as is noted elsewhere, binding would typically be accomplished in this setting by element-wise product in the frequency domain. So why not cut out the middle-man and just work with complex exponentials? Or, if complex numbers are bothersome, use cosine features as is done in most practical applications of RFF.

- After equation (3): "purely bundling variables results in an additive kernel and so, for greater expressivity (i.e. a multiplicative kernel), the mixed term is needed." It is entirely unclear what is meant by "greater expressivity." It is also not clear what the purpose of the "mixed" term is.

- In the introduction: What in (Muandet et al., 2017) suggests that the application of RFF to composite kernels results in an "unbounded" increase in dimension? Does this combine a statement about RFF with a distinct one about concatenations/tensor-products of feature maps? There is clearly no reason why RFF has to be used in this way and the fact that a few papers have done so does not constitute a fundamental limitation on them.

- Before equation 32: "Thus capacity results from the SSP literature are applicable to SSP-BO." This should be "RFF literature," the subsequent equation is a standard result from the original RFF work (which has subsequently been improved upon (Sriperumbudur and Szabó, 2015)).

- The comparison between RFF-BO and HEX-SSP-BO conflates two things: the kernel shape and the kernel approximation method. So it seems hard to disentangle differences in the kernel approximation method (to the extent there are any) from the effect of the kernel choice. This possibly undercuts a useful point of novelty: that Hex-SSPs are somehow a fundamentally better choice of kernel for a certain class of BO problems. This would be interesting if so, and would have relevance for both neuroscience and ML, but it would need to be developed more.

Other Comments

- The illustrations in Figure 1 are more distracting than helpful in understanding. The flowchart by itself is helpful though and just focusing on that would simplify the figure.
- Subsection II-A3: a_J in the exponent: J is undefined.
- Section II-B: BLR is not defined until Figure 3 caption.
- After equation 16: "i.e. ϕ^* might not lie in the image of $x^* \in \mathcal{X}$." This should be: "ϕ^* might not *be* the image of $x^* \in \mathcal{X}$," unless you are saying that $\phi(x^*)$ is not unique. Also x^* does not appear to be defined anywhere, but I may have missed it.
- At the top of section II: "The agent can observe the reward function by sampling actions from a domain $x \in \mathcal{X}$." This statement seems off. I understand x to be the action, and $\mathcal{X}$ is the domain from which it is drawn.
- In section III-B: "the VSA framework naturally represents mixed discrete-continuous data in a unified space." It is a standard idea in ML to embed different types of data into a common vector space so that they can be used in a downstream learning problem. Is there something special about VSA in this regard?

References

Krikamol Muandet, Kenji Fukumizu, Bharath Sriperumbudur, Bernhard Schölkopf, et al. Kernel mean embedding of distributions: A review and beyond. *Foundations and Trends® in Machine Learning*, 10(1-2):1–141, 2017.

Alessandro Rudi and Lorenzo Rosasco. Generalization properties of learning with random features. *Advances in neural information processing systems*, 30, 2017.

Bharath Sriperumbudur and Zoltán Szabó. Optimal rates for random fourier features. *Advances in neural information processing systems*, 28, 2015.
